# Screening for Lipid-Metabolism-Related Genes and Identifying the Diagnostic Potential of ANGPTL6 for HBV-Related Early-Stage Hepatocellular Carcinoma

**DOI:** 10.3390/biom12111700

**Published:** 2022-11-17

**Authors:** Duo Zuo, Jiawei Xiao, Haohua An, Yongzi Chen, Jianhua Li, Xiaohui Yang, Xia Wang, Li Ren

**Affiliations:** 1 Department of Clinical Laboratory, Tianjin Medical University Cancer Institute & Hospital, Tianjin 300060, China; 2 National Clinical Research Center for Cancer, Tianjin 300060, China; 3 Tianjin’s Clinical Research Center for Cancer, Tianjin 300060, China; 4 Key Laboratory of Cancer Prevention and Therapy, Tianjin 300060, China; 5 Department of Tumor Cell Biology, Tianjin Medical University Cancer Institute & Hospital, Tianjin 300060, China; 6 Department of Gastrointestinal Medical Oncology, Tianjin Medical University Cancer Institute & Hospital, Tianjin 300060, China

**Keywords:** hepatocellular carcinoma, lipid metabolism, serum, biomarker, early diagnosis, ANGPTL6, AFP, ADH4, SLC27A5B, TTC36

## Abstract

Lipid metabolic reprogramming is one of the hallmarks of hepatocarcinogenesis and development. Therefore, lipid-metabolism-related genes may be used as potential biomarkers for hepatocellular carcinoma (HCC). This study aimed to screen for genes with dysregulated expression related to lipid metabolism in HCC and explored the clinical value of these genes. We screened differentially expressed proteins between tumorous and adjacent nontumorous tissues of hepatitis B virus (HBV)-related HCC patients using a Nanoscale Liquid Chromatography–Tandem Mass Spectrometry platform and combined it with transcriptomic data of lipid-metabolism-related genes from the GEO and HPA databases to identify dysregulated genes that may be involved in lipid metabolic processes. The potential clinical values of these genes were explored by bioinformatics online analysis tools (GEPIA, cBioPortal, SurvivalMeth, and TIMER). The expression levels of the secreted protein (angiopoietin-like protein 6, ANGPTL6) in serum were analyzed by ELISA. The ability of serum ANGPTL6 to diagnose early HCC was assessed by ROC curves. The results showed that serum ANGPTL6 could effectively differentiate between HBV-related early HCC patients with normal serum alpha-fetoprotein (AFP) levels and the noncancer group (healthy participants and chronic hepatitis B patients) (AUC = 0.717, 95% CI: from 0.614 to 0.805). Serum ANGPTL6 can be used as a potential second-line biomarker to supplement serum AFP in the early diagnosis of HBV-related HCC.

## 1. Introduction

According to global cancer statistics for 2020, liver cancer ranked seventh in incidence and second in cancer-related mortality [1]. Hepatocellular carcinoma (HCC) is the most common type of primary liver cancer, accounting for approximately 70–90% of cases [2,3]. Because HCC patients often lack symptoms in the early stages, by the time they are diagnosed, they may have missed the best time to receive surgical treatment [4]. This is one of the reasons for the poor prognosis of HCC patients [5]. Histopathological examination is the gold standard for the diagnosis of HCC, but this examination is more invasive to the patient. In addition, puncture through tissue biopsy has the potential to lead to the spread of tumor cells [6]. Heterogeneity within the tumor tissue leads to inaccurate sampling, and there is still a probability of false negative results [7,8]. Imaging and serological tests are also used to diagnose HCC. However, screening for lesions by ultrasonography may be influenced by the operator’s empirical judgment and may easily miss microscopic lesions [9,10]. Multiphasic computed tomography (CT) or magnetic resonance imaging (MRI) can improve the accuracy of HCC diagnosis but at a higher cost [11]. In addition, it has been reported that indeterminate nodules still require tissue biopsy for confirmation due to the poor performance of CT and MRI diagnosis of HCC smaller than 1 cm [12,13]. Serological tests are inexpensive and easy to standardize during the test operation. More importantly, serological testing is a noninvasive test. Therefore, this test makes it easier to obtain samples and reflects the patient’s status at the time more comprehensively, avoiding the spatial and temporal heterogeneity of tumors [7]. Serum alpha-fetoprotein (AFP) is a widely used biomarker for HCC in clinical practice. However, the application of serum AFP in the diagnosis of early-stage HCC is limited because of its low sensitivity and specificity [10,14]. Therefore, researchers have been searching for novel biomarkers to compensate for the limitations of serum AFP in diagnosing early HCC.

Chronic hepatitis B virus (HBV) infection is a major influential factor in the occurrence of HCC [15]. Especially in China, the incidence of HBV-associated HCC remains higher than that of HCC associated with other etiologies [16,17]. However, the mechanism of hepatocarcinogenesis is complex, and its mechanism is still not fully elucidated. With a better understanding of cancer, metabolic reprogramming is now considered to be one of the hallmarks of cancer [18]. In particular, abnormalities in lipid metabolism during cancerogenesis and progression are receiving increasing attention [19,20,21,22]. The reprogramming of lipid metabolism provides a large amount of energy for tumor cells and promotes their growth [19,23]. Studies have shown that HBV infection and disorders of lipid metabolism can synergistically promote the occurrence of HCC [24,25,26,27,28]. In addition, lipid-metabolism-related genes and the lipid metabolome can be used as potential biomarkers for HBV-related HCC [29,30]. However, studies on lipid metabolism are mainly focused on cardiovascular diseases and metabolic syndromes [31,32,33,34,35,36,37,38,39,40], and studies on the comprehensive analysis of lipid metabolism in HCC are still relatively few.

**Figure 1:**
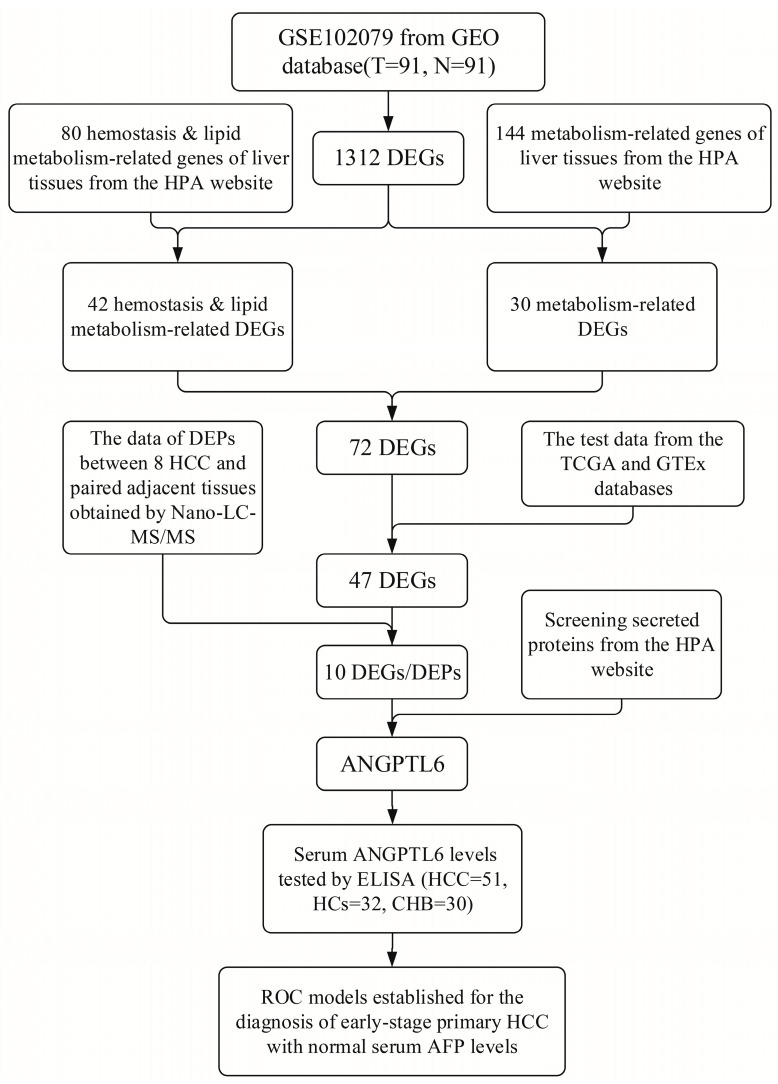
Flow chart of this study. Abbreviations: GEO, Gene Expression Omnibus; T, tumorous tissues of HCC patients; N, adjacent nontumorous tissues of HCC patients; DEGs, differentially expressed genes; HPA, Human Protein Atlas; DEPs, differential expression proteins; Nanoscale Liquid Chromatography–Tandem Mass Spectrometry, Nano-LC-MS/MS; TCGA, The Cancer Genome Atlas; GTEx, Genotype-Tissue Expression; ANGPTL6, angiopoietin-like protein 6; AFP, alpha-fetoprotein; ELISA, enzyme-linked immunosorbent assay; HCC, hepatocellular carcinoma; HCs, healthy controls; CHB, chronic hepatitis B; ROC; receiver operating characteristic.

In this study, we investigated differentially expressed proteins (DEPs) between tumorous and adjacent nontumorous tissues of HBV-related early-stage HCC patients using a Nanoscale Liquid Chromatography–Tandem Mass Spectrometry (Nano-LC-MS/MS, Thermo Fisher Scientific, Waltham, MA, USA) platform analysis and combined it with transcriptomic data of lipid-metabolism-related genes from public databases to identify genes that may be involved in dysregulated processes of lipid metabolism. The genes were evaluated for their potential clinical features at the DNA, RNA, and protein levels. More importantly, we hope to identify potential serum biomarkers that can assist AFP in the early diagnosis of HBV-related HCC. A flow chart of this study is presented in Figure 1.

## 2. Materials and Methods

### 2.1. Identification of DEGs from Microarray Data

First, we queried 18 datasets from the Gene Expression Omnibus (GEO) database on 3 September 2021 by search terms (((metabolic[All Fields] OR metabolomic[All Fields]) OR metabonomic[All Fields]) AND ((HCC[All Fields] OR (“carcinoma, hepatocellular”[MeSH Terms] OR hepatocellular carcinoma[All Fields])) OR (“liver neoplasms”[MeSH Terms] OR liver cancer[All Fields]))) AND “Homo sapiens”[porgn] AND (“gse”[Filter] AND “Expression profiling by array”[Filter] AND “attribute name tissue”[Filter]). Second, the datasets were filtered according to the following criteria: (1) primary HCC; (2) untreated patients; (3) tumorous tissues and paired adjacent nontumorous tissues from more than 50 patients; and (4) research content related to lipid metabolism. Finally, only one dataset (GSE102079 [41]) containing 91 patients met our filtering criteria. GEO2R (http://www.ncbi.nlm.nih.gov/geo/geo2r, accessed on 3 September 2021) is an online web tool that allows researchers to screen differentially expressed genes (DEGs) between HCC tumorous tissues and paired adjacent nontumorous tissues in GSE102079. The filter criteria of logFC (fold change) > 1 and adjusted *p*-value < 0.01 were used to identify the significantly upregulated DEGs. The filter criteria of logFC < −1 and adjusted *p*-value < 0.01 were used to identify the significantly downregulated DEGs. We also downloaded the data from GSE63898[42] (including 228 HCC and 168 nontumorous cirrhotic samples), GSE76427[43] (including 52 paired HCC and adjacent nontumorous cirrhotic samples), and GSE107170[44] (including 10 paired HBV-related HCC and nontumorous samples, nine paired HCV-related HCC and nontumorous samples, and five paired HDV-related HCC and nontumorous samples) to analyze ANGPTL6 expression (Table S1).

### 2.2. Functional Enrichment Analyses of DEGs

The Kyoto Encyclopedia of Genes and Genomes (KEGG) pathway and biological process functional enrichment analyses of DEGs were autogenerated by using the Database for Annotation, Visualization and Integrated Discovery (DAVID, http://david.ncifcrf.gov/, last accessed on 8 July 2022) [45]. *p* < 0.05 and false discovery rate (FDR) ≤ 0.01 were set as the thresholds [46]. Then, we selected the top 3 functional enrichment annotations with the lowest *p*-values and FDRs for visual analysis.

### 2.3. Gene Expression Clusters of Transcriptomics Data in Tissues

Gene expression clusters were obtained from the Human Protein Atlas (HPA) website (version 21, https://www.proteinatlas.org/humanproteome/tissue/expression+cluster, last accessed on 8 July 2022). According to the RNA expression data of genes in tissue samples, the protein-coding genes were clustered into gene expression clusters of various tissue types by Louvain clustering. The resulting clusters are annotated to describe common features in terms of specificity and function. This annotation is based on the overrepresentation analysis of biological databases, including Gene Ontology, Reactome, PanglaoDB, TRRUST, and KEGG pathways, as well as HPA classification, including subcellular location, proteins, secretion location, and classification, as well as specificity for human tissues, single cell types, immune cells, brain regions, and cell lines. In addition, a reliability score is set for each cluster to represent the confidence of specificity and function assignment.

### 2.4. Gene Expression Profiling Interactive Analysis (GEPIA) for Validating Gene Expression and Survival Analysis

GEPIA is an online database with an updated version (GEPIA2, http://gepia2.cancer-pku.cn, last accessed on 8 July 2022). GEPIA2 provides RNA sequencing expression data of 9736 tumors and 8587 normal samples from The Cancer Genome Atlas (TCGA) and the Genotype-Tissue Expression (GTEx) Portal, based on a standard processing pipeline [47]. In the “Expression Analysis” module, the differential expression of individual genes between liver hepatocellular carcinoma (LIHC) tissues and normal tissues was analyzed by ANOVA. RNA sequencing expression data of tumor tissues compared matched normal data from TCGA normal and GTEx data. Box plots were generated, where |Log_2_FC| greater than or equal to 1, and a *p*-value less than or equal to 0.01 were considered significant differences in the expression of the individual gene between LIHC and normal tissues. Overall survival (OS) and disease-free survival (DFS) analyses of LIHC patients were performed by Kaplan-Meier survival curves in the “Survival Analysis” module. The expression levels of a single gene higher and lower than 50% (median value) were defined as the high-expression group and the low-expression group, respectively [48]. A hazard ratio (HR) was calculated by the Cox PH mode. The 95% confidence interval (CI) as a dotted line was drawn in the survival plots. A log rank *p* < 0.05 was considered statistically significant.

### 2.5. cBioPortal for Exploring the Correlation between Gene Expression and Methylation

cBioPortal (https://www.cbioportal.org, portal version 4.1.17, last accessed on 8 July 2022) is a comprehensive public website for analyzing and visualizing multidimensional cancer genomics data [49,50]. We selected a dataset of LIHC (TCGA, Firehose Legacy), a genomic profile with mRNA expression z-scores relative to all samples (log RNA Seq V2 RSEM) (a z-score threshold of ±2.0), and an Illumina methylation (HM450) profile. Then, we analyzed the correlation between the mRNA expression z-scores of a single gene and its methylation levels from all samples with a Spearman correlation coefficient. A *p*-value < 0.05 was considered statistically significant. Absolute values of correlation coefficients were considered as follows: from 0.00 to 0.19 as very weak, from 0.20 to 0.39 as weak, from 0.40 to 0.59 as moderate, from 0.60 to 0.79 as strong, and from 0.80 to 1.0 as very strong [48]. Additionally, correlation coefficients between 0.6 and 1.0 were considered to have a strong/very strong positive correlation, and correlation coefficients between −0.6 and −1.0 were considered to have a strong/very strong negative correlation.

### 2.6. SurvivalMeth for Exploring the Correlation between DNA Methylation and the Prognosis of HCC Patients

SurvivalMeth (http://bio-bigdata.hrbmu.edu.cn/survivalmeth/, last accessed on 8 July 2022) is a web server with a collection of 81 DNA methylation profiles in 13,371 samples of 36 cancers from the TCGA, GEO, and Cancer Cell Line Encyclopedia (CCLE) databases [51]. We used the 450K (Illumina Infinium HumanMethylation450 BeadChip) experimental platform to explore the effect of DNA methylation levels of genes on the prognosis of HCC patients and selected the “samr” method for case-control differential analysis. All other options were set by default, e.g., “Threshold value” was set as “0.01”, “Absolute Difference” was set as “0”, and “Grouping Strategy” was set to “Maxstat”. The prognostic index was calculated based on the DNA methylation matrix and the coefficient obtained from the proportional hazard regression model, and patients were classified into low- and high-risk groups by the cutoff value of the prognostic index. The Kaplan–Meier plot created by sample ID, rank, survival time, patient status (alive or dead), risk group, and prognostic index was used to assess the correlation between DNA methylation levels of a single gene and the prognosis of HCC patients.

### 2.7. Tumor Immune Estimation Resource (TIMER) for Exploring the Correlation between Gene Expression and Immune Infiltration

TIMER is a web server (https://cistrome.shinyapps.io/timer/, last accessed on 8 July 2022) that encompasses 10,897 samples covering 32 cancer types from the TCGA database to assess the correlation of gene expression level (log_2_ TPM) with immune infiltration level in different cancer types [52,53]. Six types of infiltrating immune cells were used to establish these correlations, including B cells, CD4+ T cells, CD8+ T cells, macrophages, neutrophils, and dendritic cells.

### 2.8. Ethical Statement

Written informed consent was given by all participants before starting this study. Ethical approvals for this study were obtained from the Research Ethics Committee of Tianjin Medical University Cancer Institute and Hospital (protocol No. bc2020098 and No. bc2021224).

### 2.9. Tissue Sample Collection and Nano-LC-MS/MS Analysis

In our previous study, we collected eight pairs of tumorous and adjacent nontumorous tissue samples from HBV-related HCC patients with Barcelona Clinic Liver Cancer (BCLC) stage A and obtained data on the identification of proteins in these tissues by Nano-LC-MS/MS detection and analysis [30]. In the current study, we selected proteins that were highly expressed or lowly expressed in all tumorous tissues to further screen for DEPs. The conditions for screening were an FDR-adjusted *p*-value < 0.01, a fold-change value > 5 or < 0.5, and a *p*-value < 0.01. We used these data to explore whether the proteins encoded by DEGs were differentially expressed between tumorous tissues and adjacent nontumorous tissues.

### 2.10. Serum Sample Collection and Storage

HCC patients and CHB patients were recruited at the Department of Hepatobiliary of Tianjin Medical University Cancer Institute and Hospital from July 2018 to September 2022. Healthy participants (healthy controls, HCs) were enrolled at the Cancer Prevention Center of Tianjin Medical University Cancer Institute and Hospital. We defined the early stages of HCC by the BCLC staging system of stage 0-A [54,55]. Inclusion criteria were as follows: patients with untreated and histopathological diagnosis of primary HCC and cirrhosis; patients with HCC judged to be stage 0-A by BCLC staging; HCC and cirrhosis patients with chronic HBV infection (Persistent HBV infection of more than 6 months duration is defined as chronic HBV infection).; healthy participants whose physical examination report values are within the normal reference range for all indicators and who are not indicated to have any chronic diseases. Exclusion criteria were as follows: participants less than or equal to 18 years of age; patients with a history of other neoplasms; patients with a history of anti-HCC therapy or ongoing drug therapy; patients with a history of infection other than HBV infection; participants with endocrine, cardiovascular, or renal disease; participants who were breastfeeding or had a history of alcohol abuse; any factor causing abnormal elevation of serum AFP in healthy participants, such as pregnancy or any type of liver disease.

All peripheral blood samples were collected from 7:00 a.m. to 8:00 a.m. after the participants had fasted for at least 6 h. The collected tubes containing peripheral blood samples were centrifuged at 4 °C for 15 min at 3000 rpm. Next, 400 µL of serum sample was obtained from the upper layer of the tubes and stored at −80 °C until required for enzyme-linked immunosorbent assays (ELISAs). Briefly, a total of 51 serum samples from patients with early-stage HCC, 19 serum samples from CHB patients with cirrhosis, 11 serum samples from CHB patients without cirrhosis, and 32 serum samples from HCs were tested and analyzed to assess the ability of ANGPTL6 to diagnose HBV-related early-stage primary HCC.

### 2.11. ELISAs of Serum ANGPTL6

Serum ANGPTL6 levels were measured according to the manufacturer’s instructions using ELISA kits (SEN468Hu; Cloud-Clone Corp., Katy, TX, USA). The standard was reconstituted with the standard diluent. The reconstitution produced diluted standard solutions of 2^10^4^ pg/mL, 1^10^4^ pg/mL, 5000 pg/mL, 2500 pg/mL, 1250 pg/mL, 625 pg/mL, and 312 pg/mL. The standard diluent without the standard was considered a blank (standard solutions of 0 pg/mL). First, 100 µL each of the diluted standard solutions, blank, and serum samples were added to 96-well plates and incubated in the dark at 37 °C for 1 h. Then, the liquid in each well was removed. Second, 100 μL of Detection Reagent A working solution (including biotinylated ANGPTL6 antibody) was added to each well and incubated in the dark at 37 °C for 1 h. Next, 96 wells were washed, dried, and performed 3 times. Third, 100 μL of Detection Reagent B working solution (including horseradish peroxidase (HRP)-labeled avidin) was added to each well and incubated in the dark at 37 °C for 30 min. A total of 96 wells were washed, dried, and performed 5 times. After that, 90 μL of substrate solution was added to each well and incubated at 37 °C for 15 min. When the liquid was made blue by adding a substrate solution to each well, 50 μL of stop solution was added to each well to turn the liquid yellow. Finally, the optical density (O.D.) value of each well was immediately measured at 450 nm using a Multiskan FC Microplate Reader (Thermo Fisher Scientific, Waltham, MA, USA). Replicate readings for each standard and sample were averaged, and the mean value of the standard O.D. at zero concentration was subtracted. The concentrations of the standard used for creating a standard curve were 2^10^4^ pg/mL, 1^10^4^ pg/mL, 5000 pg/mL, 2500 pg/mL, 1250 pg/mL, 625 pg/mL, and 312 pg/mL. The ANGPTL6 concentration of the standard is used as the vertical coordinate, and the O.D. value as the horizontal coordinate to plot the standard curve and create the regression equation. The ANGPTL6 concentration of the serum sample was calculated via the O.D. value of the sample and the regression analysis of the standard curve. All samples and standards were assayed in duplicate. All assays were blinded to disease status.

### 2.12. ^1^H-Nuclear Magnetic Resonance (^1^H-NMR) Experiments

For the first time, we collected serum samples from 27 patients with early-stage HCC and 17 HCs (from July 2018 to December 2020) for ^1^H-NMR (Bruker 600 MHz NMR spectrometer, Bruker BioSpin, Rheinstetten, Germany) testing. The ^1^H-NMR test platform detects 112 lipoprotein subfraction indicators in serum samples, and its methodological details are the same as those described in the methods of one of our previous studies [30]. The testers were not aware of clinical information about the samples.

### 2.13. Statistical Analysis

SPSS statistical software for Windows, version 25.0 (IBM, Armonk, NY, USA) and Prism 9.0 (GraphPad Software, San Diego, CA, USA) were used for statistical analysis of the obtained data. If continuous variables were normally distributed, they were expressed as the mean ± standard deviation, and the differences between the two groups were compared using Student’s *t* tests. If continuous variables were nonnormally distributed, they were expressed as the median (interquartile range), and the differences between the two groups were compared using the Mann-Whitney U test (nonparametric analyses). Two groups of categorical variables were analyzed using chi-square tests. Correlations between serum ANGPTL6 and other laboratory indices were analyzed using Spearman’s rank correlation analysis. Spearman’s r > 0.3 or < −0.3 and *p* < 0.05 were considered to indicate a significant correlation between the two groups. The heatmap of correlations was drawn using Origin 2021 software. We used MedCalc 18.2.1 software to create receiver operating characteristic (ROC) curves to evaluate the diagnostic performances of serum ANGPTL6 level in patients with early primary HCC by the area under the ROC curves (AUCs). The median expression level of ANGPTL6 in the tissues of early HCC patients with follow-up information was the cutoff value, and early HCC patients were divided into an ANGPTL6 high expression group and an ANGPTL6 low expression group in the GSE76427 dataset. The prognostic value of ANGPTL6 levels in early HCC patients was assessed by Kaplan-Meier curves. A *p* < 0.05 (2-sided) was considered statistically significant.

## 3. Results

### 3.1. Identification of DEGs and Functional Enrichment Analyses

**Figure 2:**
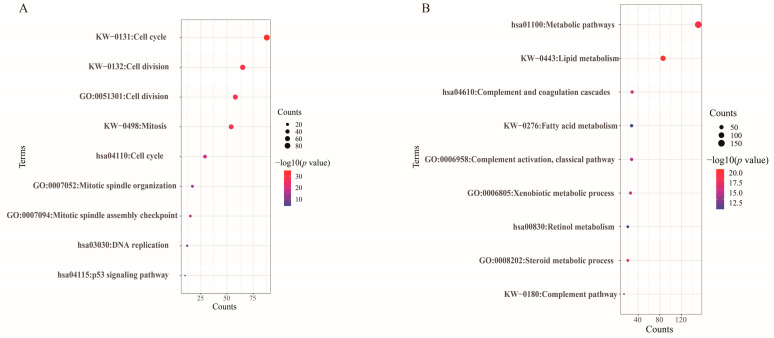
KEGG pathway and biological process enrichment analyses of 1312 DEGs from the GSE102079 dataset. (**A**) KEGG pathway and biological process enrichment analyses of 578 upregulated DEGs. (**B**) KEGG pathway and biological process enrichment analyses of 734 downregulated DEGs. In these bubble charts, the *y*-axis labels present enriched the top 3 terms of KEGG pathway, UniProt KW biological process, and GO biological process analyses. The *x*-axis labels present gene counts, which represent the number of DEGs enriched in a KEGG pathway term or a biological process term. The bubble sizes represent gene counts, and color intensity represents -log10 (*p* values) of the significantly enriched terms. *Abbreviations*: KEGG, Kyoto Encyclopedia of Genes and Genomes; KW, Keywords; GO, Gene Ontology.

Our study focused on the characterization of dysregulated gene expression involved in aberrant lipid metabolism processes in HCC, so we developed strict criteria for screening datasets in the GEO database (details of the screening are presented in the Section 2). Ultimately, we selected the GSE102079 dataset for the follow-up study. We downloaded the gene expression profiles of 91 HCC tumorous tissues and adjacent nontumorous tissues and obtained 578 upregulated DEGs and 734 downregulated DEGs in the GSE102079 dataset. Afterward, we annotated these upregulated DEGs and downregulated DEGs separately by DAVID for functional enrichment (Figure 2, Tables S2 and S3). The upregulated DEGs were significantly enriched mainly in KEGG pathways, including cell cycle, DNA replication, and p53 signaling pathways. For biological processes, these DEGs were significantly enriched, mainly in cell division and cell cycle. The downregulated DEGs were significantly enriched mainly in KEGG pathways, including metabolic pathways, complement, and coagulation cascades, and retinol metabolism. For biological processes, these DEGs were significantly enriched, mainly in lipid metabolism.

### 3.2. Identification of DEGs Related to Hemostasis and Lipid Metabolism

**Figure 3:**
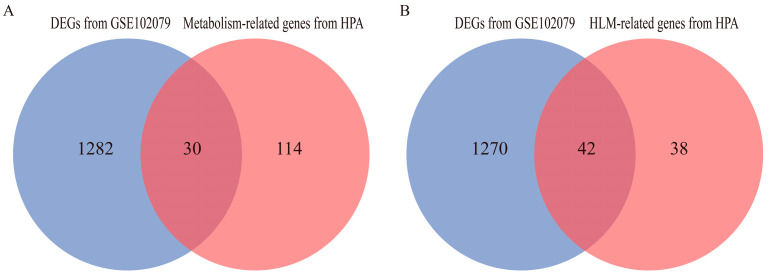
Venn diagrams of overlapping genes. (**A**) Thirty overlapping genes between 1312 DEGs (from the GSE102079 dataset) and 144 hepatic metabolism-related genes (from a gene expression cluster of the HPA website) represent hepatic metabolism-related DEGs in a Venn diagram. (**B**) Forty-two overlapping genes between 1312 DEGs (from the GSE102079 dataset) and 80 hepatic HLM-related genes (from a gene expression cluster of the HPA website) represent hepatic HLM-related DEGs in a Venn diagram.

Interestingly, the gene expression clusters of liver tissue that we found with high annotation reliabilities in the HPA website were 144 genes for metabolism-related clusters (https://www.proteinatlas.org/humanproteome/tissue/expression+cluster#cluster15, last accessed on 8 July 2022) and 80 genes for hemostasis and lipid metabolism (HLM)-related clusters (https://www.proteinatlas.org/humanproteome/tissue/expression+cluster#cluster60, last accessed on 8 July 2022). Similar results are reflected in the functional enrichment results of the 1312 DEGs of GSE102079, i.e., metabolic pathways, lipid metabolism, and coagulation cascades. Therefore, we compared these 1312 DEGs with the list of 144 genes related to hepatic metabolism and selected the overlapping genes. We obtained 30 DEGs related to hepatic metabolism (Figure 3A). In the same way, we compared these 1312 DEGs with the list of 80 genes related to hepatic HLM and selected the overlapping genes. We obtained 42 DEGs related to hepatic HLM (Figure 3B).

### 3.3. The mRNA Expression Levels of DEGs in the TCGA and GTEx Databases

We analyzed the differential expression at the transcript level of these 72 DEGs (30 DEGs related to hepatic metabolism and 42 DEGs related to hepatic HLM) between HCC tissues and normal liver tissues with RNA-Seq data from the TCGA and GTEx databases, and the expression of 47 DEGs in HCC tissues showed potentially greater robustness by comparing data from different studies. The transcript levels of these 47 DEGs were significantly differentially expressed between HCC tissues and normal liver tissues, including 19 DEGs related to hepatic metabolism and 28 DEGs related to hepatic HLM (Figures S1 and S2).

### 3.4. Identification of DEPs Encoded by the DEGs

**Figure 4:**
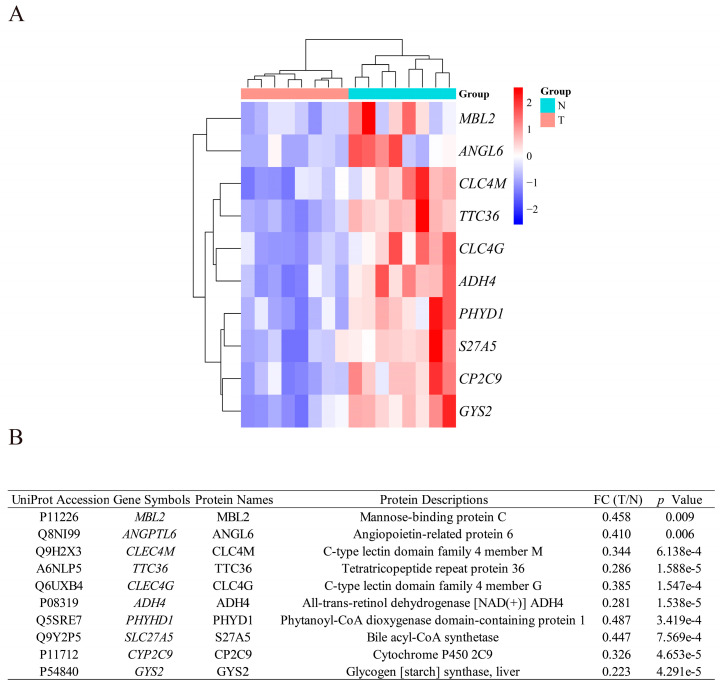
The heatmap of 10 DEPs between HCC tissues and paired adjacent nontumorous tissues. (**A**) The heatmap of 10 DEPs. (**B**) The annotated information of the DEG-encoded proteins. *Abbreviations*: *ADH4*, alcohol dehydrogenase 4 (class II), pi polypeptide; *ANGPTL6*, angiopoietin-like 6; *CLEC4G*, C-type lectin domain family 4 member G; *CLEC4M*, C-type lectin domain family 4 member M; *CYP2C9*, cytochrome P450 family 2 subfamily C member 9; *GYS2*, glycogen synthase 2; *MBL2*, mannose-binding lectin 2; *PHYHD1*, phytanoyl-CoA dioxygenase domain containing 1; *SLC27A5*, solute carrier family 27 member 5; *TTC36*, tetratricopeptide repeat domain 36; FC, fold change.

Subsequently, we obtained the data of DEPs between eight primary HCC tumorous tissues and paired adjacent nontumorous tissues by Nano-LC-MS/MS platform analysis (Clinical characteristic information of eight HCC patients is shown in Table S4). To avoid heterogeneity between the protein expression of our collection of tissues and the gene expression of tissues in public databases [56], we selected proteins that were consistent with the differential expression trends in the transcript levels of these DEGs. We finally identified that 10 of these 47 DEGs encoded proteins with significant differential expression. The expression levels of these proteins were significantly lower in HCC tissues than in nontumorous tissues (Figure 4A). The 10 DEPs encoded by the DEGs are annotated in Figure 4B. These 10 DEGs included a DEG related to hepatic metabolism [phytanoyl-CoA dioxygenase domain containing 1(*PHYHD1*)] and 9 DEGs related to hepatic HLM [mannose binding lectin 2 (*MBL2*), tetratricopeptide repeat domain 36 (*TTC36*), C-type lectin domain family 4 member G (*CLEC4G*), glycogen synthase 2 (*GYS2*), cytochrome P450 family 2 subfamily C member 9 (*CYP2C9*), C-type lectin domain family 4 member M (*CLEC4M*), alcohol dehydrogenase 4 (class II), pi polypeptide (*ADH4*), solute carrier family 27 member 5 (*SLC27A5*), and angiopoietin-like 6 (*ANGPTL6*)].

### 3.5. Exploring the Clinical Value of the 10 DEGs

**Figure 5:**
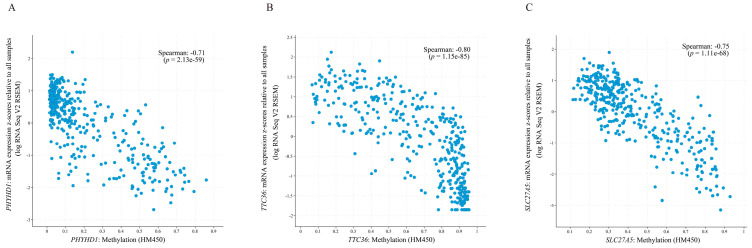
Correlations of methylation levels of (**A**) PHYHD1, (**B**) TTC36, and (**C**) SLC27A5 with corresponding mRNA levels (analyzed by the cBioPortal platform). The *y* axis shows the mRNA expression z-scores of the target gene relative to all samples. The *x* axis is the methylation level of the target gene (obtained from the HumanMethylation450 BeadChip platform). A Spearman correlation coefficient < −0.7 was considered to indicate a strong negative correlation. A *p*-value < 0.05 was considered statistically significant. Abbreviations: HM450, HumanMethylation450 BeadChip; RSEM, RNA-Seq by Expectation-Maximization.

**Figure 6:**
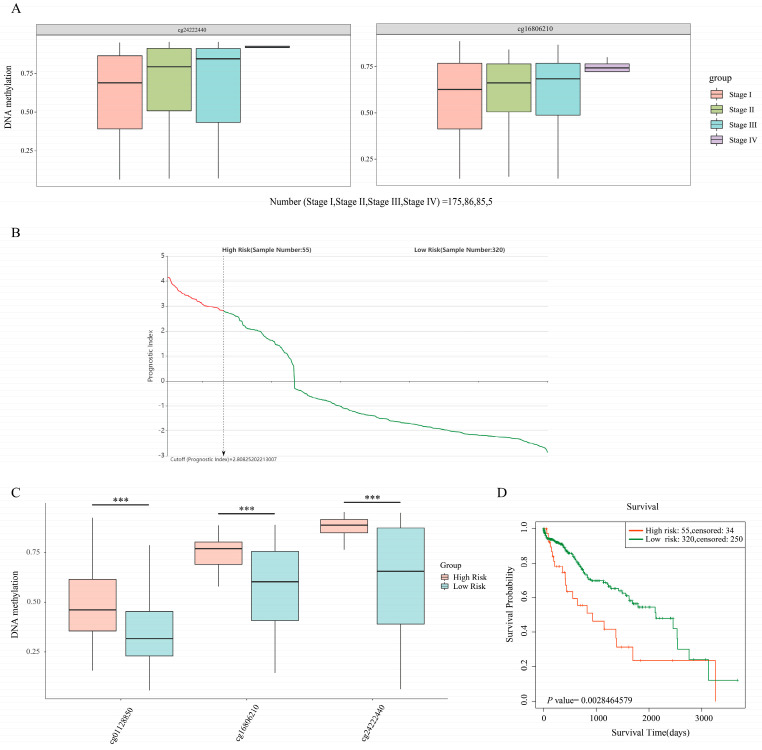
SurvivalMeth data showed a clinical correlation between TTC36 methylation levels. (**A**) Methylation levels of 2 CpGs (cg24222440 and cg16806210) of TTC36 in HCC cancer tissues with different pathological stages. (**B**) A total of 375 HCC patient samples were divided into high- and low-risk groups at the optimal cutoff (prognostic index) of 2.808. The prognostic index was calculated according to the DNA methylation matrix and the coefficient (obtained from the proportional hazards regression model) of TTC36, and a high level of prognostic index implies a high risk for the patient. The “red curve” indicates the high-risk group. The “green curve” indicates the low-risk group. (**C**) The methylation levels of 3 CpGs (cg01128850, cg16806210, and cg24222440) of TTC36 in the high- and low-risk groups. *** *p* < 0.001. (**D**) The survival curve of the Kaplan–Meier plot showed that HCC patients in the high-risk group had a lower survival probability [HR = 2.05 (1.11–3.80), *p* < 0.01].

Many studies have reported that DNA methylation can lead to the silencing of genes [57,58]. Since the mRNA expression of these 10 DEGs was downregulated in HCC tissues (Figures S1 and S2), they were analyzed for methylation correlation. We analyzed the relationship between the mRNA expression and methylation abundance of these 10 genes based on cBioPortal (Table S5). The results showed that the mRNA expression levels of *TTC36*, *CYP2C9,* and *PHYHD1* were more closely and negatively correlated with the corresponding DNA methylation levels (Figure 5 and Table S5, Spearman correlation coefficient < −0.70 and *p* < 0.05). In particular, the high methylation levels of 2 CpGs (cg24222440 and cg16806210) of *TTC36* both had a positive correlation trend with the pathologic tumor staging of HCC via SurvivalMeth automated analysis (Figure 6A). The methylation risk score model showed that the methylation levels of 3 CpGs (cg01128850, cg16806210, and cg24222440) of *TTC36* were significantly higher in the high-risk group than in the low-risk group (Figure 6B,C, *p* < 0.001), and HCC patients in the high-risk group had a shorter OS [Figure 6D, HR = 2.05 (1.11–3.80) and *p* = 0.003].

**Figure 7:**
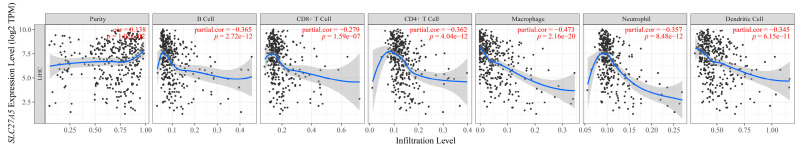
Correlation between *SLC27A5* and infiltration levels of immune cells in HCC. Correlation analysis of *SLC27A5* expression and infiltration levels of immune cells in HCC using the TIMER database. *p* < 0.05 and partial correlations < −0.3 or > 0.3 were considered to be statistically significant correlations. Abbreviations: cor, correlation. TPM, transcripts per million; TIMER, Tumor Immune Estimation Resource.

Recently, it has been demonstrated that abnormal lipid metabolism is closely related to the regulatory function of immune cells in the tumor microenvironment [59]. Therefore, we evaluated the potential relevance of these 10 genes to infiltrating immune cells using the TIMER web server (Table S6). The results showed that the expression level of *SLC27A5* was more closely and negatively correlated with the infiltration level of 6 types of immune cells (B cells, CD4+ T cells, CD8+ T cells, macrophages, neutrophils, and dendritic cells) than the other 9 DEGs (Table S6). The infiltration levels of macrophages were more closely and negatively correlated with the expression levels of *SLC27A5* than those of the other five immune cells (Figure 7 and Table S6, partial correlation < −0.4 and *p* < 0.05).

**Figure 8:**
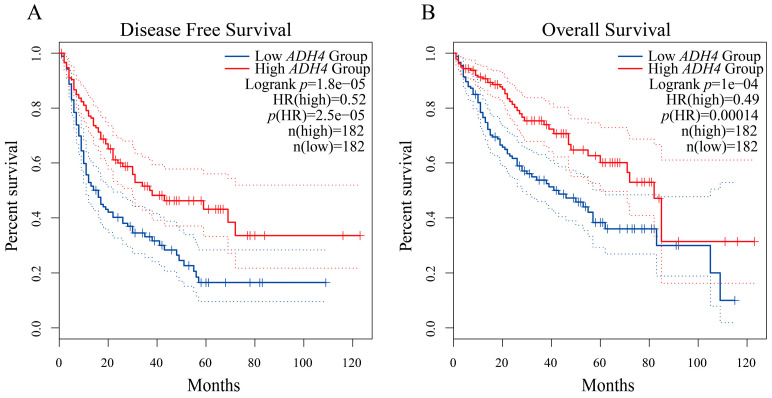
The prognostic value of *ADH4* expression for HCC patients. Kaplan-Meier survival curves were plotted using the online GEPIA platform. (**A**) Correlation of low *ADH4* expression levels with poor DFS in HCC patients. The DFS curve for patients with high *ADH4* expression levels is shown by the red solid line. The DFS curve for patients with low *ADH4* expression levels is shown by the blue solid line; (**B**) Correlation of low *ADH4* expression levels with poor OS of HCC patients. The OS curve for patients with high *ADH4* expression levels is shown by the red solid line. The OS curve for patients with low *ADH4* expression levels is shown by the blue solid line. The 95% confidence intervals are shown by the dashed line. *p* < 0.05 in the log-rank test was considered statistically significant. *Abbreviations*: DFS, disease-free survival; OS, overall survival; n, number; GEPIA, Gene Expression Profiling Interactive Analysis.

Through the GEPIA web server, we analyzed the correlation between the expression of these 10 DEGs and the prognosis of HCC patients based on the LIHC sample data in the TCGA database. The results of Kaplan-Meier survival analysis showed that lower expression of *ADH4* was significantly correlated with both poor DFS and OS among HCC patients (Figure 8, log-rank *p* < 0.05). Similarly, the expression of *CYP2C9*, *GYS2*, *SLC27A5*, and *TTC36* was significantly and positively correlated with the OS of HCC patients (Figure S3, log-rank *p* < 0.05).

To find secreted proteins in the 10 DEG-encoded DEPs between HCC tissues and adjacent nontumorous tissues and as potential circulating biomarkers for the early diagnosis of HCC, we used the HPA website to predict where these 10 DEG-encoded proteins are mainly present in the human body. We found that ANGPTL6 (https://www.proteinatlas.org/ENSG00000130812-ANGPTL6/blood+protein, accessed on 3 September 2021) and MBL2 (https://www.proteinatlas.org/ENSG00000165471-MBL2/blood+protein, accessed on 3 September 2021) may be secreted into the blood and speculated that they may have potential values in peripheral blood for the diagnosis of HCC. Given the scarcity of reports on ANGPTL6 in primary HCC, we chose ANGPTL6 for further exploration.

**Figure 9:**
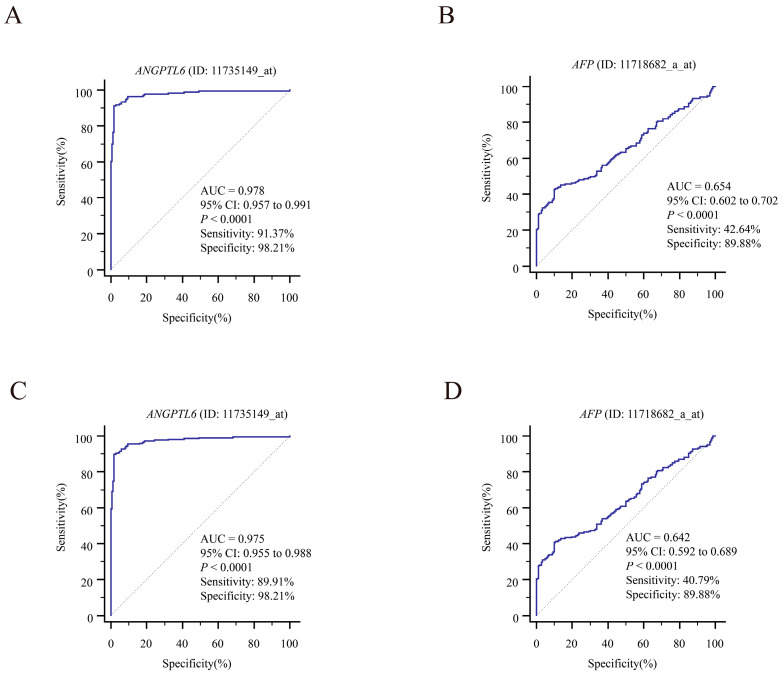
Potential diagnostic values of *ANGPTL6* and *AFP* levels for early-stage and all-stage HCC in the GSE63898 dataset. (**A**) ROC curves for *ANGPTL6* to differentiate HCC with early-stage (BCLC 0~A staging) and nontumorous cirrhotic tissues. (**B**) ROC curves for *AFP* to differentiate early-stage HCC and nontumorous cirrhotic tissues. (**C**) ROC curves for *ANGPTL6* to differentiate all-stage HCC and nontumorous cirrhotic tissues. (**D**) ROC curves for *AFP* to differentiate all-stage HCC and nontumorous cirrhotic tissues. Abbreviations: *AFP*, alpha-fetoprotein; AUC, area under the ROC curve; CI, confidence interval.

**Figure 10:**
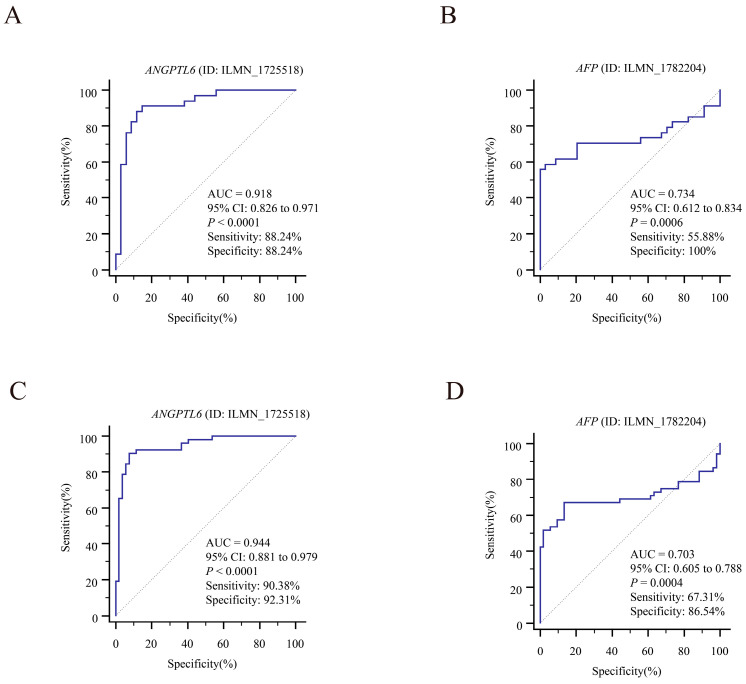
Potential diagnostic values of *ANGPTL6* and *AFP* levels for early-stage and all-stage HCC in the GSE76427 dataset. (**A**) ROC curves for *ANGPTL6* to differentiate HCC with early-stage (BCLC 0~A staging) and paired adjacent nontumorous tissues. (**B**) ROC curves for *AFP* to differentiate early-stage HCC and paired adjacent nontumorous tissues. (**C**) ROC curves for *ANGPTL6* to differentiate all-stage HCC and paired adjacent nontumorous tissues. (**D**) ROC curves for *AFP* to differentiate all-stage HCC and paired adjacent nontumorous tissues.

We downloaded GSE107170 from the GEO database and analyzed *ANGPTL6* expression in HBV-related HCC, HCV-related HCC, and HDV-related HCC tissues. *ANGPTL6* expression levels for the diagnosis of HBV-related HCC (Figure S4A), HCV-related HCC (Figure S4B), and HDV-related HCC (Figure S4C) showed great accuracies. Next, we downloaded datasets with BCLC staging information from the GEO database (GSE63898 and GSE76427). The expression level of *ANGPTL6* was effective in distinguishing HCC tissues from adjacent nontumorous tissues and was more accurate than *AFP* for diagnosis, both at early stage and at full stage of hepatocellular carcinoma (AUC*_ANGPTL6_* = 0.978 vs. AUC*_AFP_* = 0.654 for diagnosing of early HCC in the GSE63898 dataset, ROC curves are shown in Figure 9A,B) (AUC*_ANGPTL6_* = 0.975 vs. AUC*_AFP_* = 0.642 for diagnosing of HCC with all staging in the GSE63898 dataset, ROC curves are shown in Figure 9C,D) (AUC*_ANGPTL6_* = 0.918 vs. AUC*_AFP_* = 0.734 for diagnosing of early HCC in the GSE76427 dataset, ROC curves are shown in Figure 10A,B) (AUC*_ANGPTL6_* = 0.944 vs. AUC*_AFP_* = 0.703 for diagnosing of HCC with all staging in the GSE76427 dataset, ROC curves are shown in Figure 10C,D). In addition, the lower expression of *ANGPTL6* was significantly and positively correlated with the poorer recurrence-free survival of early HCC patients in the GSE76427 dataset (log-rank *p* = 0.0247) (Figure S5). In a word, the transcript levels of *ANGPTL6* in HCC tissues may have the potential to diagnose early HCC. We further explored the potential value of serum ANGPTL6 in diagnosing early HCC.

### 3.6. Serum ANGPTL6 Levels for the Diagnosis of Early Primary HCC

**Figure 11:**
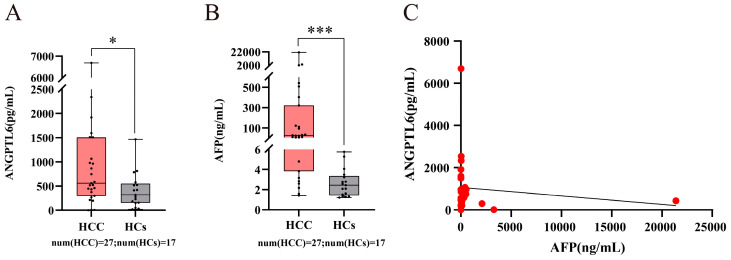
The expression levels of serum ANGPTL6 and AFP between early-stage HCC patients and HCs. (**A**) High levels of serum ANGPTL6 in early-stage HCC patients (*n* = 27) compared with those in HCs (*n* = 17) (*p* = 0.035). (**B**) High levels of serum AFP in early-stage HCC patients (*n* = 27) compared with those in HCs (*n* = 17) (*p* < 0.001). (**C**) The correlation of serum ANGPTL6 and serum AFP was analyzed using Spearman’s rank correlation analysis. * *p* < 0.05. *** *p* ≤ 0.001. Abbreviations: num, number.

In China, the etiological composition of HCC is mainly composed of HCC with chronic HBV infection [16,17], so this study was conducted with HBV-related early HCC. We collected serum samples from 32 HCs, 30 CHB patients, and 51 patients with HBV-related early-stage primary HCC (Tables S7–S9) and measured the expression levels of ANGPTL6 in serum by ELISA. We first recruited 17 HCs and 27 early-stage primary HCC patients from July 2018 to December 2020. Serum ANGPTL6 levels were significantly higher in patients with early HCC than in HCs (Figure 11A, *p* = 0.035). Serum AFP levels were also significantly higher in patients with early HCC than in HCs (Figure 11B, *p* < 0.001), but there was no significant correlation between serum ANGPTL6 levels and AFP levels in these subjects (Figure 11C, r = −0.183, *p* = 0.361).

**Figure 12:**
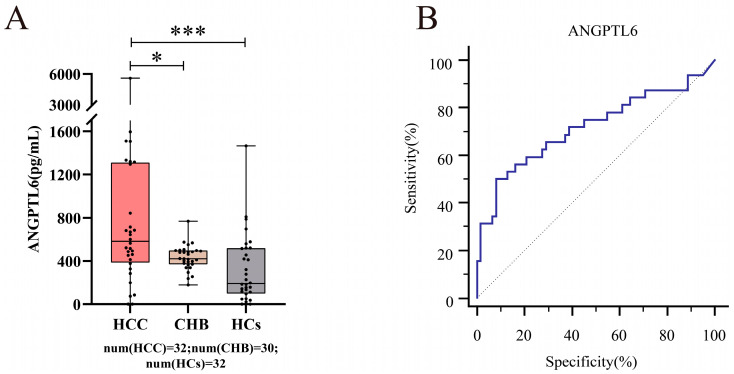
The potential value of serum ANGPTL6 for the diagnosis of early HCC patients with normal serum AFP levels (AFP ≤ 7 ng/mL). (**A**) Serum ANGPTL6 levels were higher in early HCC patients with normal serum AFP levels (*n* = 32) than in CHB patients with normal serum AFP levels (*n* = 30) (*p* = 0.013). Serum ANGPTL6 levels were higher in early HCC patients with normal serum AFP levels (*n* = 32) than in HCs (*n* = 32) (*p* = 0.001). (**B**) A ROC curve of the serum ANGPTL6 levels was used to distinguish between early HCC patients with normal serum AFP levels and the noncancer group (CHB patients and HCs). * *p* < 0.05. *** *p* ≤ 0.001.

Given that serum AFP concentration was measured by the Roche Cobas^®^ electrochemical immunoluminescence analyzer (Roche Diagnostics, Mannheim, Germany), the normal reference range for serum AFP levels is suggested in the instrument and reagent manual to be AFP ≤ 7 ng/mL [60]. We first recruited 27 early-stage primary HCC patients, of whom 8 patients had serum AFP ≤ 7 ng/mL, and then we additionally recruited 15 HCs, 19 HBV-related cirrhosis patients, 11 CHB patents (without cirrhosis), and 24 early-stage primary HCC patients with serum AFP ≤ 7 ng/mL. In total, we collected 32 serum samples with normal levels of AFP from early-stage HCC patients (Tables S8 and S9). The serum AFP levels of these HCC patients were not significantly different from those of 32 HCs (Table S8, *p* = 0.063). The serum AFP levels of these HCC patients were not significantly different from those of 30 CHB patients without HCC (including 19 HBV-related cirrhosis patients and 11 CHB patients without cirrhosis) (Table S9, *p* = 0.053). If liver abnormalities are ignored because the subject’s serum AFP level is within the normal reference range, this will result in a missed diagnosis. Therefore, we analyzed the expression levels of serum ANGPTL6 between these 32 HCC patients, 30 CHB patients, and 32 HCs, and surprisingly found that serum ANGPTL6 expression levels were significantly higher in these HCC patients than in CHB and HCs, respectively (Figure 12A and Tables S8 and S9, *p* < 0.05). Serum ANGPTL6 levels could distinguish early HCC patients with normal serum AFP levels from CHB patients (AUC = 0.684) with 96.67% specificity and could distinguish early HCC patients with normal serum AFP levels from HCs (AUC = 0.747) with 84.37% sensitivity. Furthermore, serum ANGPTL6 could effectively differentiate between the noncancer group (HCs and CHB patients) and early-stage HCC patients with serum AFP ≤ 7 ng/mL (Figure 12B and Tables S10 and S11, AUC = 0.717, 95% CI: 0.614 to 0.805) with 50.0% sensitivity and 91.9% specificity.

Therefore, we suggest that serum ANGPTL6 is a potential second-line biomarker for bridging the gap in the diagnosis of early HCC with AFP. To our knowledge, we found for the first time that serum ANGPTL6 has the potential diagnostic ability to distinguish between the noncancer group and early-stage primary HCC patients with serum AFP ≤ 7 ng/mL.

**Figure 13:**
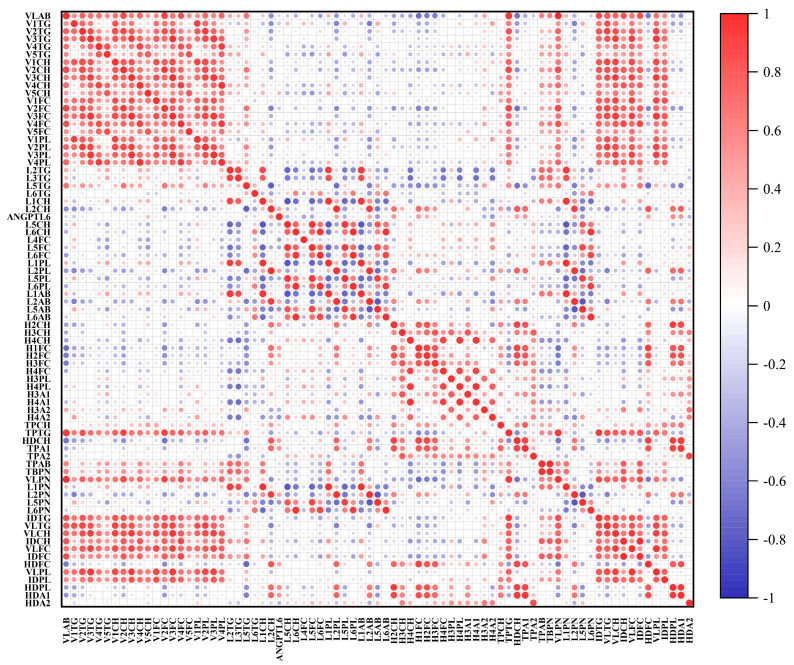
The correlation of serum ANGPTL6 and selected lipoprotein subfractions was analyzed using Spearman’s rank correlation analysis. Spearman’s rank correlation coefficient ranges from −1 to 1. The lipoprotein subfractions are annotated in Table S12.

Moreover, we examined serum samples from the first collection of 44 subjects (27 HCC patients and 17 HCs) by ^1^H-NMR, in which 76 lipoprotein subfraction levels were significantly different between the two groups (Table S12, *p* < 0.05). Serum ANGPTL6 levels in these HCC patients were significantly positively correlated with the levels of 12 lipoprotein subfractions (H2H, H1FC, H2FC, HDCH, HDFC, HDPL, HDA1, L2PN, L2CH, L2PL, L2AB, and TPA1) (Spearman’s r > 0.3, *p* < 0.05) and negatively correlated with the levels of 6 lipoprotein subfractions (L5PN, L4FC, L5FC, L5PL, L5AB, and H4A2) (Spearman’s r < −0.3, *p* < 0.05) (Figure 13 and Table S13). The lipoprotein subfractions are annotated in Table S12.

## 4. Discussion

Metabolic reprogramming processes in HCC cells are of increasing interest. The purpose of metabolic reprogramming is to provide large amounts of energy for the adaptive growth and proliferation of tumor cells [61,62,63]. Among them, lipid metabolic reprogramming is one of the key features of hepatocarcinogenesis and development [61,64,65]. The dysregulated expression of genes related to lipid metabolism is involved in the lipid metabolic reprogramming process [66,67]. In the current study, we filtered datasets from the GEO database for studies on lipid metabolism in HCC and identified many DEGs from the GSE102079 dataset. Second, some lipid-metabolism-related DEGs were screened by a gene expression profile related to hepatic lipid metabolism provided by the HPA website. Through bioinformatic analysis of DEGs at the transcription level and mass spectrometry analysis of DEGs at the protein level, 10 DEGs that could encode DEPs were finally screened, and some of them were hypothesized to be possibly associated with lipid metabolism in HCC.

By exploring the clinical characteristics of the dysregulated expression of these genes, we found that the transcript levels of *TTC36*, *SLC27A5*, and *PHYHD1* were significantly negatively correlated with their own methylation levels. In particular, the transcript level and methylation level of *TCC36* may have potential prognostic value. Our findings are consistent with previous reports. *TTC36*, a prognostic marker for HCC, is reported to encode a protein also called heat shock binding protein 21 (HBP21), which is a positive regulator of the natural antiviral immune response [68,69]. The downregulation of HBP21 in HCC is mainly caused by its allelic deletion and promoter methylation, and its upregulated expression in HCC cells can promote apoptosis [70]. It has been found that *PHYDH1* may affect the efficiency of fatty acid metabolism [71]. Although the role of *PHYHD1* in HCC is unclear, Zheng et al. [72] found low expression and high methylation levels of *PHYHD1* in HCC tissues. SLC27A5 acts as a tumor suppressor and has anti-proliferative and anti-migratory abilities in HCC cells [73,74]. It is also involved in fatty acid transport and bile acid metabolism, and its expression is downregulated by DNA hypermethylation [73]. Many reports suggest that its expression can be used as a potential marker to predict the prognosis of HCC patients [74,75,76,77]. In addition, we found that *SLC27A5* expression was significantly negatively correlated with infiltrating levels of the five immune cell types. Notably, *SLC27A5* expression was more negatively correlated with macrophage infiltration. Macrophages, as the “scavengers” in the body, are often recruited around necrotic tissues to perform the function of removing dead and dying cells [78,79]. Activated macrophages can kill tumor cells and participate in anti-tumor immunity [79]. On the other hand, tumors reprogram the metabolism of macrophages, which may lead to the conversion of macrophages to M2 subtype-like tumor-associated macrophages (TAMs) in the tumor microenvironment [78]. Qian et al. [79] described the characteristics of macrophages in the tumor microenvironment. They summarized a large amount of published experimental and clinical data and concluded that TAMs could promote tumor progression to malignancy and may promote the immune escape of tumor cells in the tumor microenvironment. Lipid accumulation has been reported in TAMs [80]. Su et al. [81] found that differentiation and activation of TAMs require enhanced lipid accumulation and metabolism. Zhang et al. [82] found that TAMs promote HCC cell migration in a fatty acid oxidation-dependent manner. Changes in the lipid profile are present not only in cancer cells but also in immune cells in the liver cancer microenvironment, which may help cancer cells better adapt to the microenvironment [59]. In future studies, we will explore whether the lipid profile changes in immune cells are associated with the dysregulation of the expression of certain genes. *ADH4*, which is considered a liver marker related to lipogenesis and lipid regulation, has an important role in the prevention of hepatic steatosis [83]. Many reports have found that low ADH4 expression is a biomarker for predicting poor prognosis in patients with HCC [84,85,86,87]. We also found that ADH4 expression was significantly and positively correlated with both OS and DFS in patients with HCC. Two DEG-encoded proteins are secreted proteins (MBL2 and ANGPTL6). Since more studies have been reported on the use of serum/plasma MBL2 for the diagnosis of HCC [88,89,90], we focused on exploring the value of serum ANGPTL6 for the diagnosis of HCC.

Serum AFP is the most widely used biomarker for diagnosing HCC [10]. One study reported that serum AFP predicted the risk of HCC in Caucasian patients with HBV-monoinfection with compensated cirrhosis on long-term tenofovir or entecavir therapy [60]. They concluded that elevated serum AFP levels above 7 ng/mL significantly predicted the development of HCC within one year in these patients but found that 69% of patients with cirrhosis who eventually developed HCC had serum AFP less than or equal to 7 ng/mL when they were diagnosed with HCC. In the current study, we enrolled a total of 32 patients with early HCC whose serum AFP levels were in the normal reference range. Serum AFP expression levels in these HCC patients were not significantly different compared to those in the HCs or the CHB patients with serum AFP ≤ 7 ng/mL. Early-stage HCC patients lack obvious symptoms [91]. When screening for HCC is performed in non-developed countries, serum AFP testing may be the only means of determining whether a subject is at risk for HCC due to the large number of people who need to be screened and the inability to afford other costly tests. However, when a patient’s serum AFP is below the upper limit of normal, he or she may not undergo further screening for HCC. These patients are most likely to miss the best time to diagnose HCC. Therefore, there is a need to find new serum biomarkers to compensate for the lack of AFP in diagnosing early HCC.

ANGPTL6 is a secreted angiogenic factor [92] that can promote endothelial cell migration and angiogenesis in AFP-producing gastric cancer [93]. ANGPTL6 also drives liver metastasis of colorectal cancer cells [94]. However, in the present study, ANGPTL6 expression was downregulated in primary HCC tissues. These results implied that ANGPTL6 may play opposite roles in primary and secondary HCC. Currently, few studies have reported the role of ANGPTL6 in the occurrence and development of primary HCC. To our knowledge, only one study found elevated expression of serum ANGPTL6 in patients with HCC compared to HCs and concluded that it has the potential as a diagnostic marker for HCC [95]. In our study, serum ANGPTL6 was found for the first time to distinguish early primary HCC patients with normal serum AFP levels from the noncancer group, with a diagnostic accuracy of 71.7%. Serum ANGPTL6 is expected to be a second-line biomarker to supplement AFP for the diagnosis of HBV-related early HCC. A study revealed that adipose tissue can secrete ANGPTL6 [96]. Mitochondrial DNA in adipocytes encodes a mitoribosomal protein, CR6-interacting factor 1, the deficiency of which leads to reduced mitochondrial oxidative phosphorylation function, which induces increased expression of the secretory factor ANGPTL6 but does not affect other angiopoietin-like proteins [96]. In addition, researchers found that leptin, as an adipokine, can lead to increased serum levels of ANGPTL6 [97]. They suggest that ANGPTL6 may act as a systemic metabolic stress marker and that elevated serum levels of ANGPTL6 may be interpreted as a compensatory upregulation of ANGPTL6 expression caused by metabolic dysregulation. Oike et al. [98] found that overexpression of ANGPTL6 in the livers of mice reversed high-fat diet-induced obesity in mice and suggested that targeted activation of ANGPTL6 in the liver could increase the energy expenditure of the body. However, there seems to be no clear mechanism to elucidate the specific role of ANGPTL6 in lipid metabolism. There is a complex interaction between the dysregulation of hepatic lipid metabolism and HBV infection, which promotes hepatocellular carcinogenesis [24,25,26,27,28]. We found that the levels of some serum lipoprotein subfractions were significantly different between patients with HBV-related early HCC and HCs, suggesting possible changes in lipid metabolism in these patients with HBV-related early HCC. Importantly, there were correlations between serum ANGPTL6 and a number of serum lipoprotein subfractions with significantly different changes in HBV-related early HCC patients, speculating that elevated serum ANGPTL6 expression in HBV-related early HCC patients may be associated with changes in lipid metabolism. Verification of this speculation will be part of our future research.

Limitations of the present study: First, the exploration of potential clinical features of lipid-metabolism-related DEGs was based on bioinformatics analysis. The specific mechanisms and clinical value of these genes involved in the reprogramming of lipid metabolism will be elucidated by clinical sample validation and cell biology studies in the future. Second, fewer clinical samples were included in exploring the diagnostic ability of serum ANGPTL6 for early HCC. In future studies, we will collect clinical samples of HCC of different etiologies to detect ANGPTL6 expression and more comprehensively analyze the differences in its expression in HCC of different etiologies. Strengths of this study: First, by ELISA of serum ANGPTL6 levels in HBV-related early primary HCC patients with normal serum AFP levels and in the noncancer group, we identified ANGPTL6 as a potential biomarker to compensate for the lack of ability of serum AFP to diagnose early HCC. Second, we found that the expression levels of serum ANGPTL6 in HCC patients were significantly correlated with the levels of some serum lipoprotein subfractions. Finally, through a combination of bioinformatics analysis of public datasets and mass spectrometry assay analysis of clinical tissue samples, we identified differential expression of *ANGPTL6*, *TTC36*, *SLC27A5*, *ADH4*, *PHYHD1*, *MBL2*, *GYS2*, *CYP2C9*, *CLEC4M,* and *CLEC4G* between HCC tissues and adjacent nontumorous tissues at the transcriptional and protein levels.

## 5. Conclusions

Overall, to our knowledge, we found for the first time that serum ANGPTL6 levels have the potential to discriminate between the noncancer group and HBV-related early primary HCC patients with normal serum AFP levels and observed that elevated serum ANGPTL6 levels in HCC patients were associated with altered levels of some lipoprotein subfractions. In addition, we aimed to screen for genes with dysregulated expression related to lipid metabolism in HCC and identified 10 DEG-encoded proteins that were differentially expressed between HCC tissues and adjacent nontumorous tissues. The potential clinical characteristics of these DEGs were evaluated by methylation level correlation analysis, immune cell infiltration level correlation analysis, and patient survival correlation analysis. We found that the high methylation level of *TTC36* was significantly and negatively correlated with its mRNA expression. *SLC27A5* expression was significantly negatively correlated with macrophage infiltration levels. Low *ADH4* expression was significantly positively correlated with poor prognosis in HCC patients. This study provides valuable reference data for future studies on mechanisms related to lipid metabolism in HCC and provides new ideas for the development of novel lipid-metabolism-related biomarkers and targeted therapies based on lipid metabolism against tumors.

## Data Availability

Publicly available data of datasets GSE102079 was analyzed in this study and can be downloaded here: https://www.ncbi.nlm.nih.gov/geo/query/acc.cgi?acc=GSE102079, accessed on 3 September 2021. Publicly available data of datasets GSE63898, GSE76427, and GSE107170 were analyzed in this study and can be downloaded here: https://www.ncbi.nlm.nih.gov/geo/query/acc.cgi?acc=GSE63898, https://www.ncbi.nlm.nih.gov/geo/query/acc.cgi?acc=GSE76427, and https://www.ncbi.nlm.nih.gov/geo/query/acc.cgi?acc=GSE107170, accessed on 11 October 2022. Public data were analyzed by GEPIA (http://gepia2.cancer-pku.cn), HPA (https://www.proteinatlas.org/), cBioPortal (https://www.cbioportal.org), SurvivalMeth (http://bio-bigdata.hrbmu.edu.cn/survivalmeth/), and TIMER (https://cistrome.shinyapps.io/timer/), accessed on 8 July 2022. In accordance with the journal’s guidelines, the data presented in this study are available on request from the corresponding author.

## Supplementary Materials

The following supporting information can be downloaded at: https://www.mdpi.com/article/10.3390/biom12111700/s1, Figure S1: The differential expression levels of 19 hepatic metabolism-related genes were validated in 369 HCC tissues and 160 normal tissues from TCGA and GTEx datasets. The tumor tissue group is shown in red, and the normal tissue group is shown in gray. |log_2_FC| > 1 and *p* < 0.01 were considered statistically significant. * *p* < 0.01. *Abbreviations:* T, tumorous tissues of HCC patients; N, nontumorous tissues of HCC patients. Figure S2: The differential expression levels of 28 hepatic HLM-related genes were validated in 369 HCC tissues and 160 normal tissues from TCGA and GTEx datasets. The tumor tissue group is shown in red, and the normal tissue group is shown in gray. |log_2_FC| > 1 and *p* < 0.01 were considered statistically significant. * *p* < 0.01. Figure S3: Correlations of *CYP2C9*, *GYS2*, *SLC27A5*, and *TTC36* expression levels with the OS of HCC patients. *p* < 0.05 in the log-rank test was considered statistically significant. Figure S4: Potential diagnostic values of *ANGPTL6* levels for three types of virus-related HCC in the GSE107170 dataset. (A) ROC curves for *ANGPTL6* to differentiate HBV-related HCC and paired adjacent nontumorous tissues. (B) ROC curves for *ANGPTL6* to differentiate HCV-related HCC and paired adjacent nontumorous tissues. (C) ROC curves for *ANGPTL6* to differentiate HDV-related HCC and paired adjacent nontumorous tissues. Figure S5: Kaplan-Meier curves for the correlation between *ANGPTL6* expression and the recurrence-free survival rate of early-stage HCC patients. The “blue curve” indicates a low expression group of *ANGPTL6* (*n* = 17). The “red curve” indicates a high expression group of *ANGPTL6* (*n* = 16). Table S1: Four datasets from the GEO database. Table S2: KEGG pathway and biological process enrichment analyses of upregulated DEGs. Table S3: KEGG pathway and biological process enrichment analyses of downregulated DEGs. Table S4: Clinical characteristic information of eight HCC patients. Table S5: Methylation analysis of 10 DEGs in LIHC by the cBioPortal platform. Table S6: Correlation analysis of the expression of 10 DEGs with the infiltration level of six immune cell types in HCC. Table S7: Characteristics of early primary HCC patients. Table S8: Characteristics of HCs and early primary HCC patients with normal serum AFP levels. Table S9: Characteristics of early primary HCC patients and CHB patients with normal serum AFP levels. Table S10: Characteristics of early primary HCC patients and the noncancer group with normal serum AFP levels. Table S11: Serum ANGPTL6 Levels for the Diagnosis of Early Primary HCC with normal serum AFP levels. Table S12: Comparison of serum lipoprotein subfraction levels in early-stage HCC patients and HCs. Table S13: Correlation analysis of serum ANGPTL6 and selected lipoprotein subfractions in patients with early-stage HCC.

## Abbreviations

^1^H-NMR	^1^H-nuclear Magnetic Resonance
ADH4	alcohol dehydrogenase 4 (class II), pi polypeptide
AFP	alpha-fetoprotein
ANGPTL6	angiopoietin-like 6
AUC	area under the ROC curve
BCLC	Barcelona Clinic Liver Cancer
CCLE	Cancer Cell Line Encyclopedia
CLEC4G	C-type lectin domain family 4 member G
CLEC4M	C-type lectin domain family 4 member M
CYP2C9	cytochrome P450 family 2 subfamily C member 9
DAVID	Database for Annotation, Visualization, and Integrated Discovery
DEGs	differentially expressed genes
DEPs	differentially expressed proteins
DFS	disease-free survival
FC	fold change
FDR	false discovery rate
GEO	Gene Expression Ominibus
GEPIA	Gene Expression Profiling Interactive Analysis
GYS2	glycogen synthase 2
HBP21	heat shock binding protein 21
HBV	hepatitis B virus
HCC	hepatocellular carcinoma
HLM	hemostasis and lipid metabolism
HM450	HumanMethylation450 BeadChip
HPA	Human Protein Atlas
HR	hazard ratio
IQR	interquartile range
KEGG	Kyoto Encyclopedia of Genes and Genomes
LIHC	liver hepatocellular carcinoma
MBL2	mannose binding lectin 2
Nano-LC-MS/MS	Nanoscale Liquid Chromatography–Tandem Mass Spectrometry
OS	overall survival
PHYHD1	phytanoyl-CoA dioxygenase domain containing 1
ROC	receiver operating characteristic
RSEM	RNA-Seq by Expectation-Maximization
SLC27A5	solute carrier family 27 member 5
TAM	tumor-associated macrophages
TCGA	The Cancer Genome Atlas
TIMER	Tumor Immune Estimation Resource
TPM	transcripts per million
TTC36	tetratricopeptide repeat domain 36
