# Supplementary material for: Screening for Lipid-Metabolism-Related Genes and Identifying the Diagnostic Potential of ANGPTL6 for HBV-Related Early-Stage Hepatocellular Carcinoma
Source: Biomolecules. 2022 Nov 17;12(11):1700. doi: 10.3390/biom12111700 (PMC9687352; doi:10.3390/biom12111700)
Supplement: Supplementary file 1 [file biomolecules-12-01700-s001.zip › Table S10.pdf]

Table S10. Characteristics of early primary HCC patients and the noncancer group with normal serum AFP levels.

**Characteristics**	**HCC Patients**	**The noncancer group**	***p*-Value**
Case	32	62	-
Age (years), Mean ± SD	61.22 ± 9.69	62.50 ± 10.59	0.569
Male, *n* (%)	29 (90.63)	56 (90.32)	0.962
HBsAg Positive, *n* (%)	32 (100)	30 (48.39)	< 0.001
HCV-Ab Positive, *n* (%)	0 (0)	0 (0)	-
HEV-IgM Positive, *n* (%)	0 (0)	0 (0)	-
BMI (kg/m^2), Median (IQR)	24.25 (23.73 to 25.28)	24.30 (23.35 to 24.93)	0.303
ALT (U/L), Median (IQR)	23.00 (14.25 to 31.50)	22.00 (17.75 to 34.00)	0.768
AST (U/L), Median (IQR)	26.00 (20.25 to 31.75)	23.00 (18.00 to 28.00)	0.063
TBIL (μmol/L), Median (IQR)	14.80 (10.15 to 20.03)	13.55 (11.40 to 20.05)	0.968
ALB (g/L), Median (IQR)	42.80 (41.15 to 44.95)	44.60 (40.98 to 46.75)	0.171
CREA (μmol/L), Median (IQR)	68.50 (59.25 to 79.50)	69.50 (65.00 to 78.50)	0.587
AFP (ng/mL), Median (IQR)	3.16 (2.26 to 4.53)	2.48 (1.71 to 3.54)	0.028
ANGPTL6 (pg/mL), Median (IQR)	582.10 (385.88 to 1309.13)	399.18 (181.34 to 497.48)	0.001

The noncancer group had a total of 30 CHB patients and 32 HCs.
