# Supplementary material for: Screening for Lipid-Metabolism-Related Genes and Identifying the Diagnostic Potential of ANGPTL6 for HBV-Related Early-Stage Hepatocellular Carcinoma
Source: Biomolecules. 2022 Nov 17;12(11):1700. doi: 10.3390/biom12111700 (PMC9687352; doi:10.3390/biom12111700)
Supplement: Supplementary file 1 [file biomolecules-12-01700-s001.zip › Table S11.pdf]

Table S11. Serum ANGPTL6 Levels for the Diagnosis of Early Primary HCC with normal serum AFP levels.

Subjects	AUC	95% CI	Sensitivity (%)	Specificity (%)	*p*-Value	The Cutoff Value of ANGPTL6 (pg/mL)
HCC Patients vs. The Noncancer Group	0.717	0.614 to 0.805	50.00	91.94	0.0005	577.5
HCC Patients vs. CHB Patients	0.684	0.554 to 0.797	50.00	96.67	0.0118	574.5
HCC Patients vs. HCs	0.747	0.623 to 0.847	84.37	59.38	0.0001	277.5
