# Supplementary material for: Screening for Lipid-Metabolism-Related Genes and Identifying the Diagnostic Potential of ANGPTL6 for HBV-Related Early-Stage Hepatocellular Carcinoma
Source: Biomolecules. 2022 Nov 17;12(11):1700. doi: 10.3390/biom12111700 (PMC9687352; doi:10.3390/biom12111700)
Supplement: Supplementary file 1 [file biomolecules-12-01700-s001.zip › Table S13.pdf]

Table S13. Correlation analysis of serum ANGPTL6 and selected lipoprotein subfractions in patients with early-stage HCC.

Index	Spearman's r	*p*
TPCH	0.256	0.197
TPTG	-0.182	0.364
HDCH	0.478*	0.012
TPA1	0.509**	0.007
TPA2	-0.121	0.546
TPAB	-0.176	0.380
TBPN	-0.176	0.380
VLPN	-0.216	0.279
L1PN	0.109	0.590
L2PN	0.441*	0.021
L5PN	-0.483*	0.011
L6PN	-0.120	0.550
IDTG	-0.015	0.940
VLTG	-0.194	0.332
VLCH	0.040	0.844
IDCH	-0.134	0.506
VLFC	0.085	0.673
IDFC	-0.163	0.417
HDFC	0.440*	0.022
VLPL	0.063	0.755
IDPL	0.134	0.504
HDPL	0.579**	0.002
HDA1	0.529**	0.005
HDA2	-0.351	0.073
VLAB	-0.216	0.279
V1TG	-0.277	0.162

V2TG	-0.142	0.481
V3TG	0.047	0.816
V4TG	0.033	0.870
V5TG	0.059	0.769
V1CH	-0.071	0.726
V2CH	-0.079	0.694
V3CH	0.080	0.691
V4CH	0.148	0.463
V5CH	0.346	0.077
V1FC	0.152	0.449
V2FC	-0.265	0.182
V3FC	0.053	0.795
V4FC	0.002	0.993
V5FC	0.049	0.808
V1PL	-0.162	0.418
V2PL	-0.031	0.880
V3PL	0.154	0.442
V4PL	0.100	0.619
L2TG	0.121	0.549
L3TG	0.082	0.683
L5TG	-0.227	0.256
L6TG	-0.204	0.307
L1CH	0.085	0.675
L2CH	0.462*	0.015
L5CH	-0.328	0.094
L6CH	-0.167	0.406
L4FC	-0.439*	0.022
L5FC	-0.415*	0.032
L6FC	-0.120	0.552

L1PL	0.110	0.585
L2PL	0.468*	0.014
L5PL	-0.386*	0.047
L6PL	-0.150	0.456
L1AB	0.109	0.590
L2AB	0.441*	0.021
L5AB	-0.483*	0.011
L6AB	-0.120	0.550
H2CH	0.514**	0.006
H3CH	0.070	0.728
H4CH	-0.173	0.389
H1FC	0.473*	0.013
H2FC	0.402*	0.037
H3FC	0.248	0.212
H4FC	-0.080	0.691
H3PL	0.010	0.961
H4PL	-0.132	0.512
H3A1	0.085	0.673
H4A1	-0.076	0.705
H3A2	-0.373	0.055
H4A2	-0.501**	0.008

*$p < 0.05$ (2-sided) and Spearman's r > 0.3 or < -0.3 were considered to be statistically significant correlations. **$p < 0.01$ (2-sided) and Spearman's r > 0.3 or < -0.3 were considered to be statistically significant correlations. The lipoprotein subfractions are annotated in Table S12.
