# Supplementary material for: Screening for Lipid-Metabolism-Related Genes and Identifying the Diagnostic Potential of ANGPTL6 for HBV-Related Early-Stage Hepatocellular Carcinoma
Source: Biomolecules. 2022 Nov 17;12(11):1700. doi: 10.3390/biom12111700 (PMC9687352; doi:10.3390/biom12111700)
Supplement: Supplementary file 1 [file biomolecules-12-01700-s001.zip › Table S2.pdf]

Table S2. KEGG pathway and biological process enrichment analyses of up-regulated DEGs.

**Term**	**Description**	**Gene Count**	***p*-Value**	**FDR**
KEGG Pathway				
hsa04110	Cell cycle	29	2.63e-19	7.19e-17
hsa03030	DNA replication	12	7.07e-10	9.65e-08
hsa04115	p53 signaling pathway	10	8.41e-5	0.008
Biological Process				
KW-0131	Cell cycle	88	1.07e-35	1.02e-33
KW-0132	Cell division	65	7.53e-31	3.58e-29
KW-0498	Mitosis	54	2.50e-29	7.93e-28
GO: 0051301	Cell division	58	4.07e-29	9.61e-26
GO: 0007094	Mitotic spindle assembly checkpoint	15	4.98e-15	5.88e-12
GO: 0007052	Mitotic spindle organization	17	1.46e-13	1.15e-10

Abbreviations: KEGG, Kyoto Encyclopedia of Genes and Genomes; KW, Keywords; GO, Gene Ontology; DEGs, differentially expressed genes; FDR, false discovery rate.
