# Supplementary material for: Screening for Lipid-Metabolism-Related Genes and Identifying the Diagnostic Potential of ANGPTL6 for HBV-Related Early-Stage Hepatocellular Carcinoma
Source: Biomolecules. 2022 Nov 17;12(11):1700. doi: 10.3390/biom12111700 (PMC9687352; doi:10.3390/biom12111700)
Supplement: Supplementary file 1 [file biomolecules-12-01700-s001.zip › Table S3.pdf]

Table S3. KEGG pathway and biological process enrichment analyses of down-regulated DEGs.

Term	Description	Gene Count	*p*-Value	FDR
KEGG Pathway				
hsa01100	Metabolic pathways	151	7.72e-19	2.09e-16
hsa04610	Complement and coagulation cascades	29	1.99e-16	2.70e-14
hsa00830	Retinol metabolism	21	5.00e-11	4.48e-09
Biological Process				
KW-0443	Lipid metabolism	84	8.12e-21	7.80e-19
KW-0180	Complement pathway	14	3.46e-11	1.66e-09
KW-0276	Fatty acid metabolism	28	8.59e-11	2.75e-09
GO: 0008202	Steroid metabolic process	21	1.98e-16	6.36e-13
GO: 0.006805	Xenobiotic metabolic process	26	1.45e-15	2.33e-12
GO: 0.006958	Complement activation, classical pathway	28	7.72e-15	8.26e-12

Abbreviations: KEGG, Kyoto Encyclopedia of Genes and Genomes; KW, Keywords; GO, Gene Ontology; DEGs, differentially expressed genes; FDR, false discovery rate.
