# Supplementary material for: Screening for Lipid-Metabolism-Related Genes and Identifying the Diagnostic Potential of ANGPTL6 for HBV-Related Early-Stage Hepatocellular Carcinoma
Source: Biomolecules. 2022 Nov 17;12(11):1700. doi: 10.3390/biom12111700 (PMC9687352; doi:10.3390/biom12111700)
Supplement: Supplementary file 1 [file biomolecules-12-01700-s001.zip › Table S5.pdf]

Table S5. Methylation analysis of 10 DEGs in LIHC by cBioPortal platform.

Genes	Correlation of Methylation Levels of Genes and Their mRNA Levels
ADH4	Spearman: -0.24 (p = 2.89e-6)
ANGPTL6	Spearman: -0.28 (p = 3.43e-8)
CLEC4G	Spearman: 0.03 (p =0.629)
CLEC4M	Spearman: 0.16 (p =1.69e-3)
CYP2C9	Spearman: -0.58 (p = 5.82e-35)
GYS2	Spearman: -0.53 (p = 2.87e-28)
MBL2	Spearman: -0.46 (p = 1.49e-20)
PHYHD1	Spearman: -0.71 (p = 2.13e-59)
SLC27A5	Spearman: -0.75 (p = 1.11e-68)
TTC36	Spearman: -0.80 (p = 1.15e-85)

Abbreviations: DEGs, differentially expressed genes; LIHC, liver hepatocellular carcinoma; *ADH4*, alcohol dehydrogenase 4 (class II), pi polypeptide; *ANGPTL6*, angiopoietin like 6; *CLEC4G*, C-type lectin domain family 4 member G; *CLEC4M*, C-type lectin domain family 4 member M; *CYP2C9*, cytochrome P450 family 2 subfamily C member 9; *GYS2*, glycogen synthase 2; *MBL2*, mannose binding lectin 2; *PHYHD1*, phytanoyl-CoA dioxygenase domain containing 1; *SLC27A5*, solute carrier family 27 member 5; *TTC36*, tetratricopeptide repeat domain 36.
