# Supplementary material for: Screening for Lipid-Metabolism-Related Genes and Identifying the Diagnostic Potential of ANGPTL6 for HBV-Related Early-Stage Hepatocellular Carcinoma
Source: Biomolecules. 2022 Nov 17;12(11):1700. doi: 10.3390/biom12111700 (PMC9687352; doi:10.3390/biom12111700)
Supplement: Supplementary file 1 [file biomolecules-12-01700-s001.zip › Table S6.pdf]

**Table S6.** Correlation analysis of the expression of 10 DEGs with the infiltration level of six immune cell types in HCC.

| Genes | B Cells | | CD8+ T Cells | | CD4+ T Cells | | Macrophages | | Neutrophils | | Dendritic Cells | |
|---|---|---|---|---|---|---|---|---|---|---|---|---|
| | *p*-Value | Partial Correlation | *p*-Value | Partial Correlation | *p*-Value | Partial Correlation | *p*-Value | Partial Correlation | *p*-Value | Partial Correlation | *p*-Value | Partial Correlation |
| *ADH4* | 2.82e-08 | -0.294 | 1.52e-04 | -0.203 | 1.72e-04 | -0.201 | 1.43e-08 | -0.301 | 2.30e-05 | -0.226 | 1.91e-05 | -0.230 |
| *ANGPTL6* | 1.35e-04 | -0.204 | 0.024 | -0.122 | 0.902 | 0.007 | 0.119 | -0.085 | 0.496 | -0.037 | 0.114 | -0.086 |
| *CLEC4G* | 0.477 | 0.038 | 0.110 | 0.086 | 0.009 | 0.142 | 8.78e-05 | 0.211 | 0.086 | 0.092 | 0.174 | 0.074 |
| *CLEC4M* | 0.702 | 0.021 | 0.538 | 0.033 | 0.129 | 0.082 | 0.345 | 0.051 | 0.869 | 0.009 | 0.804 | -0.013 |
| *CYP2C9* | 3.31e-04 | -0.192 | 0.009 | -0.141 | 7.48e-04 | -0.181 | 2.52e-07 | -0.275 | 0.004 | -0.154 | 0.002 | -0.166 |
| *GYS2* | 7.78e-09 | -0.305 | 1.21e-05 | -0.234 | 2.95e-04 | -0.194 | 1.10e-07 | -0.283 | 0.011 | -0.136 | 3.30e-07 | -0.273 |
| *MBL2* | 1.86e-04 | -0.200 | 4.72e-05 | -0.218 | 0.291 | -0.057 | 1.81e-04 | -0.201 | 0.011 | -0.137 | 7.13e-04 | -0.183 |
| *PHYHD1* | 5.79e-06 | -0.242 | 0.064 | -0.100 | 0.025 | -0.121 | 6.52e-05 | -0.215 | 0.078 | -0.095 | 0.009 | -0.142 |
| *SLC27A5* | 2.72e-12 | -0.365 | 1.59e-07 | -0.279 | 4.04e-12 | -0.362 | 2.16e-20 | -0.473 | 8.48e-12 | -0.357 | 6.15e-11 | -0.345 |
| *TTC36* | 3.99e-12 | -0.363 | 3.10e-04 | -0.194 | 3.83e-07 | -0.270 | 3.00e-12 | -0.366 | 6.80e-06 | -0.240 | 1.29e-07 | -0.282 |

$p < 0.05$ and partial correlations < -0.3 or > 0.3 were considered to be statistically significant correlations.
