# Supplementary material for: Screening for Lipid-Metabolism-Related Genes and Identifying the Diagnostic Potential of ANGPTL6 for HBV-Related Early-Stage Hepatocellular Carcinoma
Source: Biomolecules. 2022 Nov 17;12(11):1700. doi: 10.3390/biom12111700 (PMC9687352; doi:10.3390/biom12111700)
Supplement: Supplementary file 1 [file biomolecules-12-01700-s001.zip › Table S7.pdf]

Table S7. Characteristics of early primary HCC patients.

Characteristics	HCC Patients
Case, *n*	51
Age (years), Mean ± SD	60.78 ± 9.11
Male, *n* (%)	47 (92.16)
Tumor size (cm)	
⩾ 3 cm, *n* (%)	30 (58.82)
< 3 cm, *n* (%)	21 (41.18)
< 2 cm, *n* (%)	11 (21.57)
< 1 cm, *n* (%)	2 (3.92)
Tumor Location in Couinaud's Segment	
I, *n* (%)	3 (5.88)
II, *n* (%)	4 (7.84)
III, *n* (%)	2 (3.92)
IV, *n* (%)	5 (9.80)
V, *n* (%)	8 (15.69)
VI, *n* (%)	8 (15.69)
VII, *n* (%)	13 (25.50)
VIII, *n* (%)	4 (7.84)
II and IV, *n* (%)	1 (1.96)
VI and VII, *n* (%)	2 (3.92)
VII and VIII, *n* (%)	1 (1.96)
Differentiation Degree	
Well, *n* (%)	5 (9.80)
Moderate, *n* (%)	25 (49.02)
Poor, *n* (%)	21 (41.18)
Child-Push Class	
A, *n* (%)	47 (92.16)
B, *n* (%)	4 (7.84)
Cirrhosis, *n* (%)	35 (68.63)
BCLC Stage	
Stage 0, *n* (%)	5 (9.80)
Stage A, *n* (%)	46 (90.20)
HBsAg Positive, *n* (%)	51 (100)
HCV-Ab Positive, *n* (%)	0 (0)
HEV-IgM Positive, *n* (%)	0 (0)
BMI (kg/m^2), Median (IQR)	24.1 (23.7 to 24.8)
ALT (U/L), Median (IQR)	24.0 (19.0 to 35.0)
AST (U/L), Median (IQR)	26.0 (21.0 to 33.0)
TBIL (μmol/L), Median (IQR)	15.2 (11.0 to 21.7)
ALB (g/L), Median (IQR)	42.8 (41.0 to 45.0)
PT (sec), Median (IQR)	11.5 (11.0 to 11.9)
CREA (μmol/L), Median (IQR)	69.0 (59.0 to 80.0)
AFP (ng/mL), Median (IQR)	4.74 (3.02 to 28.27)

AFP > 7 ng/mL, *n* (%)	19 (37.25)
AFP > 20 ng/mL, *n* (%)	15 (29.41)
AFP > 200 ng/mL, *n* (%)	7 (13.73)
AFP > 400 ng/mL, *n* (%)	6 (11.76)

Abbreviations: SD, standard deviation; IQR, interquartile range.
