# Supplementary material for: Screening for Lipid-Metabolism-Related Genes and Identifying the Diagnostic Potential of ANGPTL6 for HBV-Related Early-Stage Hepatocellular Carcinoma
Source: Biomolecules. 2022 Nov 17;12(11):1700. doi: 10.3390/biom12111700 (PMC9687352; doi:10.3390/biom12111700)
Supplement: Supplementary file 1 [file biomolecules-12-01700-s001.zip › Table S8.pdf]

Table S8. Characteristics of HCs and early primary HCC patients with normal serum AFP levels.

**Characteristics**	**HCC Patients**	**HCs**	***p*-Value**
Case	32	32	-
Age (years), Mean ± SD	61.22 ± 9.69	65.75 ± 9.67	0.066
Male, *n* (%)	29 (90.63)	30 (93.75)	0.641
HBsAg Positive, *n* (%)	32 (100)	0 (0)	-
HCV-Ab Positive, *n* (%)	0 (0)	0 (0)	-
HEV-IgM Positive, *n* (%)	0 (0)	0 (0)	-
BMI (kg/m^2), Median (IQR)	24.25 (23.73 to 25.28)	24.30 (23.80 to 25.05)	0.957
ALT (U/L), Median (IQR)	23.00 (14.25 to 31.50)	22.50 (18.00 to 33.00)	0.747
AST (U/L), Median (IQR)	26.00 (20.25 to 31.75)	23.00 (20.00 to 25.00)	0.053
TBIL (μmol/L), Median (IQR)	14.80 (10.15 to 20.03)	13.35 (11.68 to 14.15)	0.383
ALB (g/L), Median (IQR)	42.80 (41.15 to 44.95)	46.00 (43.43 to 47.45)	0.001
CREA (μmol/L), Median (IQR)	68.50 (59.25 to 79.50)	75.50 (67.25 to 81.50)	0.183
AFP (ng/mL), Median (IQR)	3.16 (2.26 to 4.53)	2.51 (1.67 to 3.45)	0.063
ANGPTL6 (pg/mL), Median (IQR)	582.10 (385.88 to 1309.13)	191.00 (99.80 to 519.50)	0.001
