# Supplementary material for: Screening for Lipid-Metabolism-Related Genes and Identifying the Diagnostic Potential of ANGPTL6 for HBV-Related Early-Stage Hepatocellular Carcinoma
Source: Biomolecules. 2022 Nov 17;12(11):1700. doi: 10.3390/biom12111700 (PMC9687352; doi:10.3390/biom12111700)
Supplement: Supplementary file 1 [file biomolecules-12-01700-s001.zip › Table S9.pdf]

Table S9. Characteristics of early primary HCC patients and CHB patients with normal serum AFP levels.

Characteristics	HCC Patients	CHB patients	*p*-Value
Case	32	30	-
Age (years), Mean ± SD	61.22 ± 9.69	59.03 ± 10.57	0.399
Male, *n* (%)	29 (90.63)	26 (86.67)	0.623
Child-Push Class			0.588
A, *n* (%)	30 (93.75)	27 (90.00)	
B, *n* (%)	2 (6.25)	3 (10.00)	
Cirrhosis, *n* (%)	23 (71.88)	19 (63.33)	0.472
HBsAg Positive, *n* (%)	32 (100)	30 (100)	-
HCV-Ab Positive, *n* (%)	0 (0)	0 (0)	-
HEV-IgM Positive, *n* (%)	0 (0)	0 (0)	-
BMI (kg/m^2), Median (IQR)	24.25 (23.73 to 25.28)	24.00 (21.93 to 24.93)	0.078
ALT (U/L), Median (IQR)	23.00 (14.25 to 31.50)	21.50 (16.50 to 35.00)	0.855
AST (U/L), Median (IQR)	26.00 (20.25 to 31.75)	24.50 (14.75 to 32.50)	0.209
TBIL (μmol/L), Median (IQR)	14.80 (10.15 to 20.03)	14.85 (10 40 to 23.43)	0.398
ALB (g/L), Median (IQR)	42.80 (41.15 to 44.95)	41.90 (36.03 to 45.03)	0.324
PT (sec), Median (IQR)	11.55 (11.23 to 11.90)	11.30 (10.10 to 12.55)	0.313
CREA (μmol/L), Median (IQR)	68.50 (59.25 to 79.50)	66.00 (63.50 to 74.50)	0.662
AFP (ng/mL), Median (IQR)	3.16 (2.26 to 4.53)	2.46 (1.74 to 3.58)	0.053
ANGPTL6 (pg/mL), Median (IQR)	582.10 (385.88 to 1309.13)	421.78 (369.04 to 496.56)	0.013

The CHB patients had a total of 11 CHB patients without cirrhosis and 19 HBV-related liver cirrhosis patients. *Abbreviation*: CHB, chronic hepatitis B.
