# Supplementary figures and images for: Screening for Lipid-Metabolism-Related Genes and Identifying the Diagnostic Potential of ANGPTL6 for HBV-Related Early-Stage Hepatocellular Carcinoma
Source: Biomolecules. 2022 Nov 17;12(11):1700. doi: 10.3390/biom12111700 (PMC9687352; doi:10.3390/biom12111700)

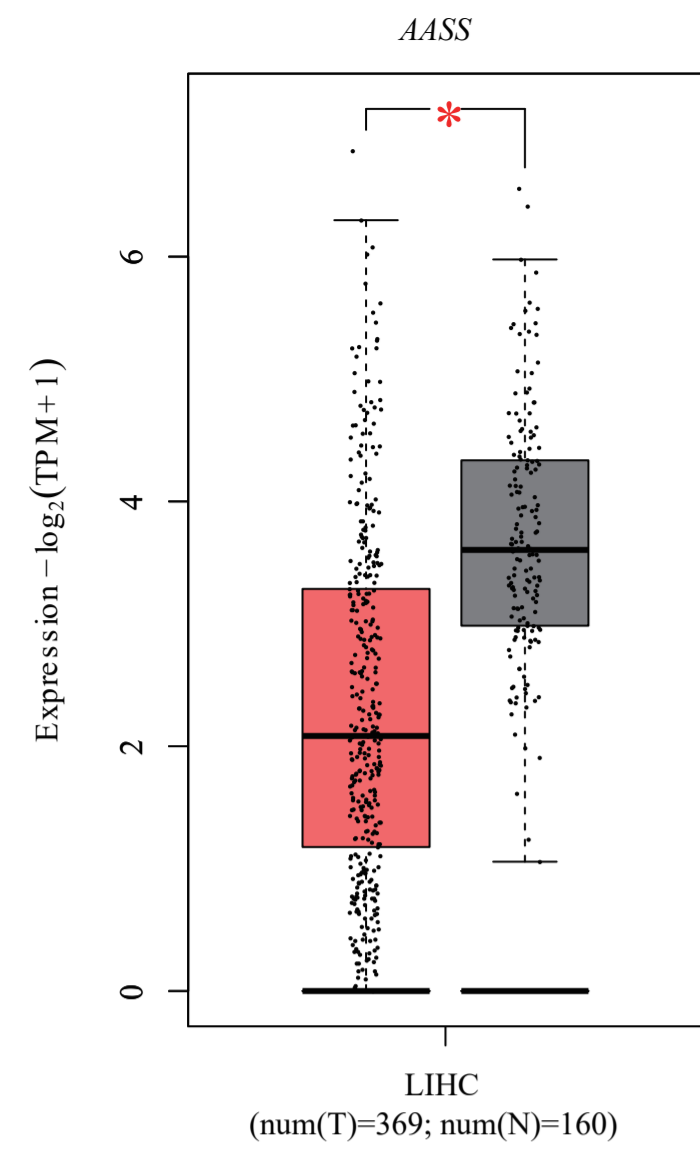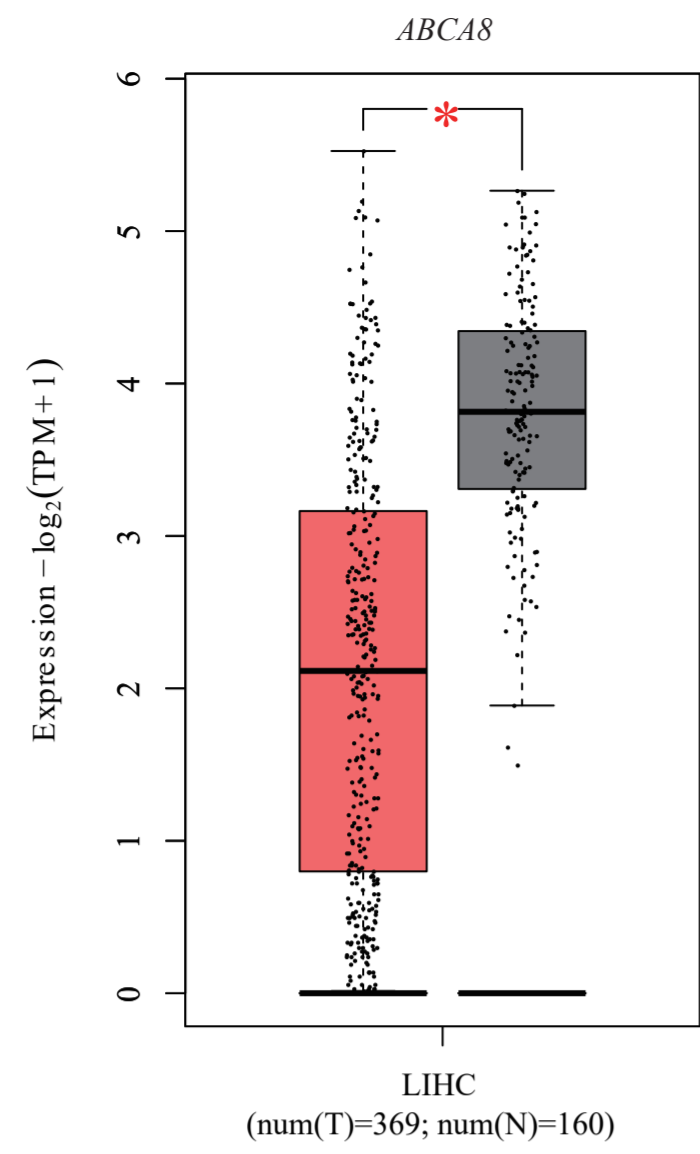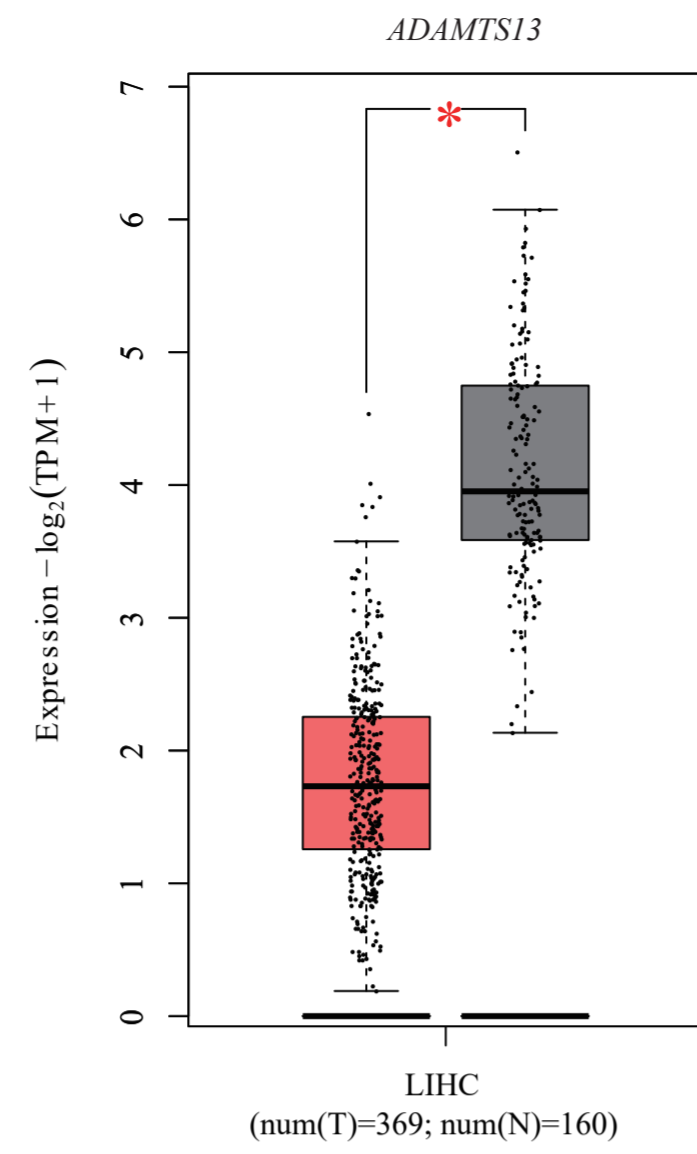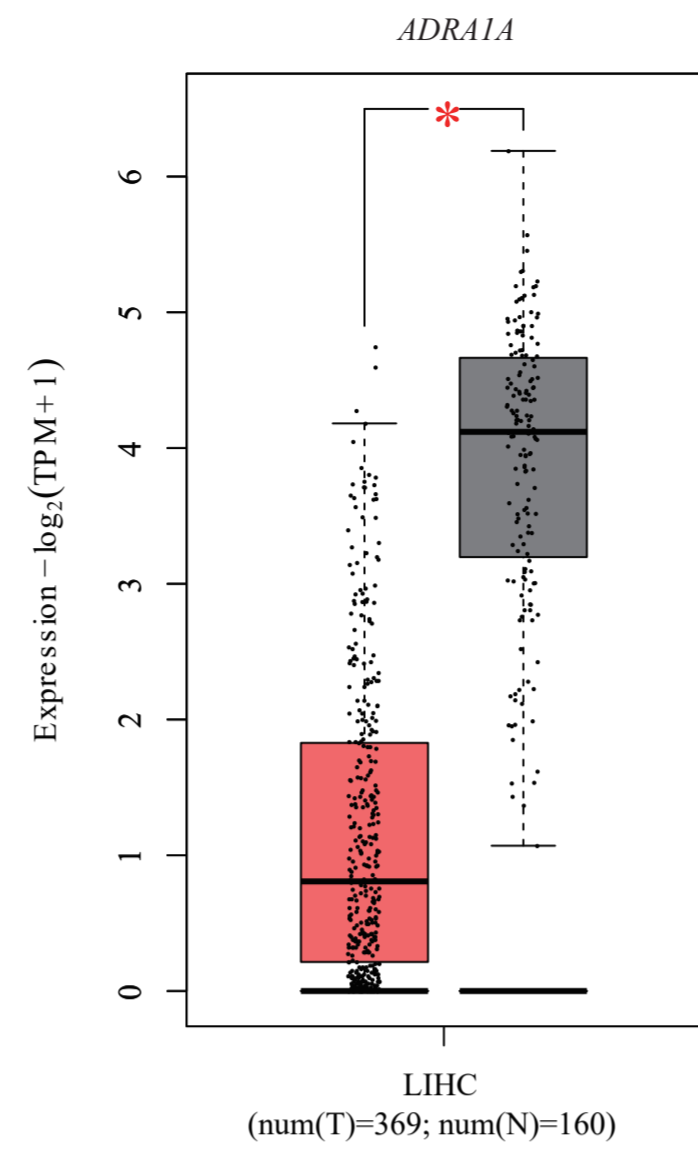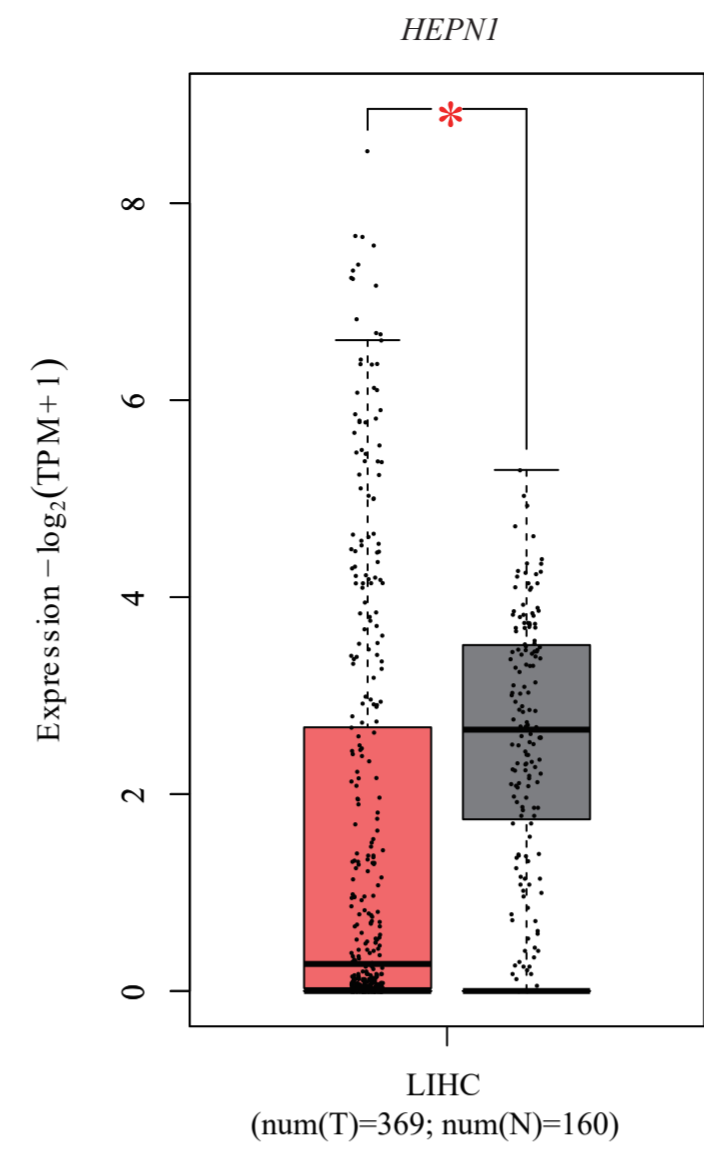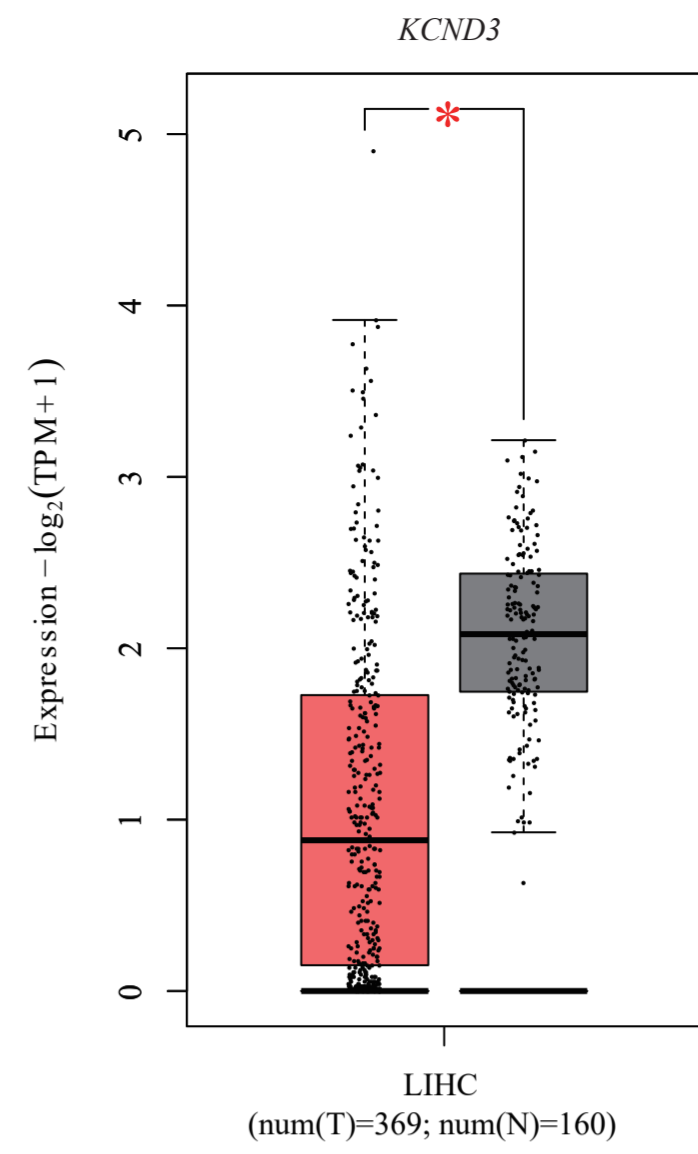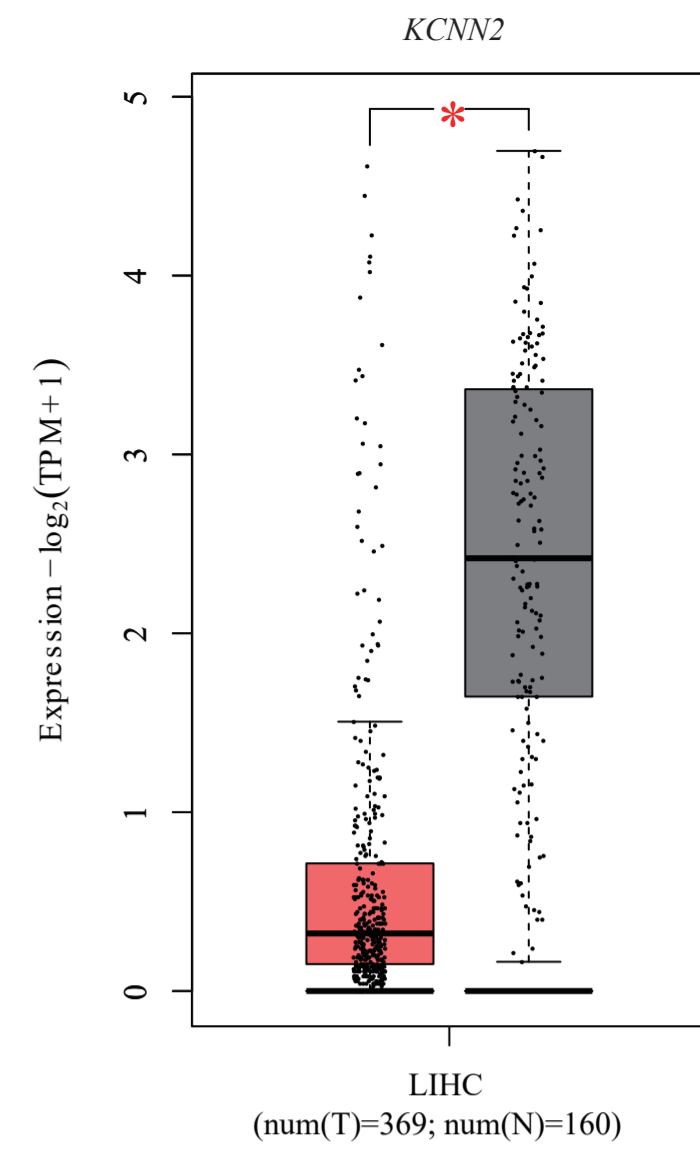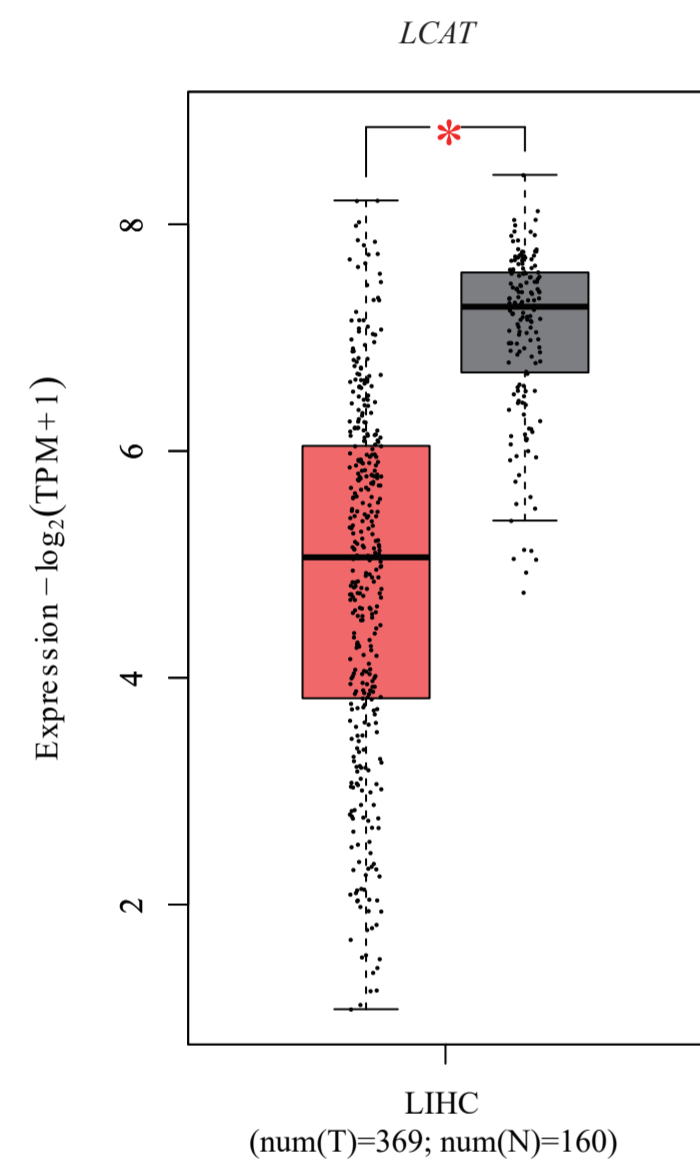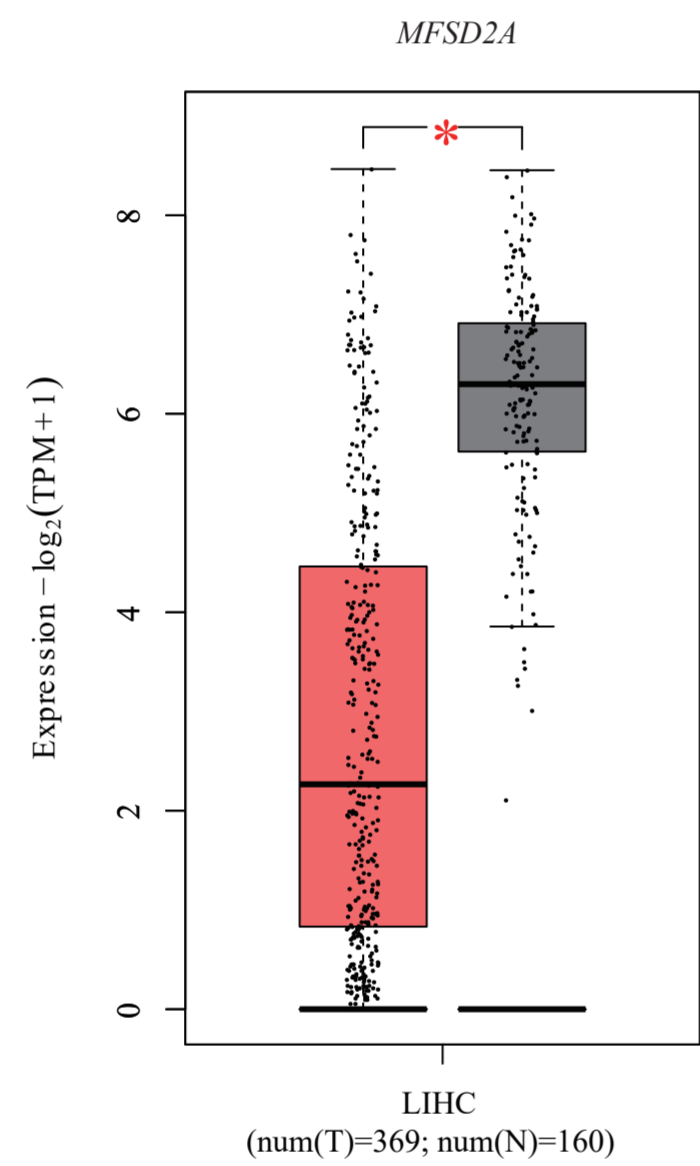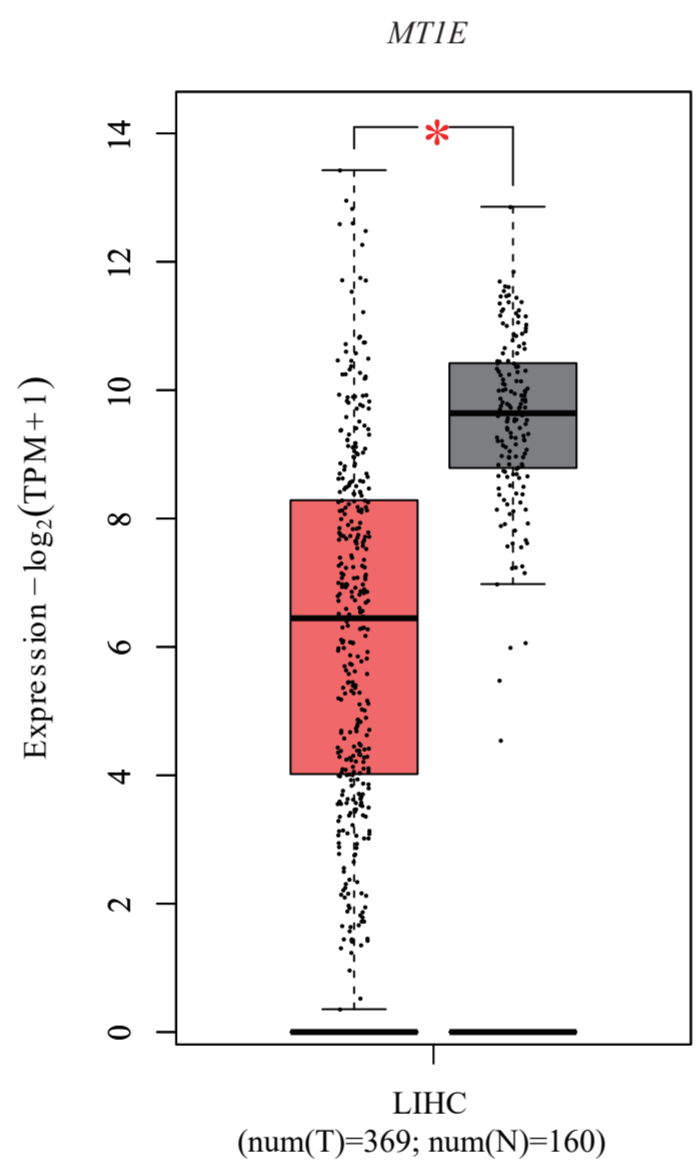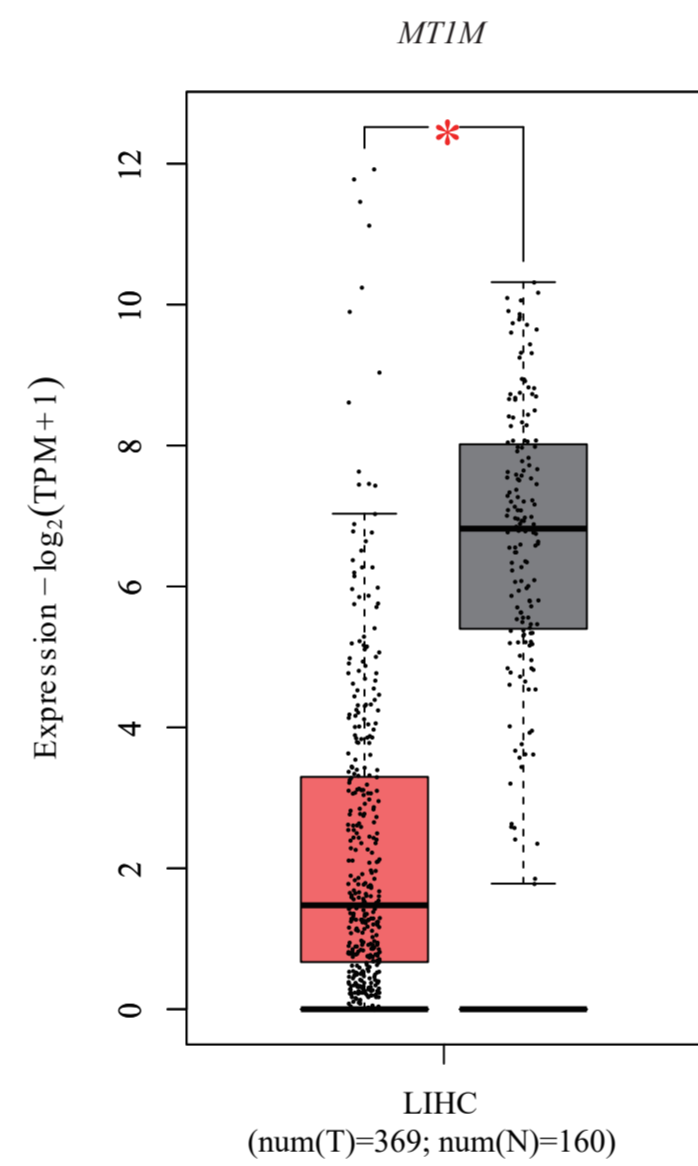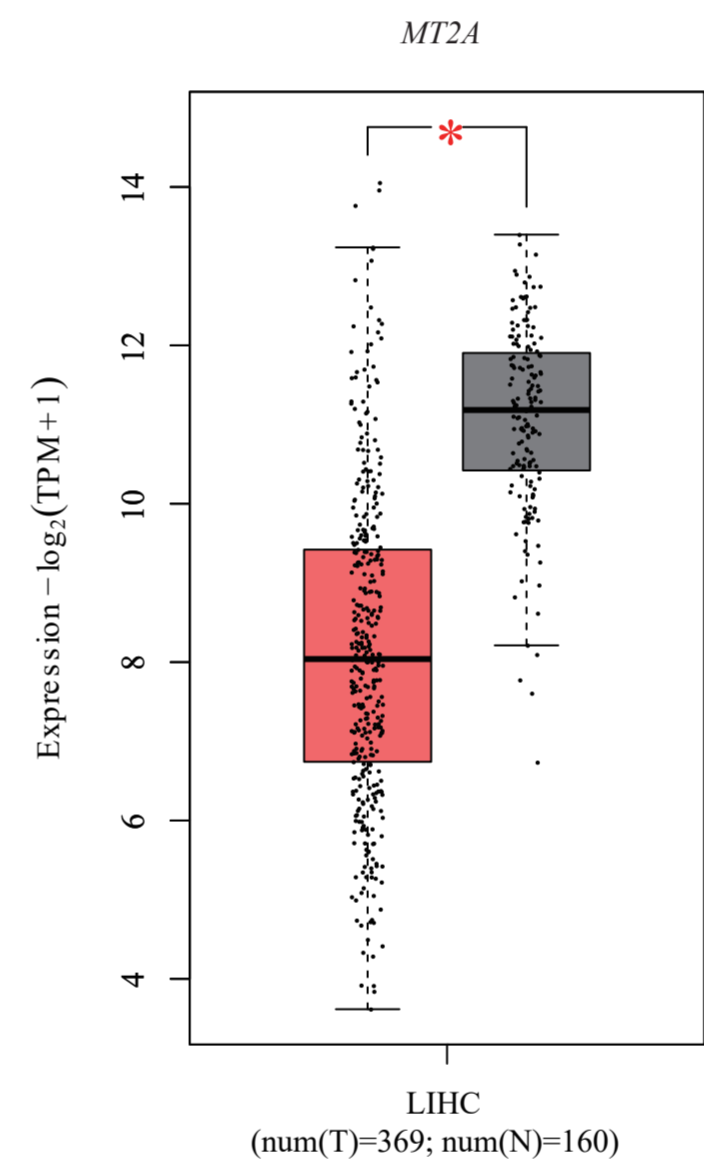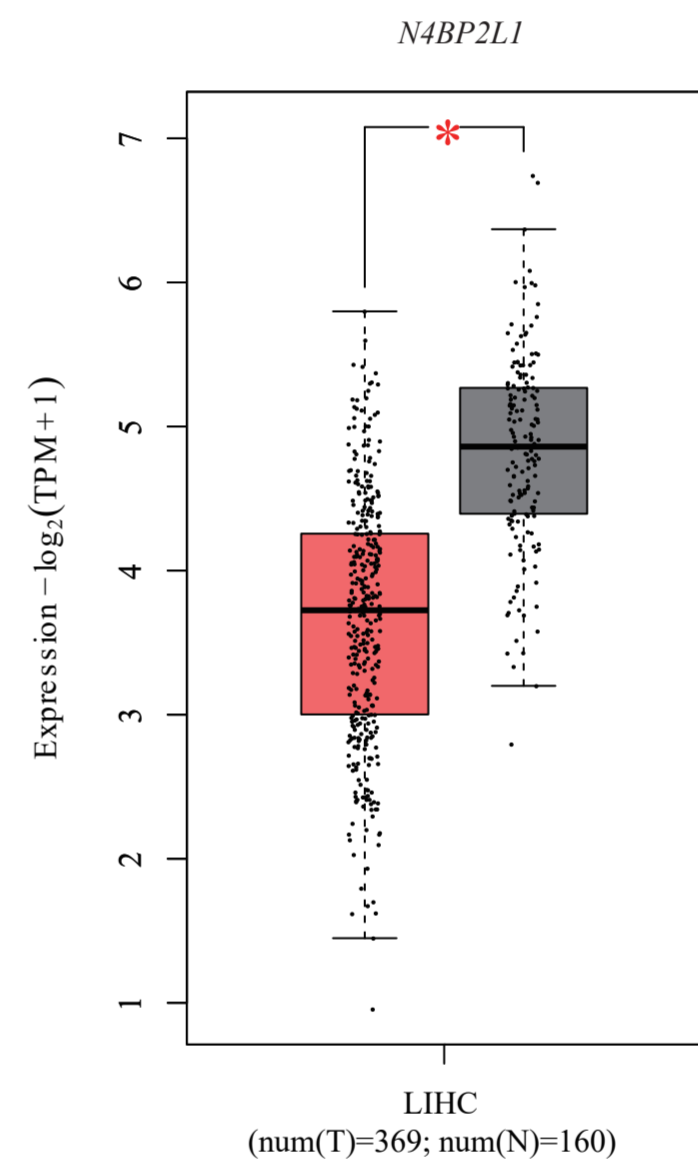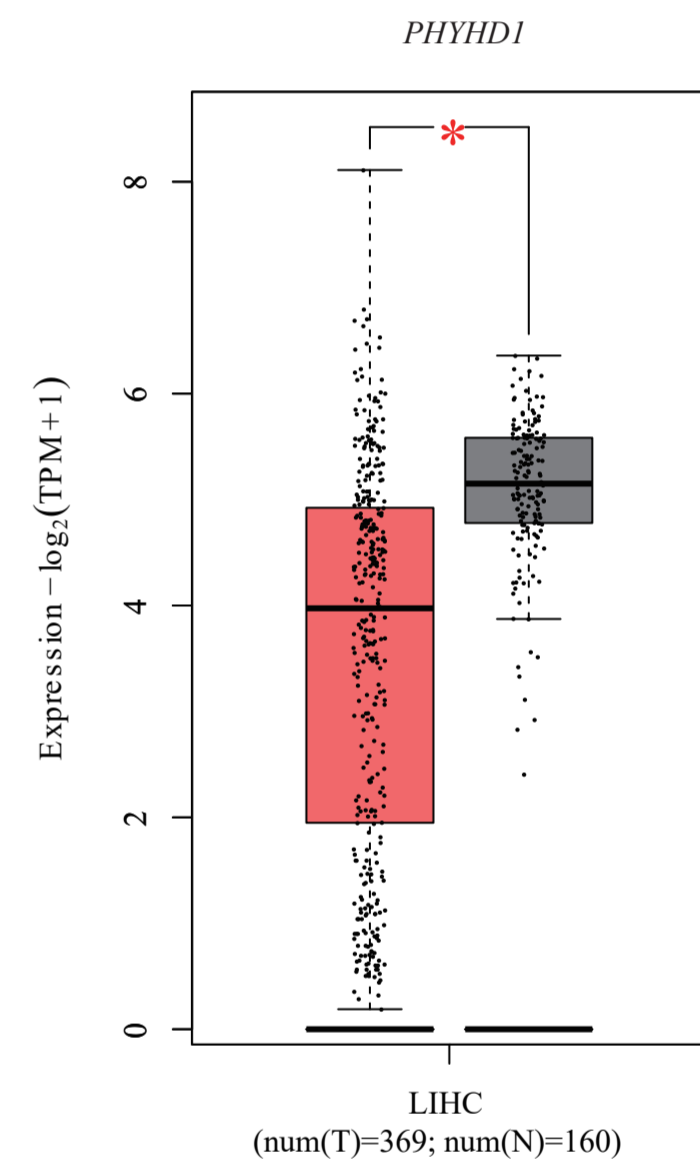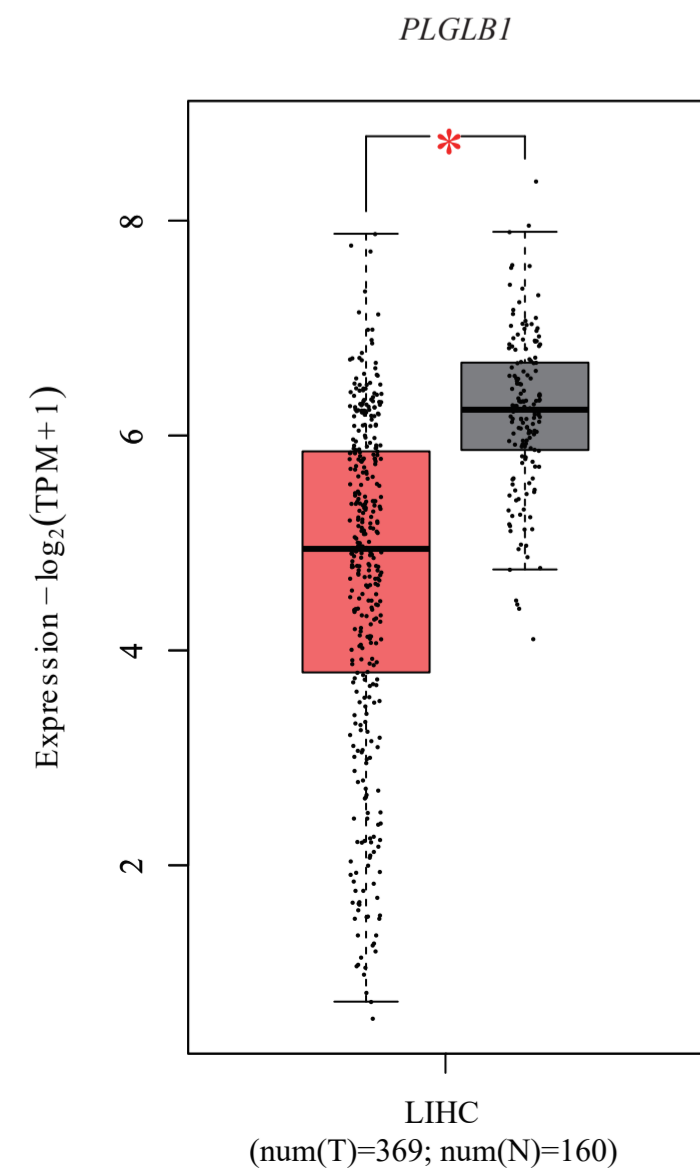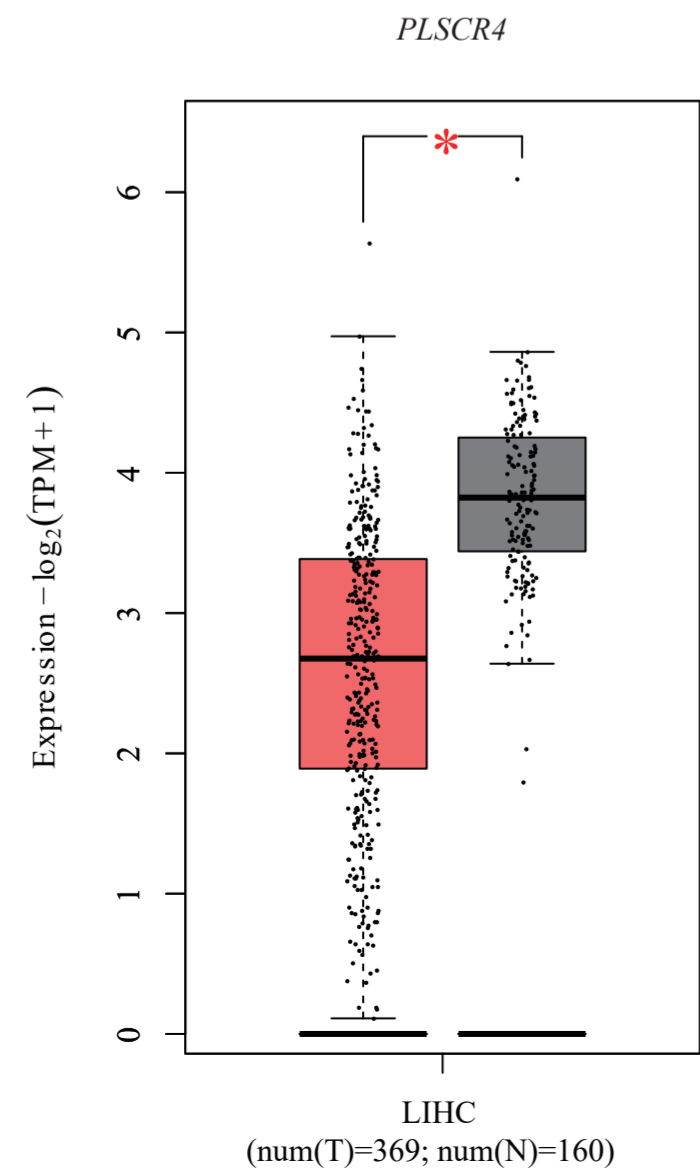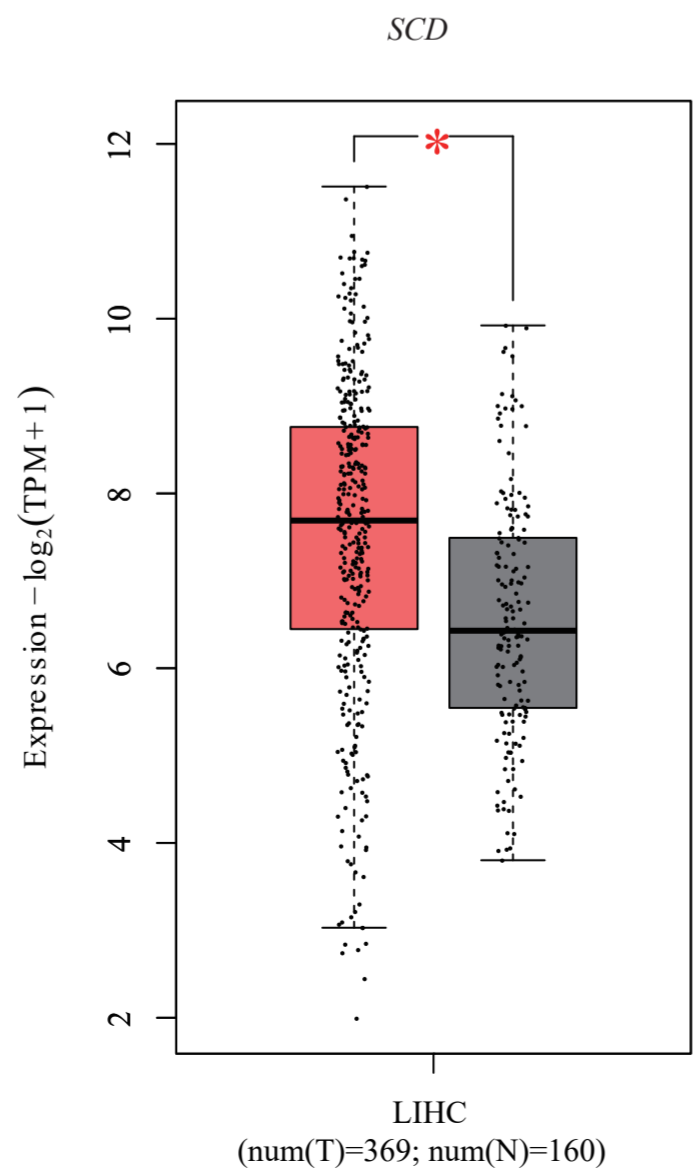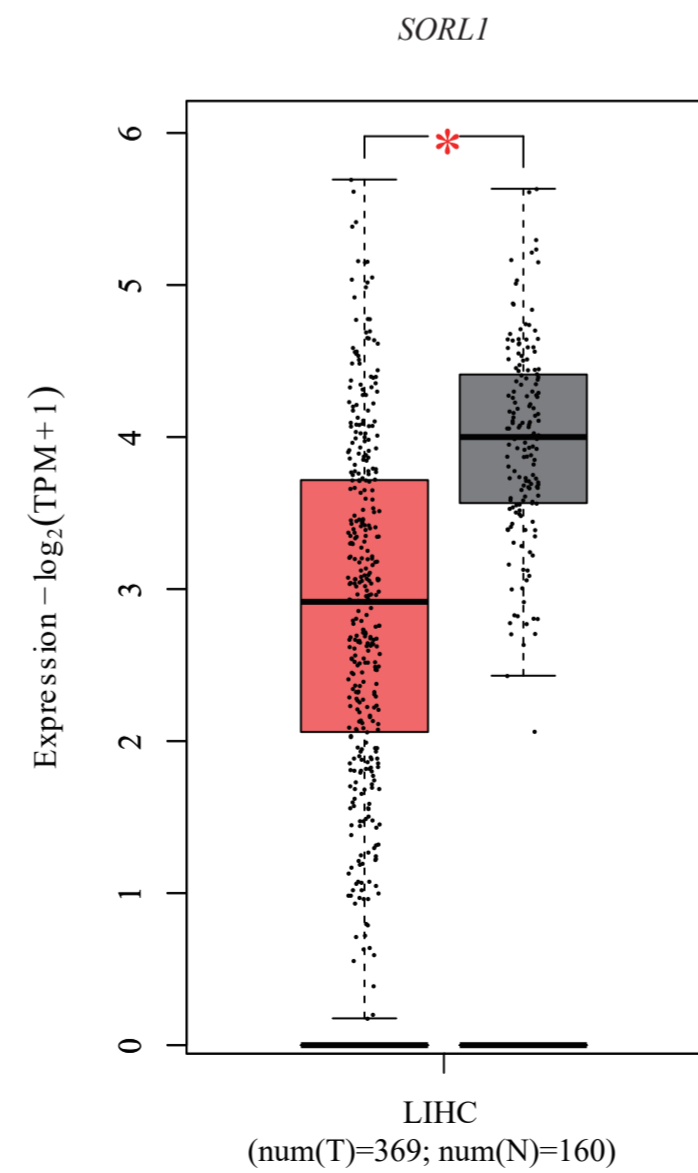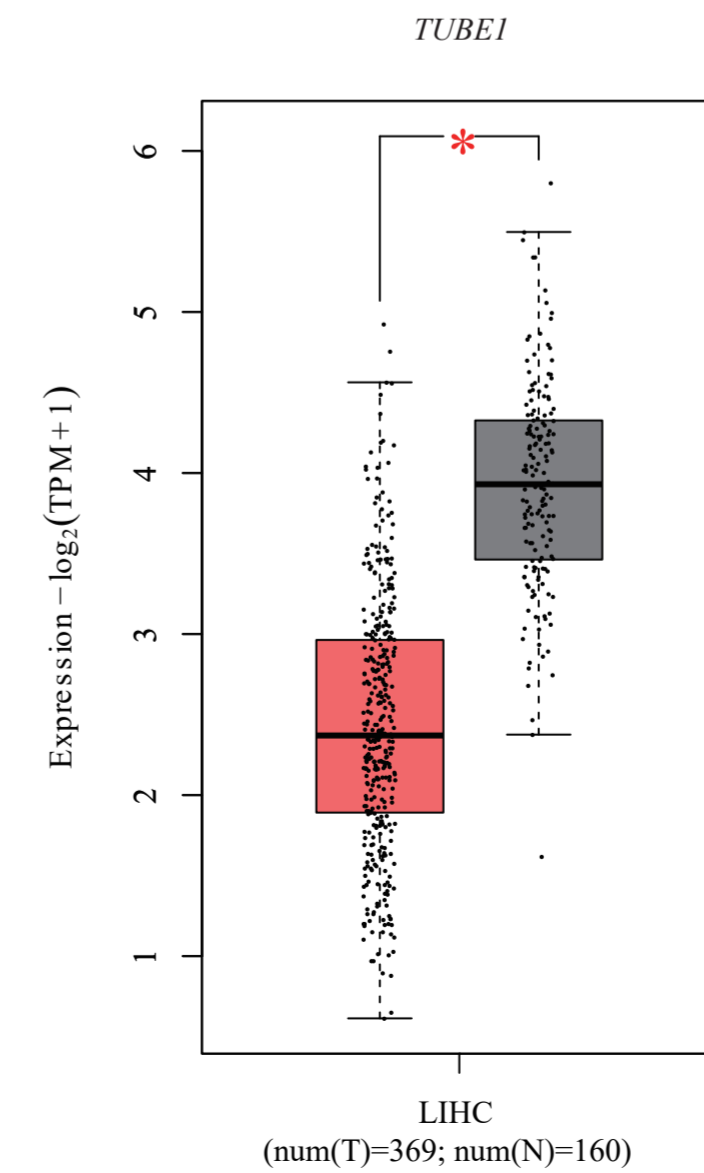

Supplement: Supplementary file 1 [file biomolecules-12-01700-s001.zip › Figure S1.pdf]

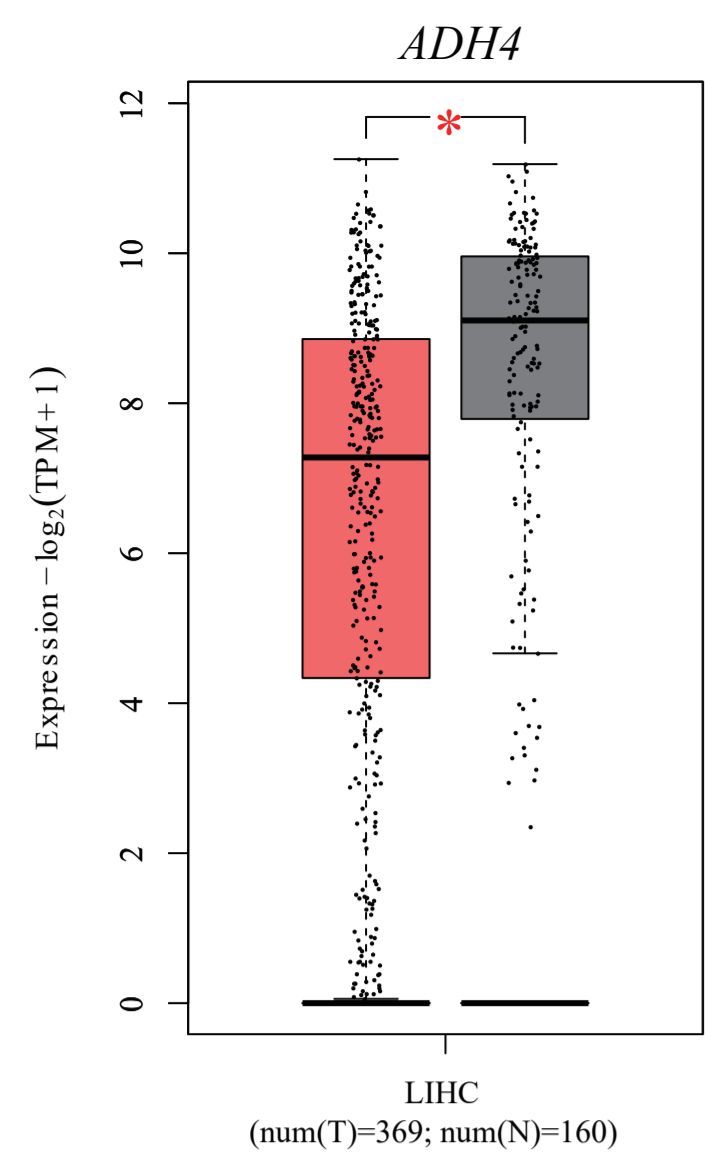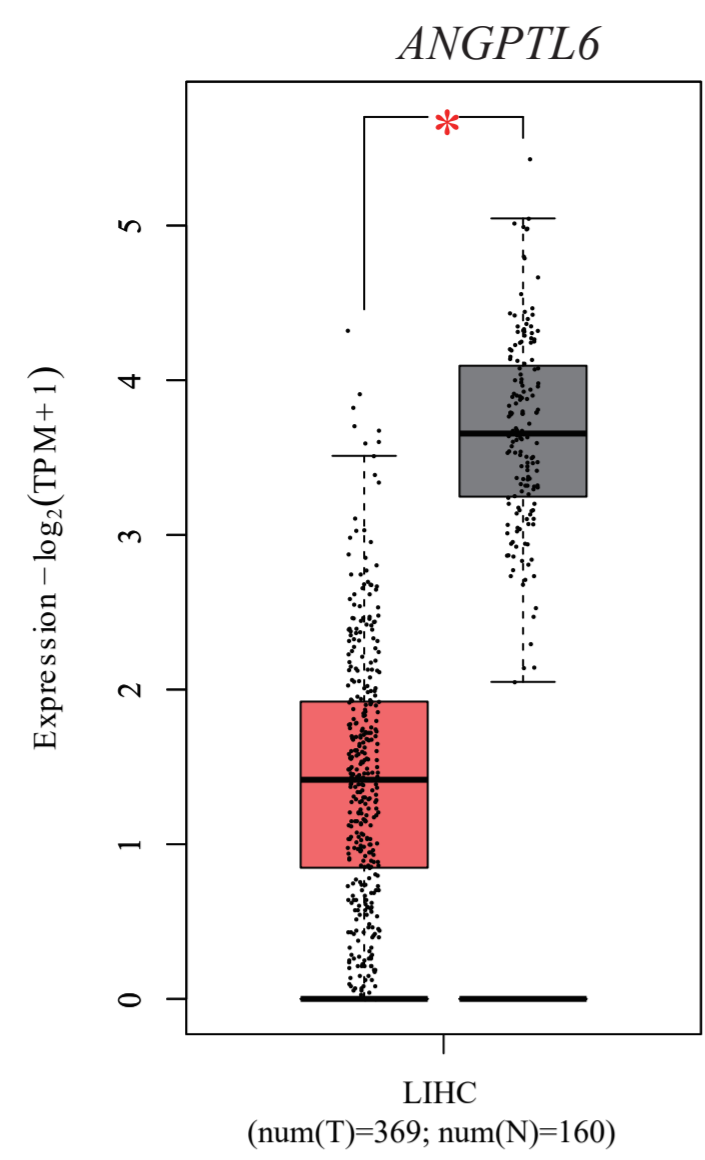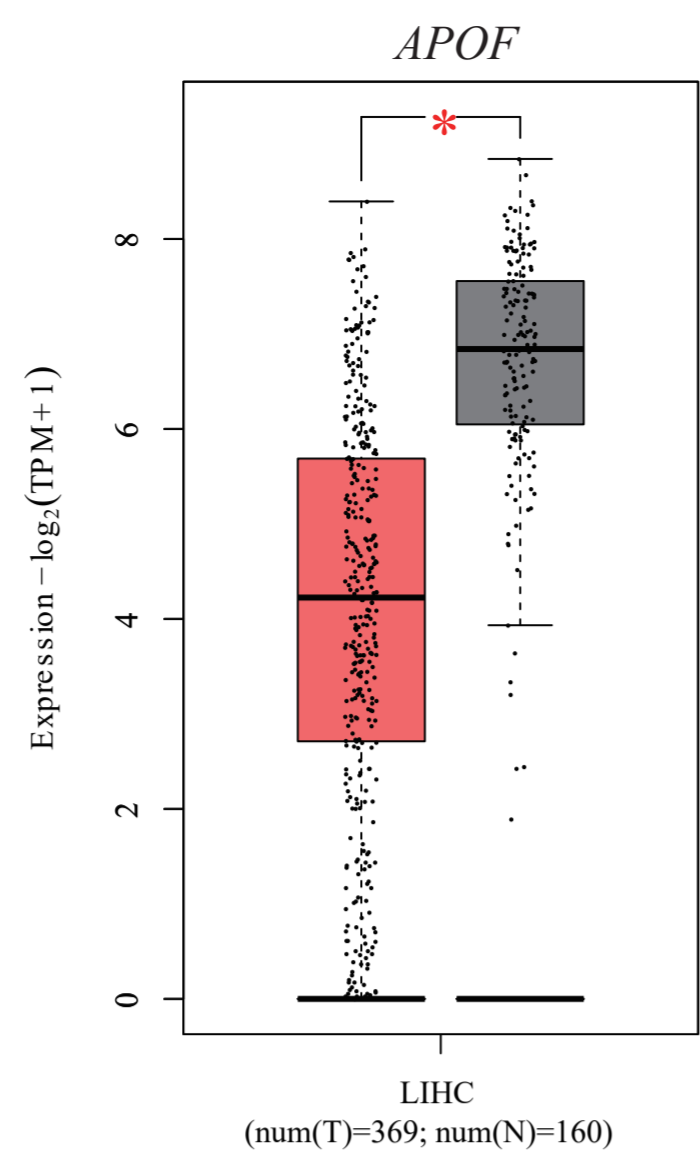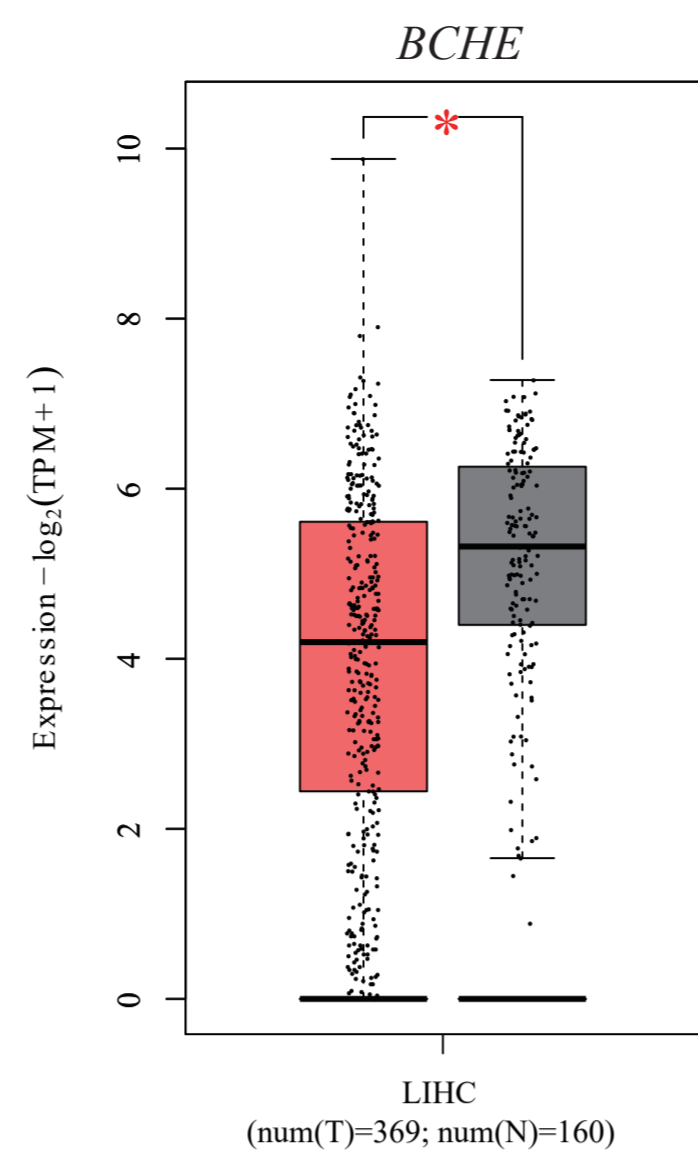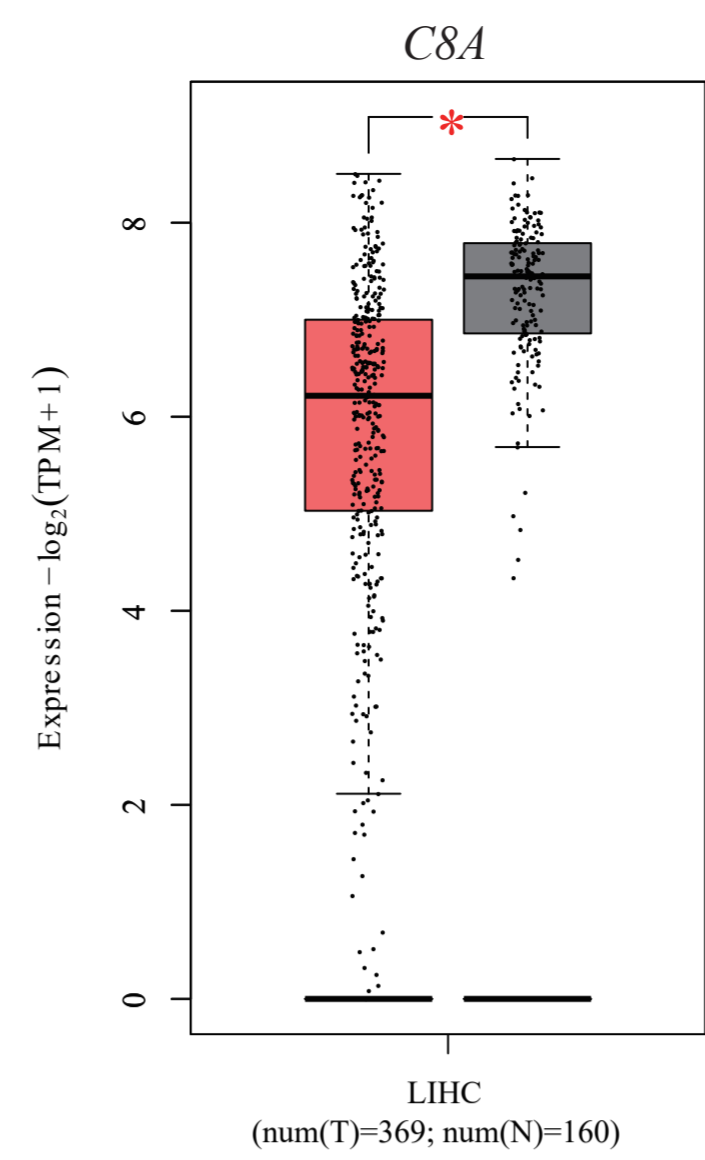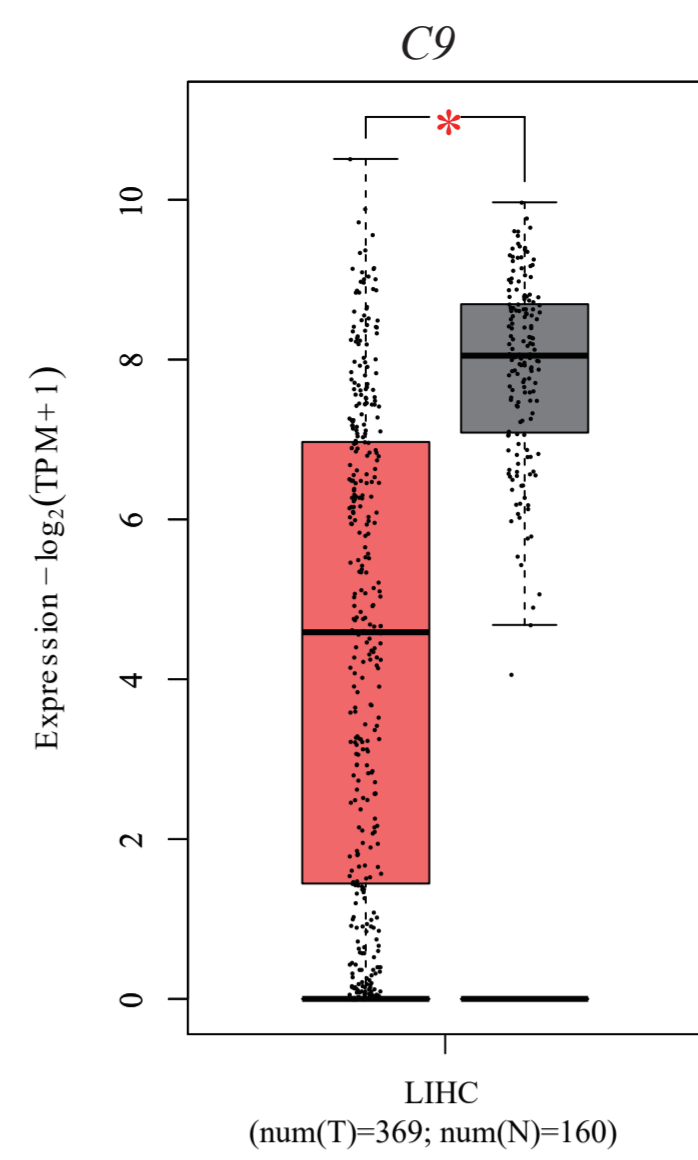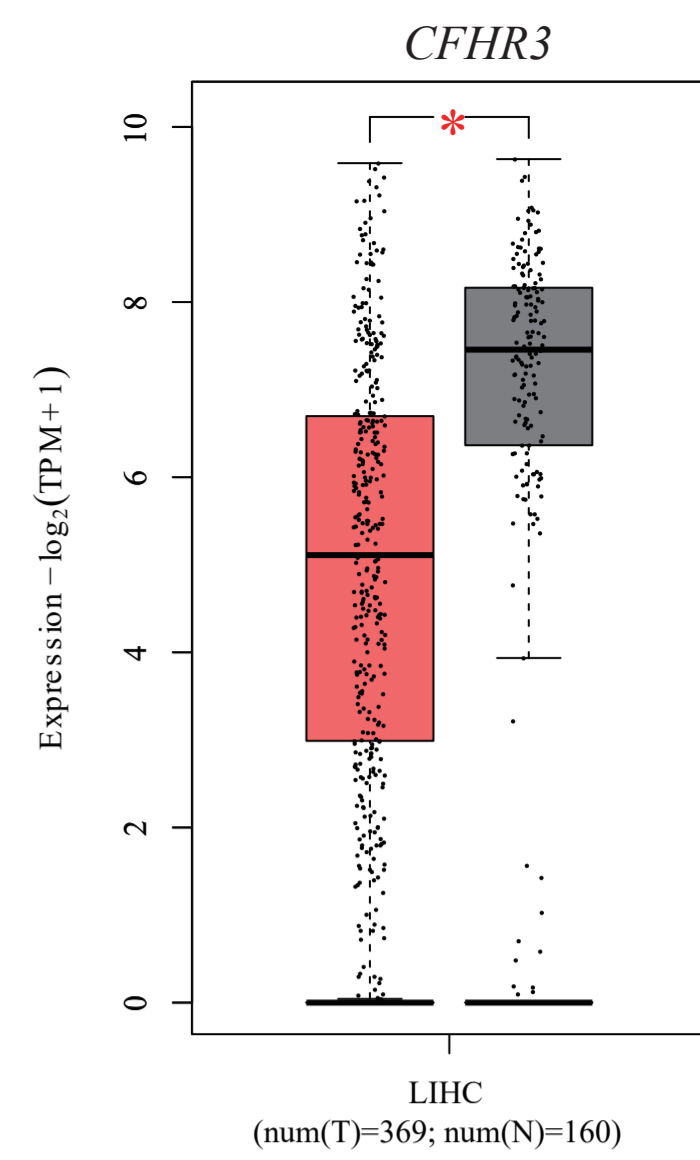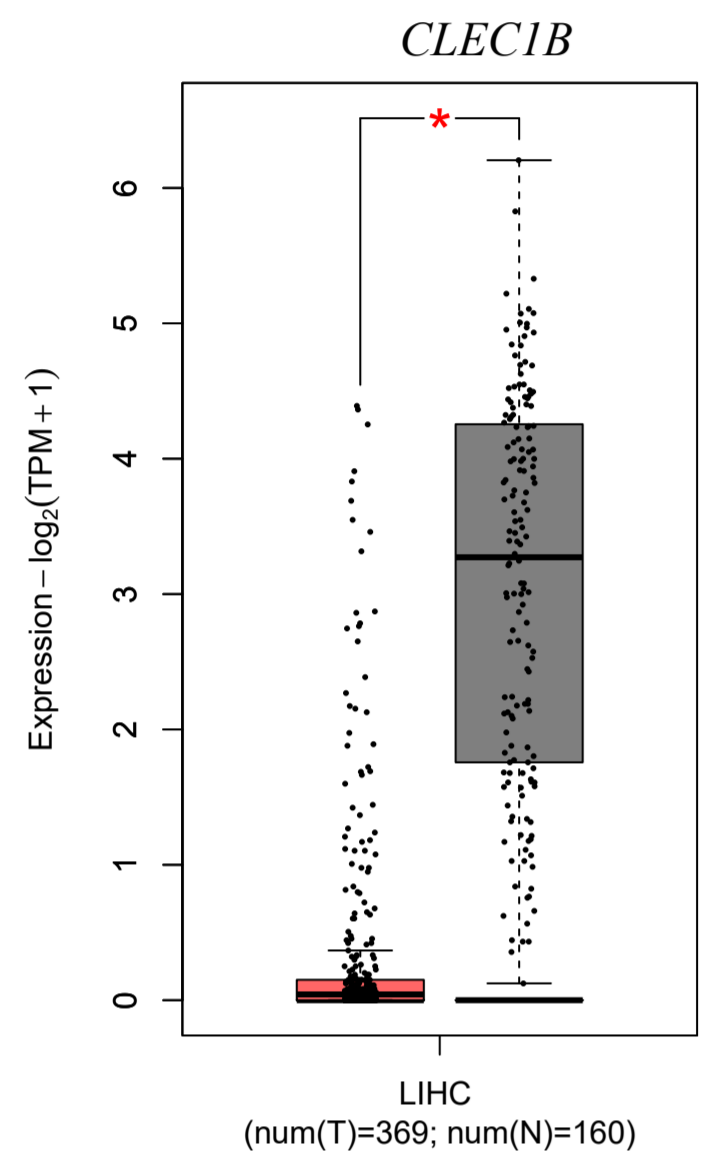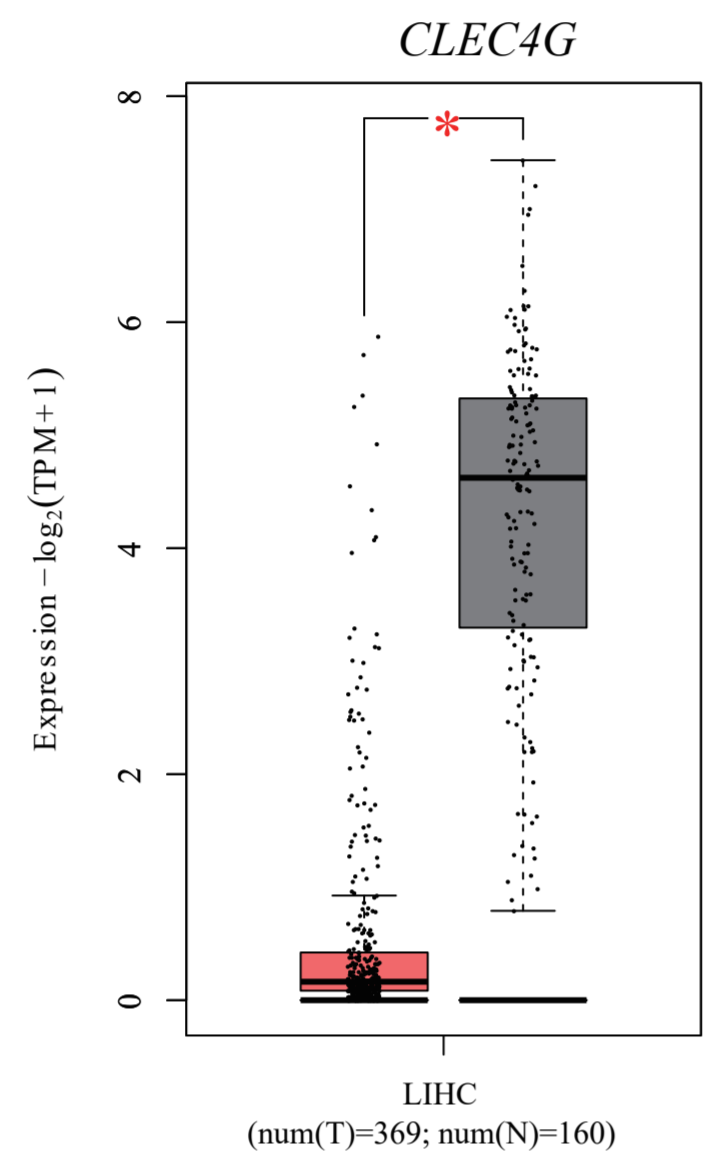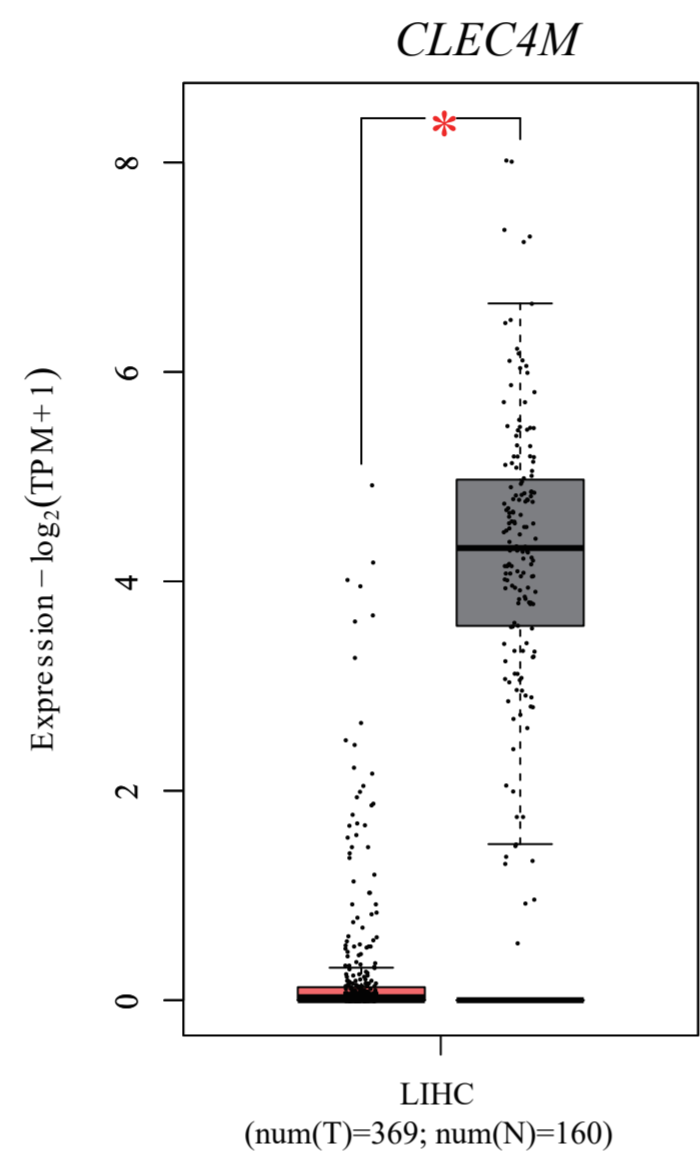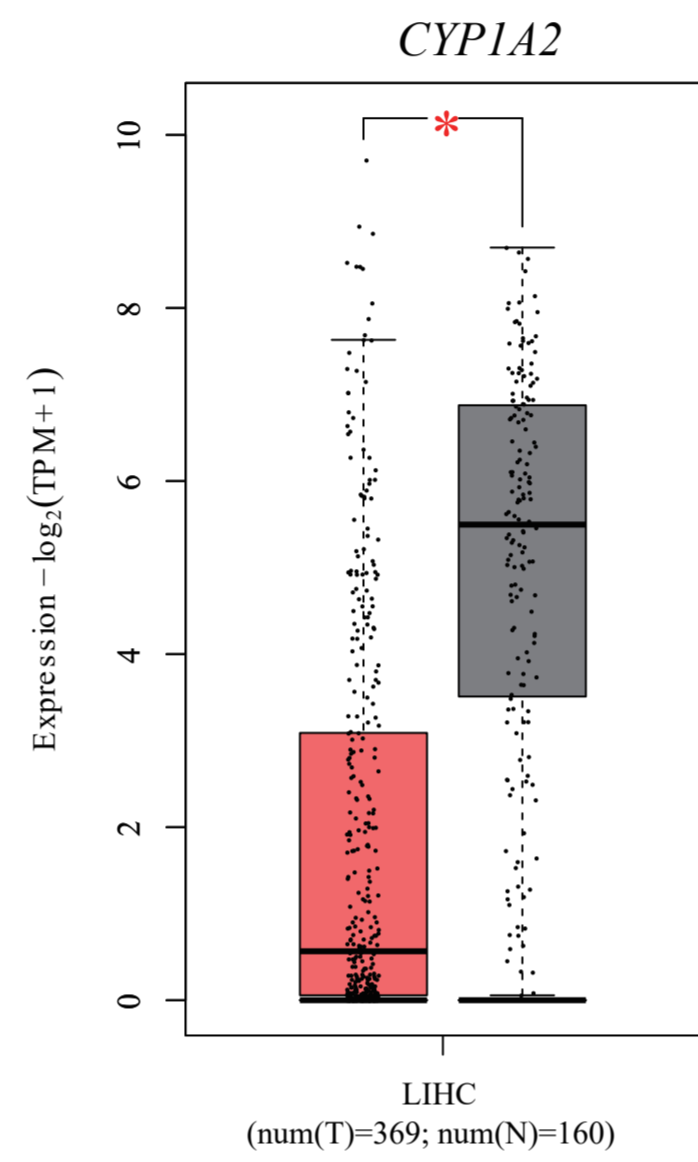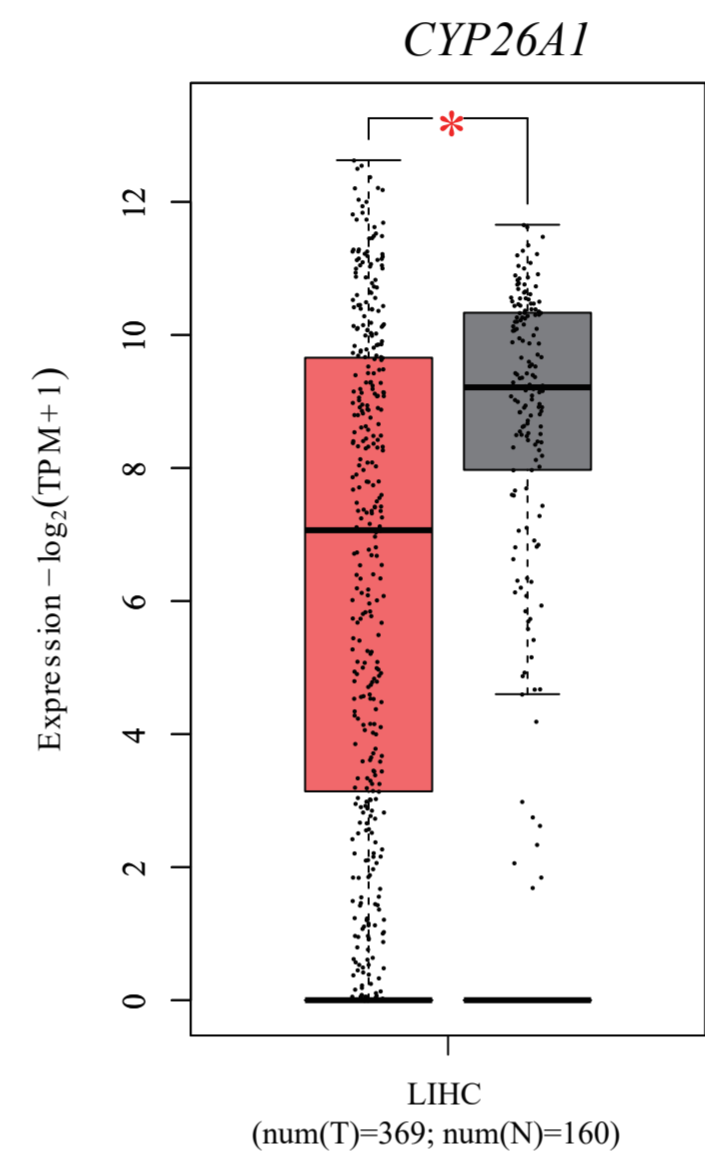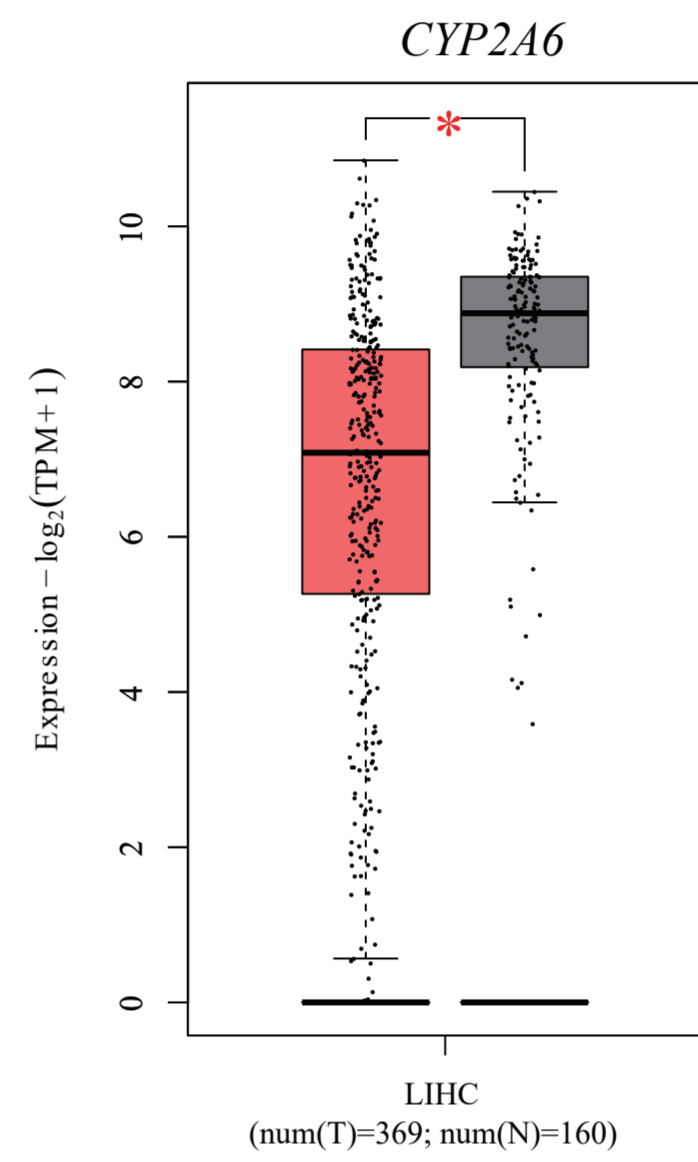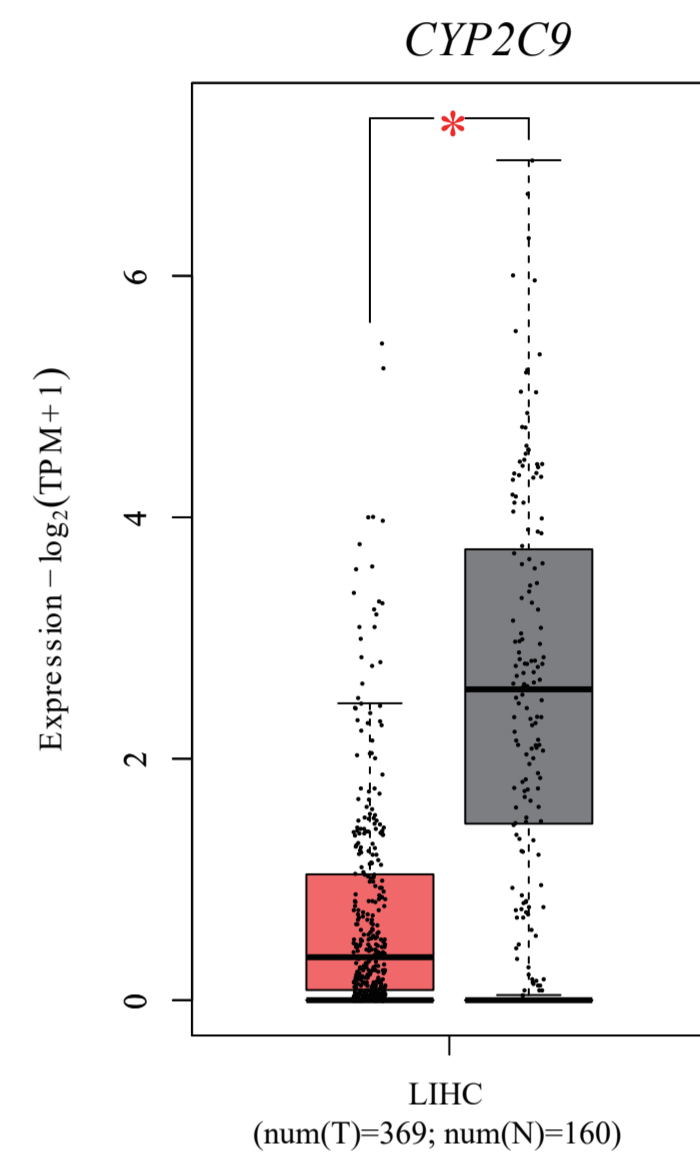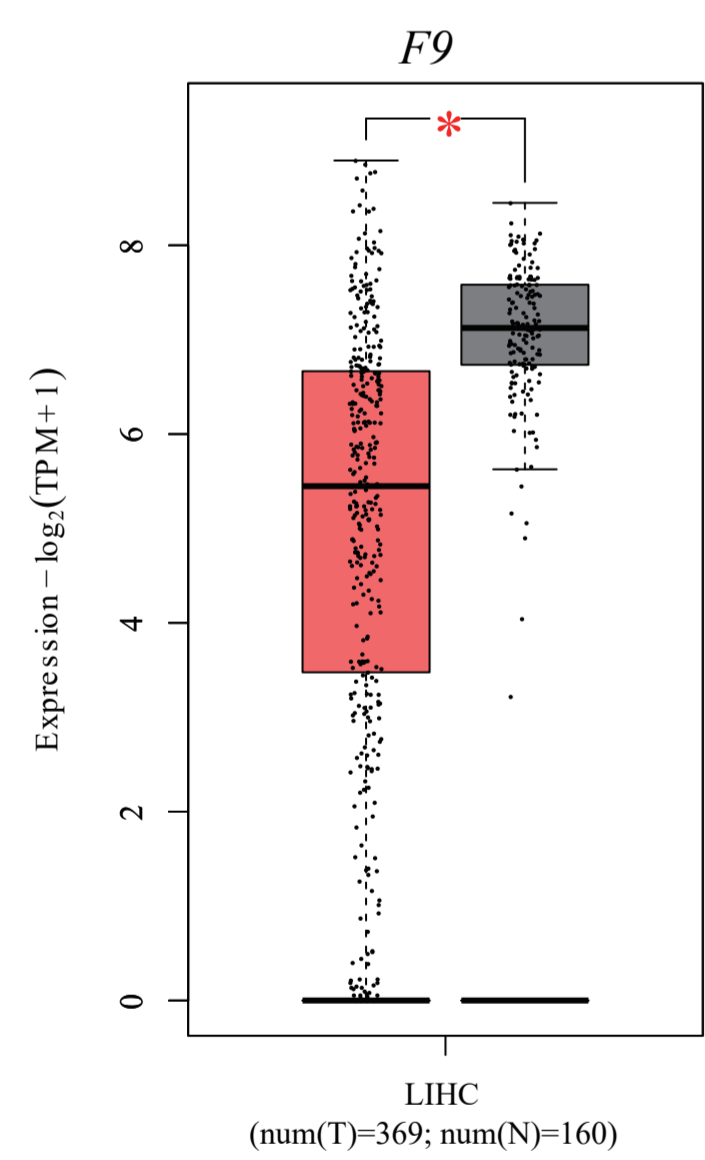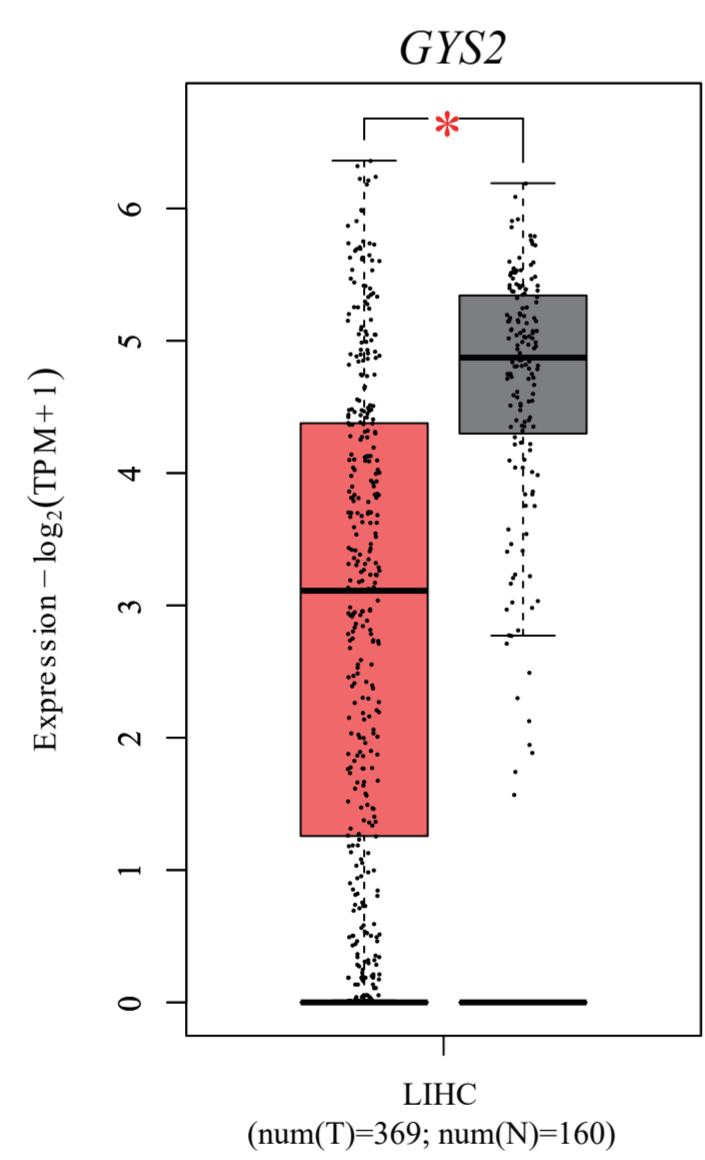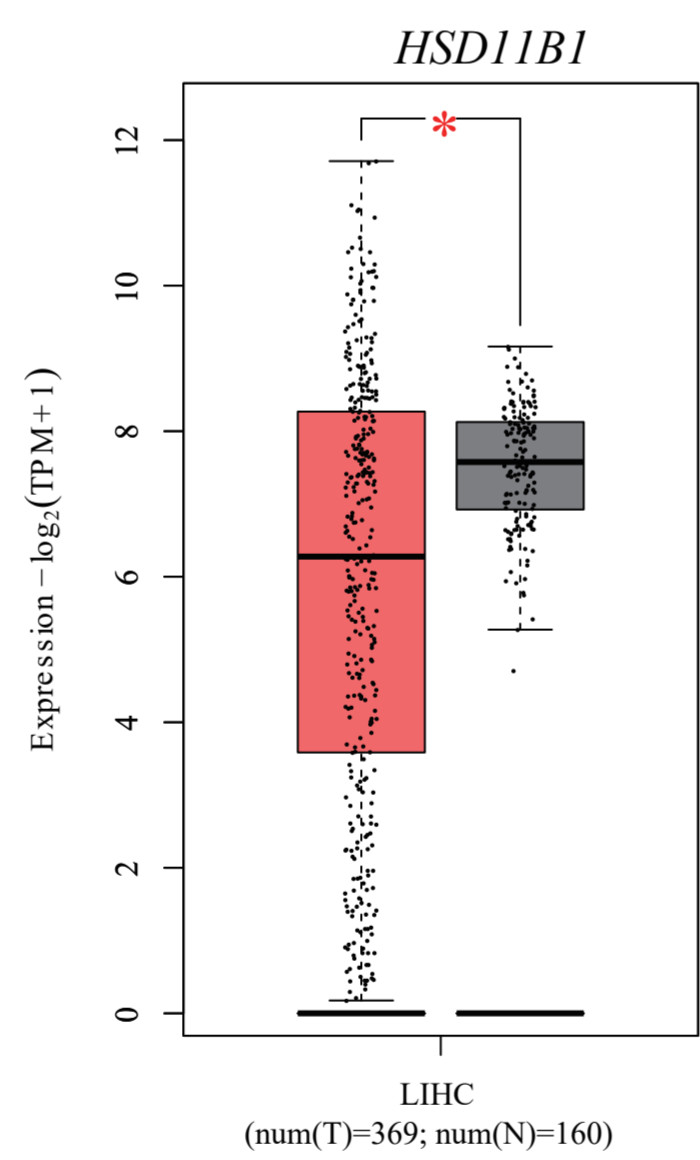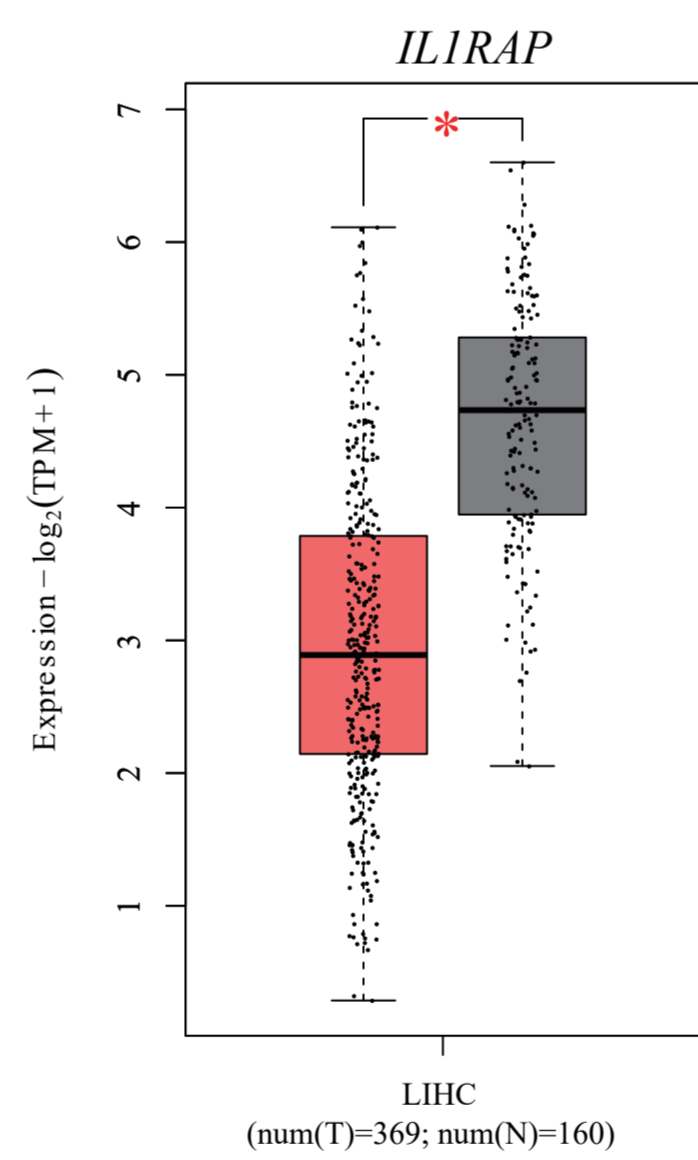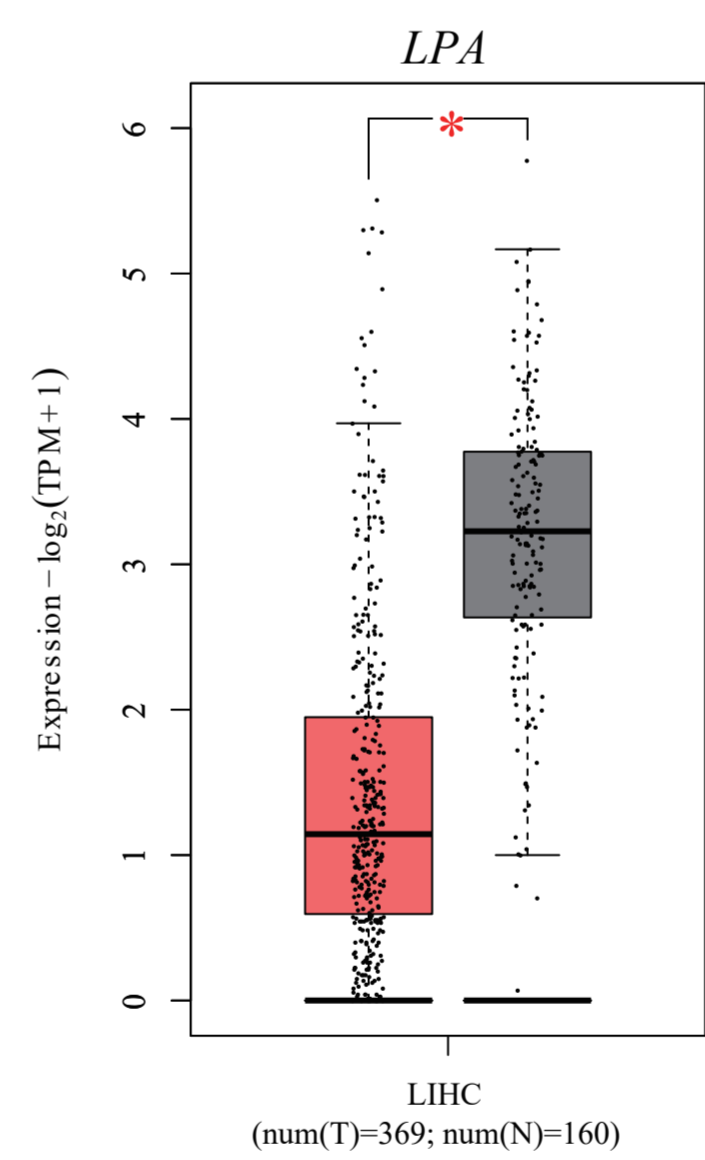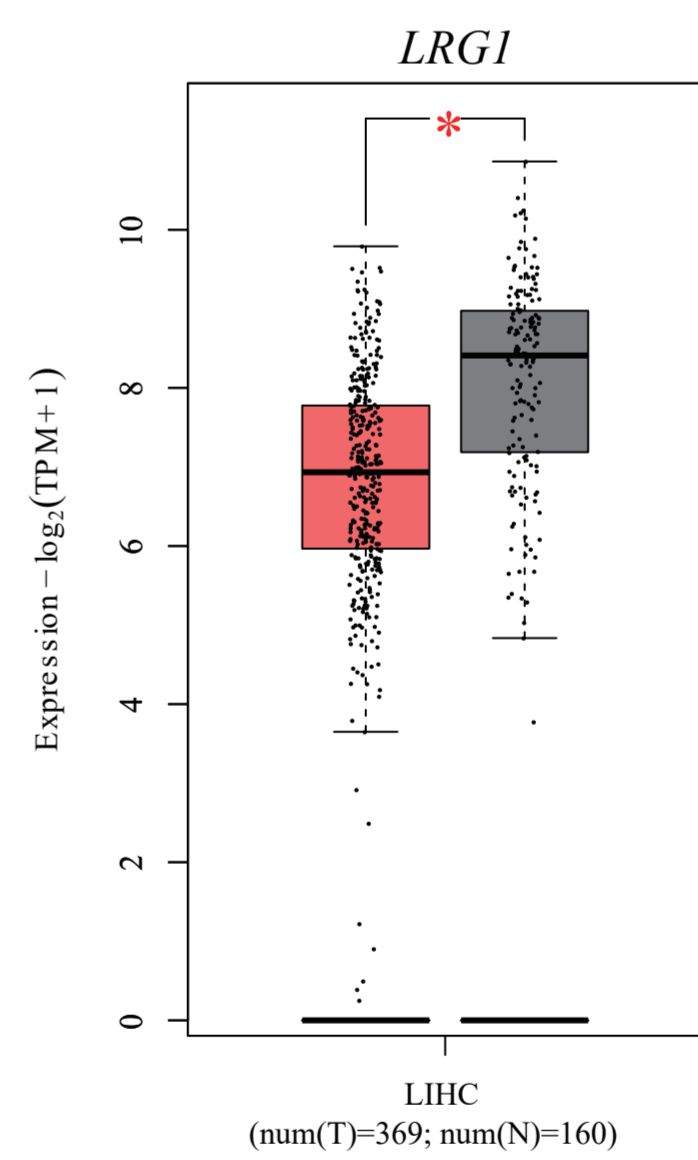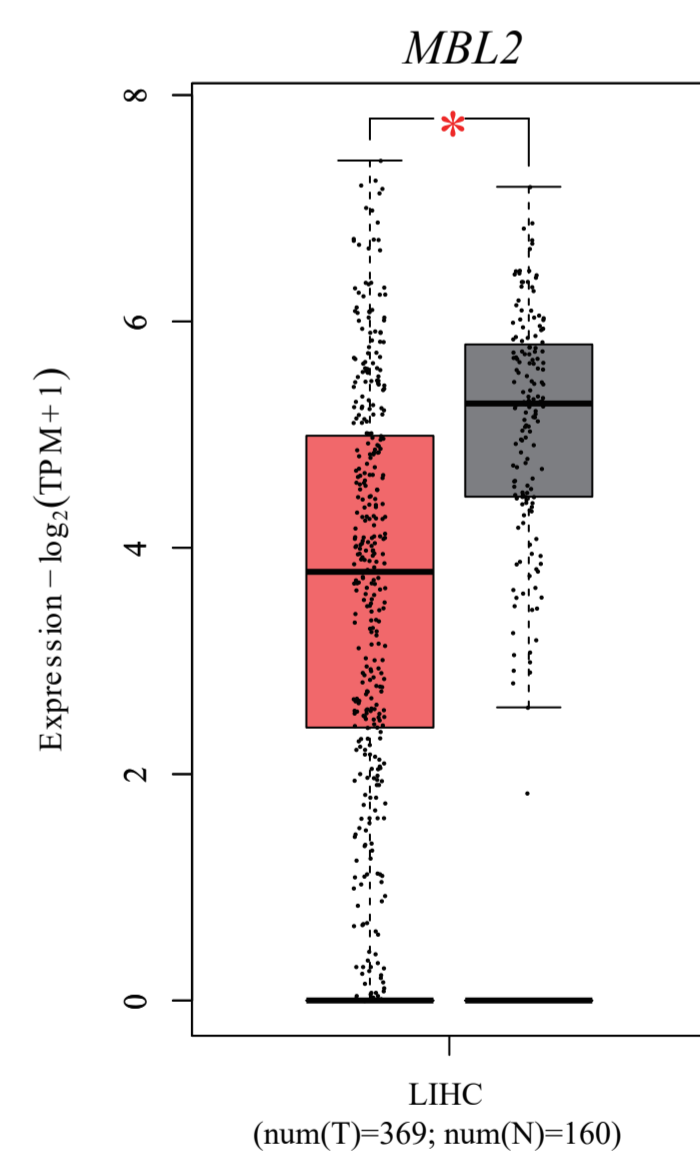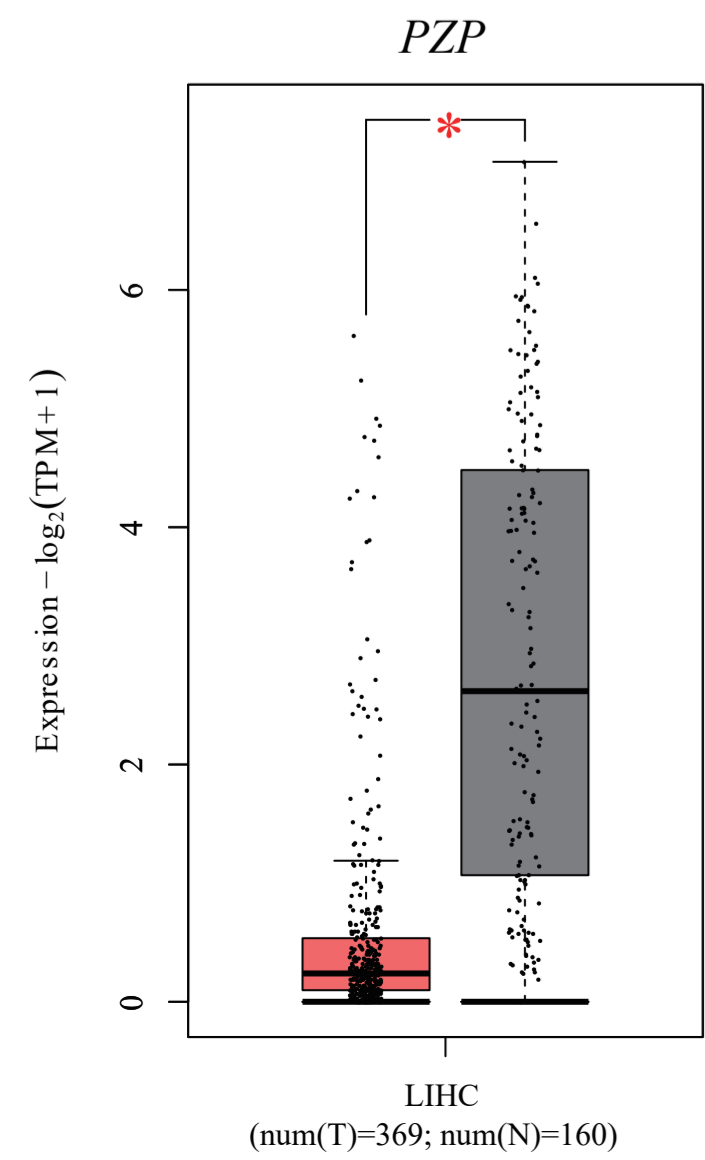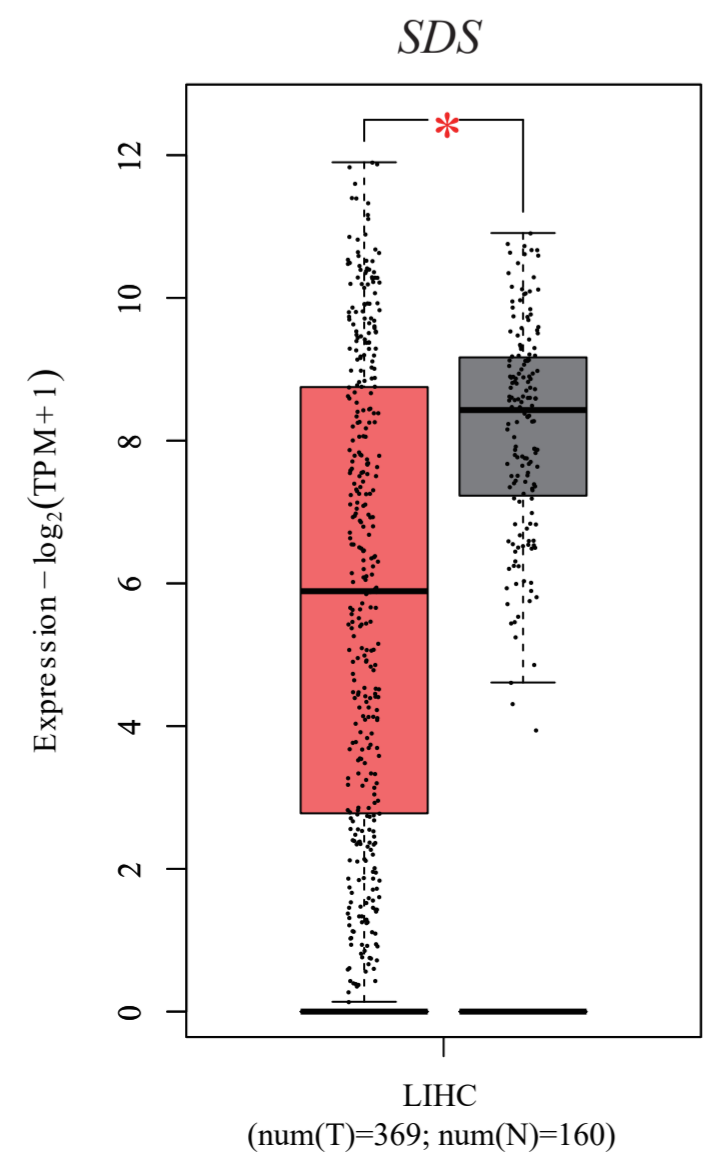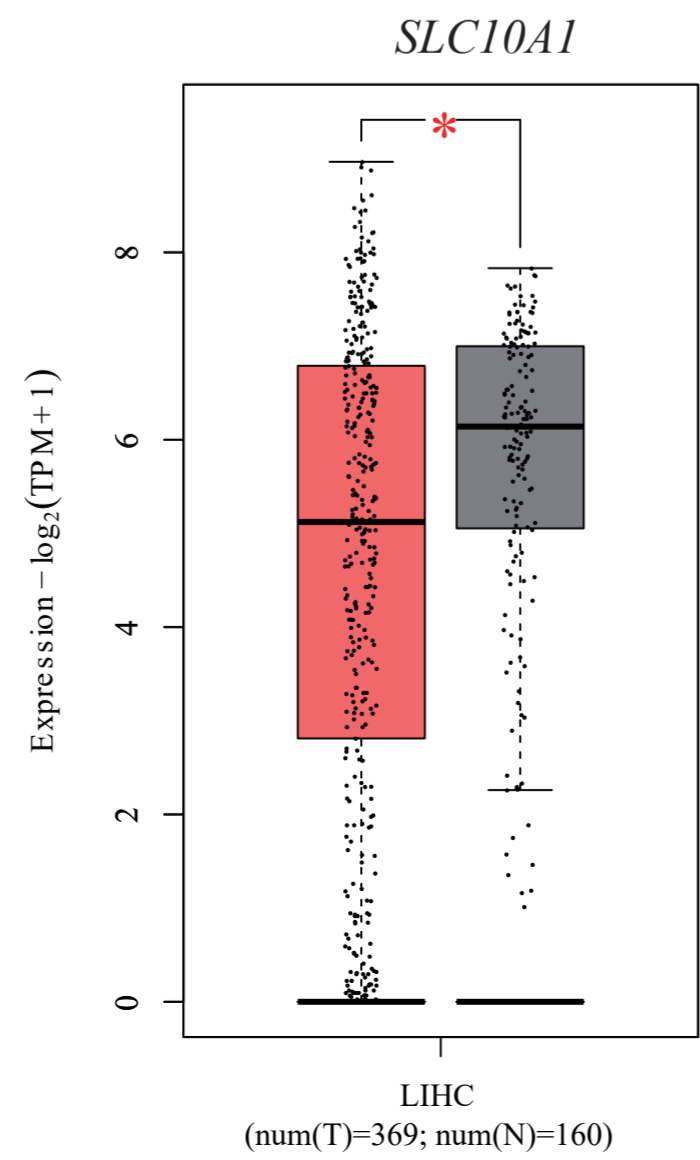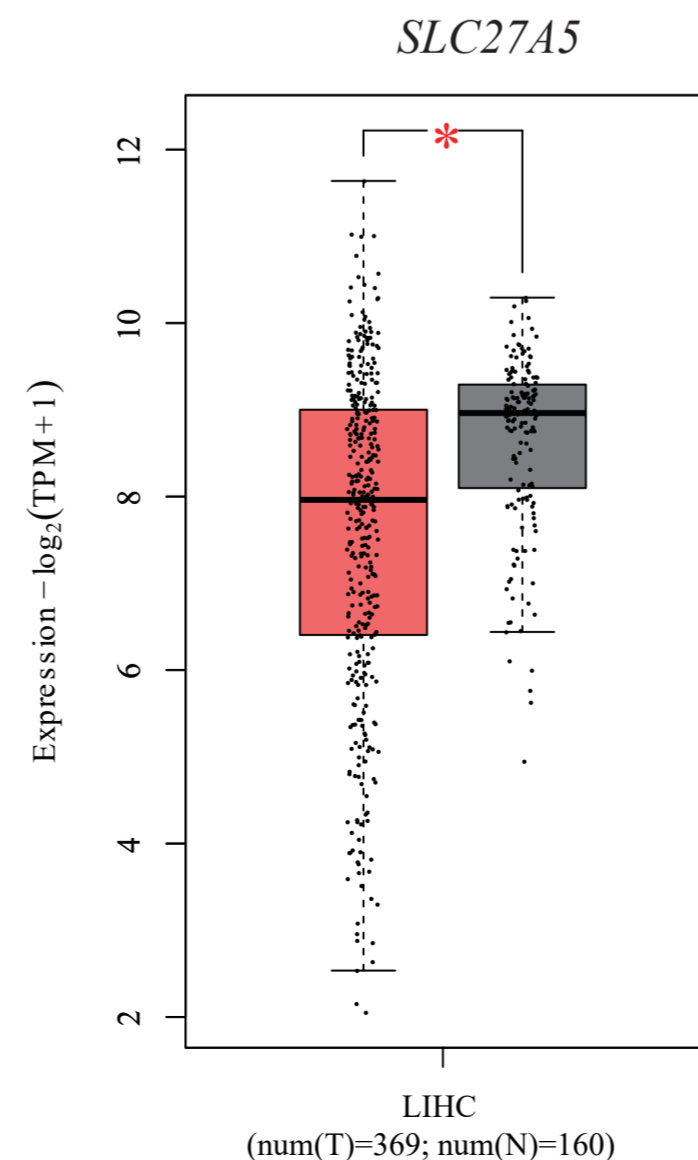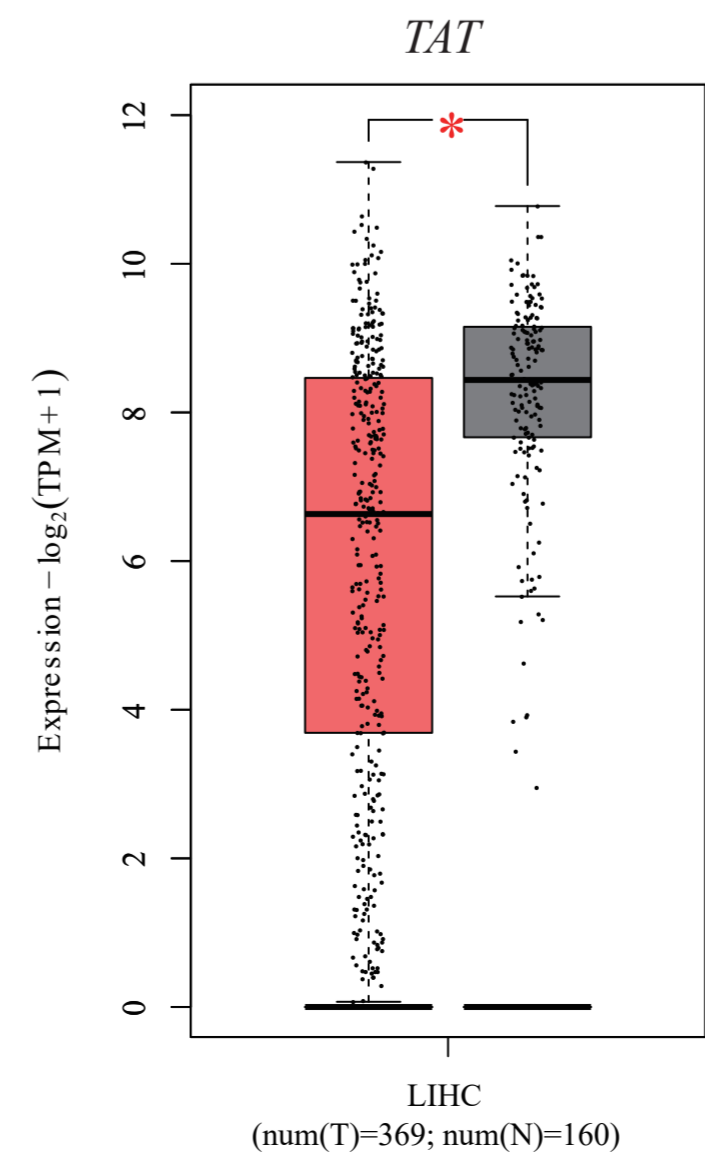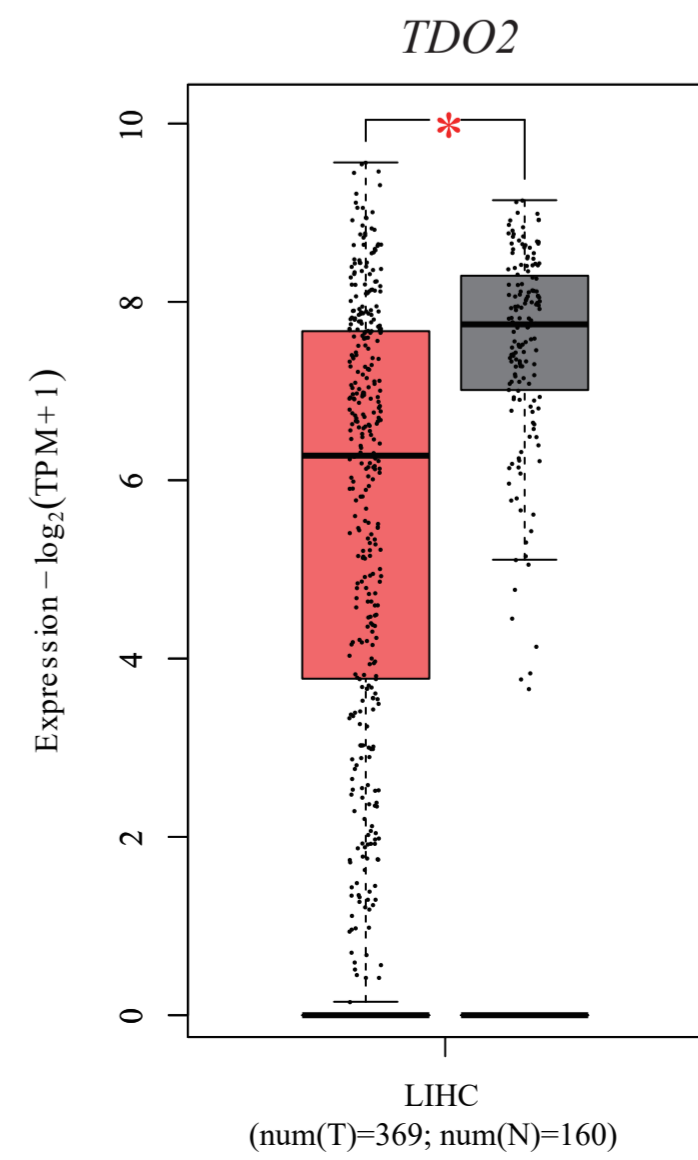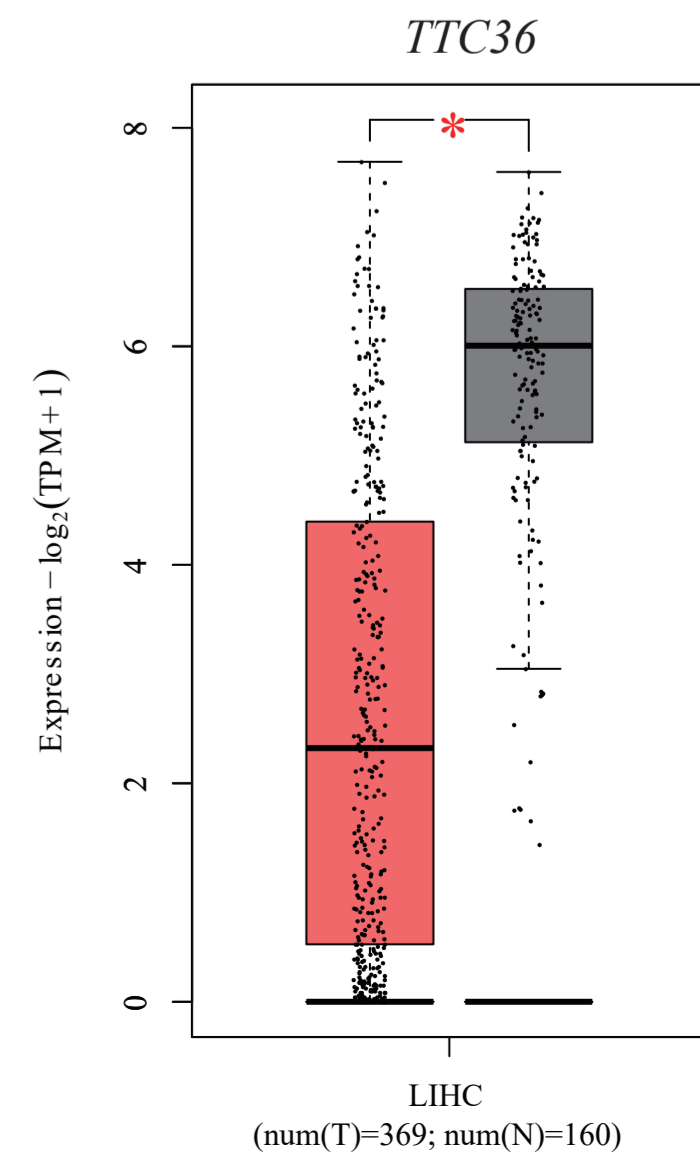

Supplement: Supplementary file 1 [file biomolecules-12-01700-s001.zip › Figure S2.pdf]

A

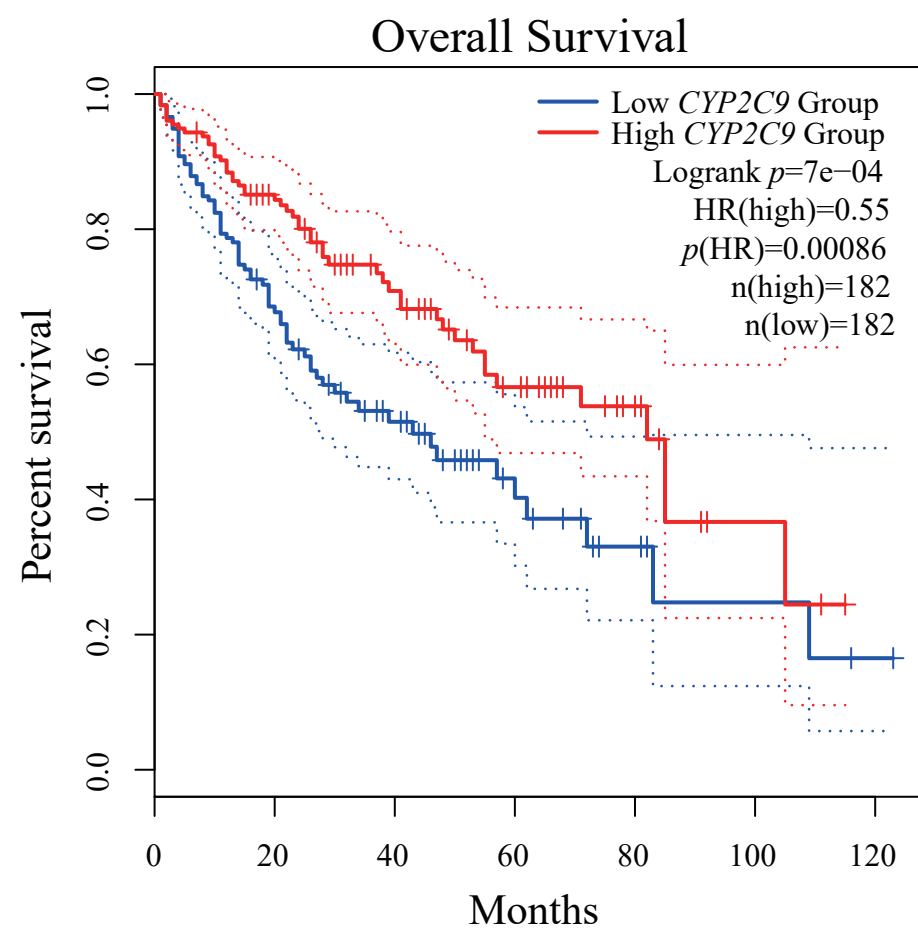

B

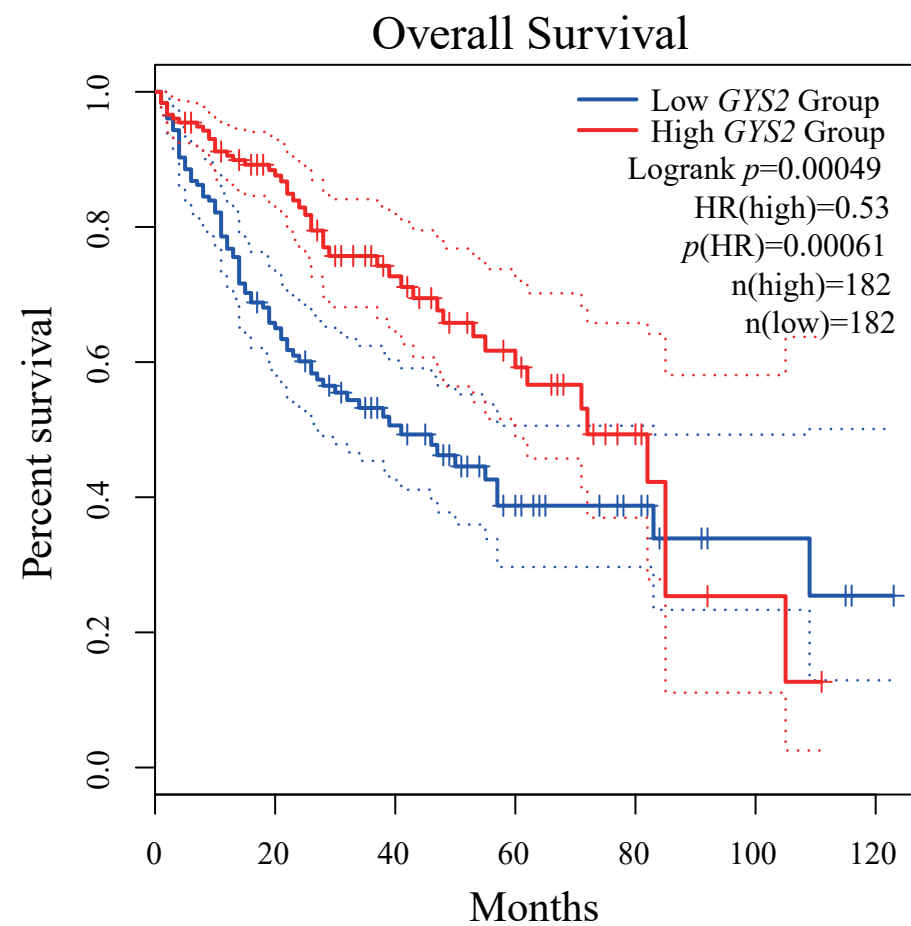

C

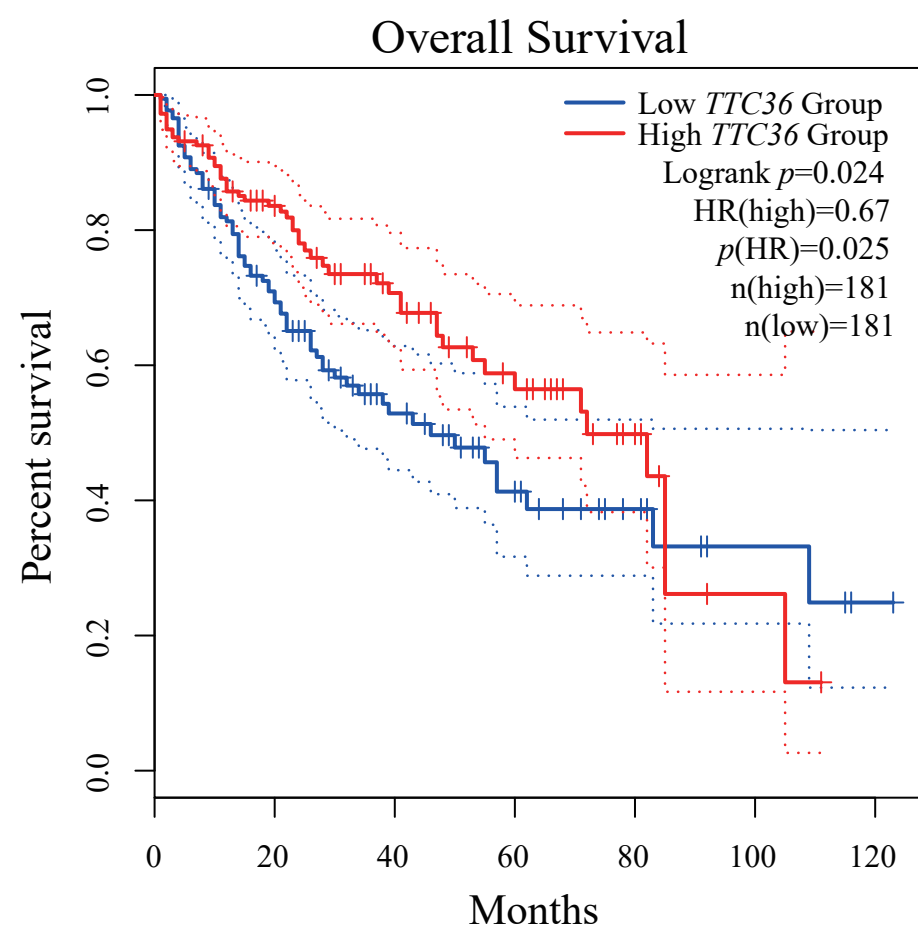

D

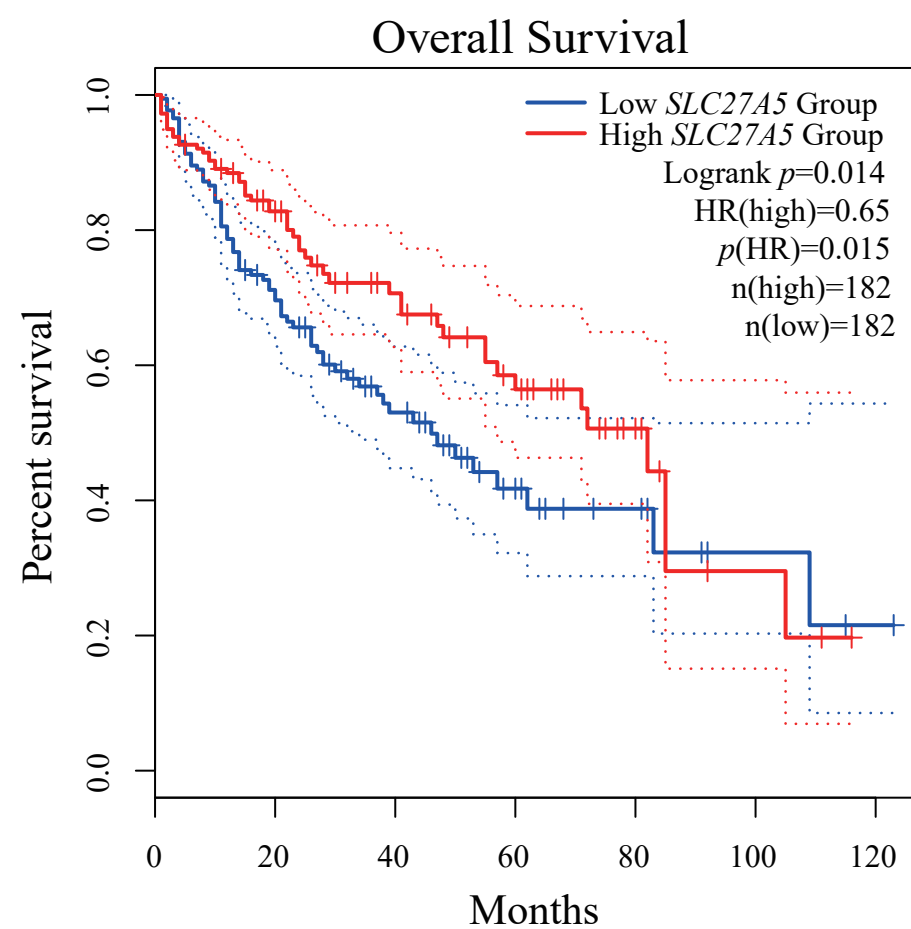

Supplement: Supplementary file 1 [file biomolecules-12-01700-s001.zip › Figure S3.pdf]

A

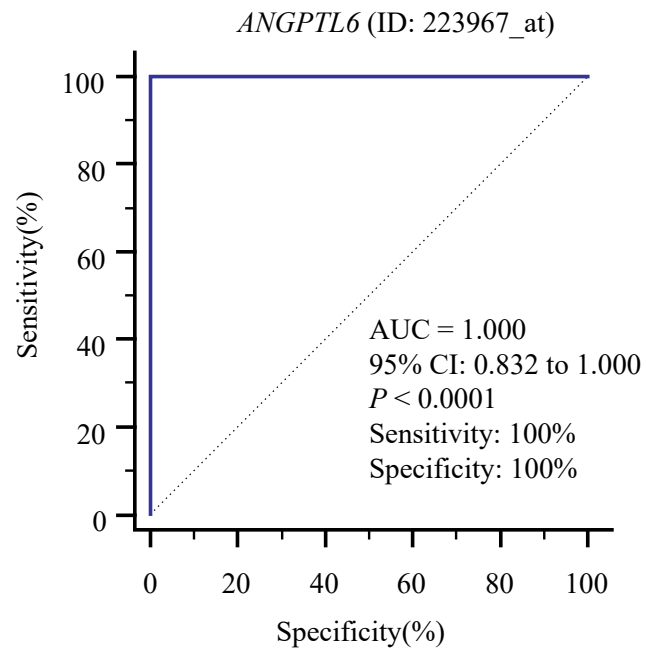

B

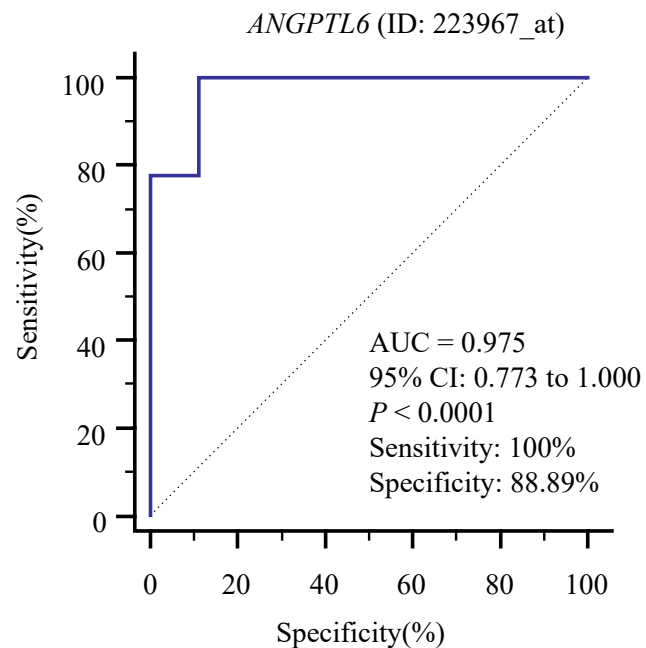

C

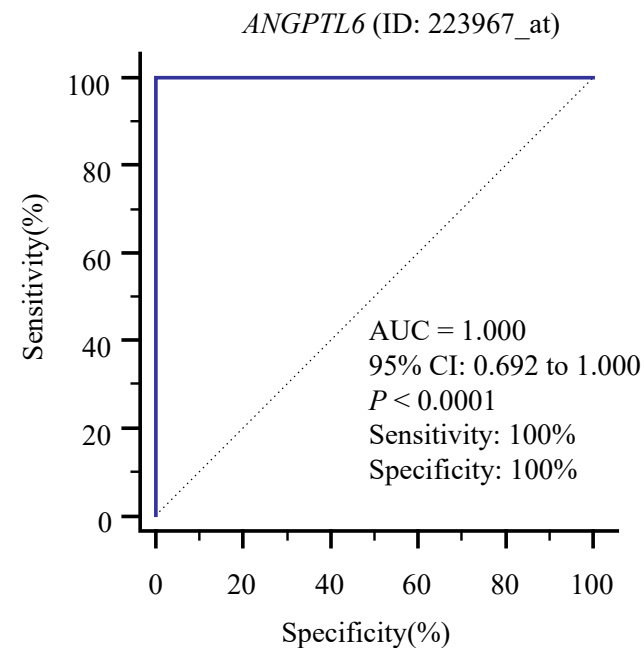

Supplement: Supplementary file 1 [file biomolecules-12-01700-s001.zip › Figure S4.pdf]

## Recurrence free survival (n=33)

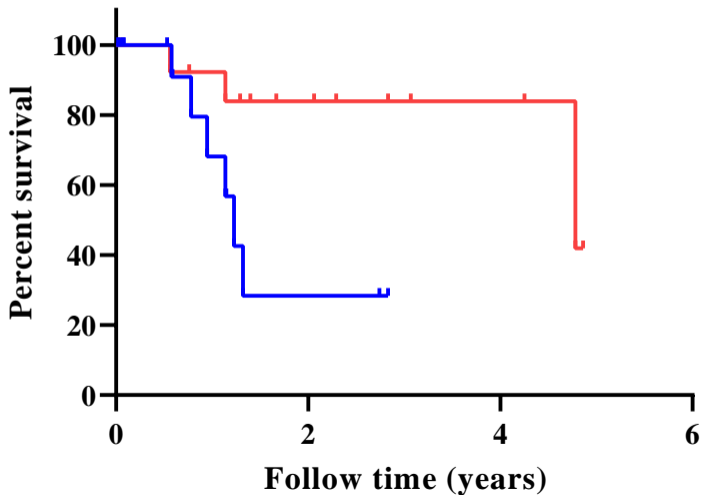

Supplement: Supplementary file 1 [file biomolecules-12-01700-s001.zip › Figure S5.pdf]
